# Precise Engineering and Efficient Biosynthesis of Robust and High‐Activity Human Haemoglobin for Artificial Oxygen Carriers

**DOI:** 10.1111/1751-7915.70128

**Published:** 2025-03-12

**Authors:** Fan Liu, Jingwen Zhou, Jianghua Li, Jian Chen, Guocheng Du, Xinrui Zhao

**Affiliations:** ^1^ Science Center for Future Foods, Jiangnan University Wuxi Jiangsu China; ^2^ Key Laboratory of Industrial Biotechnology, Ministry of Education, School of Biotechnology Jiangnan University Wuxi Jiangsu China; ^3^ Jiangsu Province Engineering Research Center of Food Synthetic Biotechnology Jiangnan University Wuxi Jiangsu China; ^4^ Engineering Research Center of Ministry of Education on Food Synthetic Biotechnology Jiangnan University Wuxi Jiangsu China; ^5^ Key Laboratory of Carbohydrate Chemistry and Biotechnology, Ministry of Education Jiangnan University Wuxi Jiangsu China

**Keywords:** heme loss, heme‐supply, high‐activity, oxygen transport efficiency, recombinant human haemoglobin, structural stability

## Abstract

Recombinant human haemoglobin (rHb) is a tetramer protein with heme as cofactors, which have extensive applications in the fields of biomaterials and biomedical therapeutics. However, due to the poor structural stability, the dissociation of heme, weak oxygen transport efficiency, and lower activity, the utilisation of rHb is severely limited in artificial oxygen carriers. Herein, based on the novel developed high‐throughput screening strategies and semi‐rational design, the engineered rHb mutant with strong stability and heme‐binding ability was obtained. In addition, through the homology alignment and rational design, the oxygen transport capacity of rHb was significantly enhanced. Furthermore, the bottlenecks of heme supply were overcome by applying the fine‐tuned heme synthesis in *Escherichia coli* . Finally, the robust and high‐activity rHb mutant was synthesised and can be used as a new generation of artificial oxygen carriers.

## Graphical abstract

The stable human haemoglobin variant was obtained through the design of novel high‐throughput screening strategies and methods. Furthermore, the supply level of heme in the production host was enhanced. Ultimately, an robust human haemoglobin was achieved with stronger stability, lower heme loss rate, lower autoxidation rate, more efficient oxygen transport capacity and higher activity.

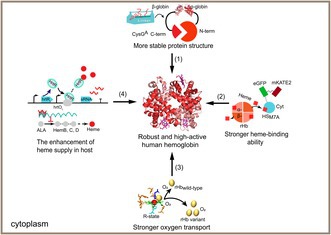

## 1 Introduction

Human haemoglobin (Hb) is a tetramer protein, including two α‐globins (141 residues) and two β‐globins (146 residues). Each globin binds to one molecule of heme, further carrying one molecule of oxygen (Nagatomo et al. 2022). Hb plays an essential role in transporting oxygen, and the efficiency is over 2‐fold higher than red blood cells, which can be used as artificial oxygen carriers to alleviate the problems of insufficient blood supply and pathogen infections (Ahmed et al. 2020). Currently, compared with the complex and time‐consuming process of extracting Hb from human blood (Jansman et al. 2022), the biosynthesized recombinant human haemoglobin (rHb) in *Escherichia coli* has many advantages, including the reduced risk of disease transmission and expensive cost (Hoffman et al. 1990). However, the heterologous expression of rHb lacks the antioxidant systems (superoxide dismutase and catalase) and 2, 3‐diphosphoglycerate (2, 3‐DPG) existing in the red blood cells, leading to severe challenges in widespread applications in artificial oxygen carriers.

The first problem of synthesised rHb is its instability that tends to dissociate into dimers and monomers when it is used alone, resulting in the degradation and serious harm to kidney (Buehler et al. 2010). Thus, the stability of rHb should be enhanced and a variety of strategies have been attempted. The chemical modifications of rHb include intramolecular cross‐linking and polymerisation with raffinose or glutaraldehyde, etc. (Jahr et al. 2019). However, these modifications will increase the cost of rHb. In addition, the single glycine linkage between two α‐globins (rHb 0.1) (Looker et al. 1992) and the mutations at special sites of rHb (α‐L29W (Maillett et al. 2008) or α‐G15A, β‐G16A/H116I (Graves et al. 2008)) have also been applied, but the improvement of rHb stability was limited due to the lack of a comprehensive design and modification approach for the overall structure of rHb (Maillett et al. 2008). To obtain more stable rHb mutants, random mutagenesis, directed evolution or rational modifications in the neglected key residues can be used to produce a great deal of potential mutants (Ren et al. 2019). Then, based on the improving fluorescent biosensors to perform high‐throughput screening, the efficient acquisition of stable rHb mutants can be achieved.

Another serious bottleneck for the application of rHb was the rapid autoxidation from the ferrous Hb to methemoglobin (Pires et al. 2023), which prevents the binding of oxygen and leads to the rapid loss of heme from rHb (Samuel et al. 2020). To address this issue, the screening of mutants with resistance to autoxidation and strong binding affinity for heme is a feasible approach. For instance, the introduction of tyrosine (β‐T84Y) as a redox mediator to prevent the oxidation process of rHb (Cooper et al. 2019), or the special mutations (β‐V67T or α‐T42V) can stabilise the binding of heme (Gattoni et al. 2001; Silkstone et al. 2016). However, these methods only relied on rational design and were suffered by limited throughput (Ren et al. 2019). At present, the real‐time monitoring of intracellular heme can be achieved by the developed heme sensors, including mAPX‐mEGFP, FRET‐based heme sensor and HrtR‐hrtO, etc. (Hanna et al. 2017; Zhang et al. 2020; Leung et al. 2021). Thus, it is feasible to realise high‐throughput modifications at the undeveloped residues and screen the heme‐binding enhanced rHb mutants by the rapid detection of heme loss rate through heme sensor.

The efficient oxygen transport capacity of rHb is especially important for the application of artificial oxygen carriers. However, due to the lack of 2, 3‐DPG, the oxygen affinity of rHb increases and it cannot effectively deliver oxygen to the tissues. The utilisation of extrinsic effectors (inositol hexaphosphate, etc.) can regulate the allosteric properties of rHb and transform rHb into a low oxygen affinity state (Ahmed et al. 2020), but the addition of these compounds will lead to increasing costs and security risks, which is not conducive to the commercial application of rHb as an artificial oxygen carrier. Additionally, the modifications of residues can also enhance the oxygen transport efficiency of rHb. For instance, the mutations at α‐V1E (Sen Gupta 2019), β‐V1M/H2_deleted_/T4I/P5A/A76K (Fronticelli and Koehler 2009) or β‐N108K (Looker et al. 1992) have been designed to lower the oxygen affinity. Furthermore, several natural hemoglobins possess the special key regions that contribute to the lower oxygen affinity (Fronticelli et al. 2002; Giardina et al. 2005) and it has been reported that the introduction of chloride ion (Cl^−^) binding sites can compensate for the absence of 2, 3‐DPG (Fronticelli et al. 2007). Hence, the integration of the above beneficial strategies can be applied to obtain a bigger breakthrough in the oxygen transport capability of rHb.

The efficient biosynthesis of high‐activity rHb is also crucial for the commercial production and large‐scale application of rHb. Although the exogenous addition of 5‐aminolevulinic acid (ALA) or heme can enhance the expression of high‐activity hemoproteins (Quehl et al. 2016), the effect of these strategies was unsatisfactory. The moderate enhancement of heme supply in the microbial hosts can overcome these limitations. In our previous research, the synthesis of holo‐hemoprotein (71.5% mol heme/mol P450 BM3_mut_) was improved by the utilisation of assembled rate‐limiting enzymes and heme‐sensitive biosensors in *E. coli* C41 (DE3) strain (Hu et al. 2023). Therefore, this heme‐supply enhanced strains may contribute to the synthesis of high‐activity rHb.

**FIGURE 1:**
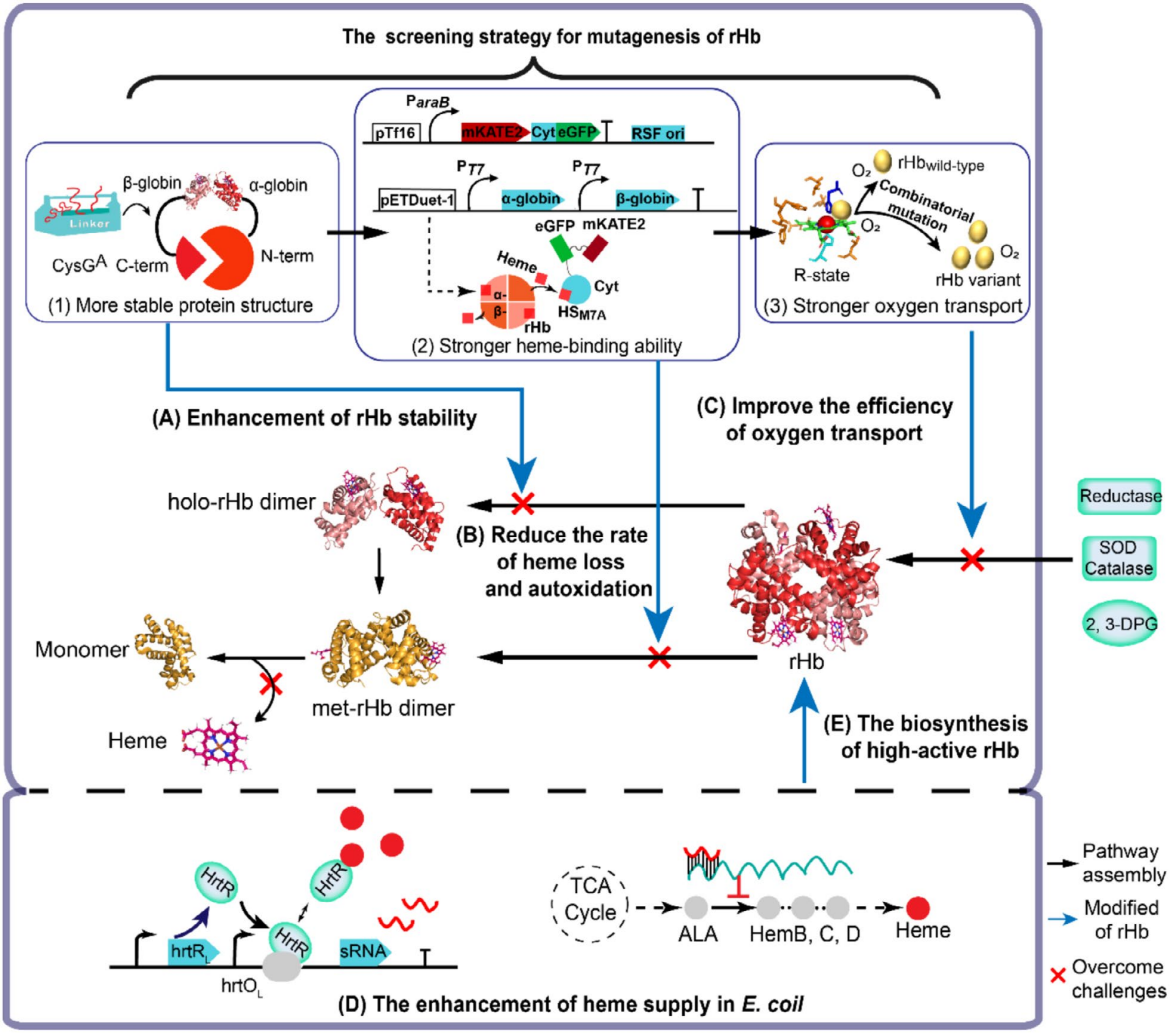
Engineering strategies to construct and synthesise robust and high‐activity rHb. (A) The design of a high‐throughput screening tool (Ren et al. 2021) through linker engineering and semi‐rational design to improve the stability of rHb (PDB ID: 2DN2) (Park et al. 2006). (B) The design of a dual‐plasmid system and semi‐rational methods for high‐throughput screening of heme‐binding enhanced rHb mutants. (C) Through rational design, the oxygen transport efficiency of rHb was enhanced. (D) The enhancement of heme supply in *E. coli* R11‐HEME strain. (E) The biosynthesis of high‐activity rHb in *E. coli* (Hu et al. 2023). ALA, 5‐aminolevulinic acid; 2, 3‐DPG, 2, 3‐diphosphoglycerate; SOD, superoxide dismutase.

In this study, the novel and efficient strategies for the modifications and biosynthesis of robust rHb were designed (Figure 1). Based on the established high‐throughput screening strategies for stable multi‐unit proteins and the mutants with enhanced heme‐binding ability, the modified rHb mutant with strong tetramer and cofactor stability was obtained. Next, the efficiency of oxygen transport was improved by the combination of homology alignment and rational design. In the following, the supplies of heme were moderately improved in the *E. coli* strain, and the biosynthesis of the superior and high‐activity rHb_13th_ mutant was achieved.

## 2 Experimental Procedures

### 2.1 Strains and Plasmids Construction

*E. coli* JM109 strain was used to construct recombinant plasmids and *E. coli* BL21(DE3) strain was used to express and modify rHb. *E. coli* C41(DE3) derivative strain HEME‐R11 was used to screen the heme‐binding enhanced rHb variants and synthesise rHb. RHb_wild‐type_ gene was synthesised from Genscript Biotech Corporation (Nanjing, China) and the Gibson assembly method was used to construct the expression cassettes controlled by pUC57, pTf16, pMAL‐c5X, pETDuet‐1 and pRSFDuet‐1 vectors. The constructed plasmids and strains were listed in Tables S1 and S2, respectively. The related primers are listed in Table S3.

### 2.2 The Establishment of High Throughput Screening Strategy for Enhanced rHb Stability

The CysG^A^ gene was amplified from the genome of BL21(DE3) strain (Ren et al. 2021) and further cloned into the pETDuet‐1 vector. The gene of rHb was inserted at the G_364_ site of CysG^A^, and different linkers (Table S4) were selected for the efficient connection between the C‐terminal of CysG^A^ and β‐globin. Based on the optimization of linkers, various rHb‐CysG^A^ fusion proteins (N‐terminal of CysG^A^‐(SSG)_2_‐α‐globin, C‐terminal of CysG^A^‐different linker‐β‐globin) were constructed. These fusion proteins were expressed in *E. coli* BL21(DE3) strains and the fluorescent values of CysG^A^ were measured at the specific wavelength (excitation wavelength 357 nm, emission wavelength 620 nm) by a microplate reader (xMark Microplate Spectrophotometer, BIO‐RAD, CA, USA) (Wildt and Deuschle 1999). The mutant with the lowest fluorescent intensity of CysG^A^ was selected as the candidate for the stability modification of rHb. The six reported rHb mutants with different degrees of stability were constructed by Gibson assembly (Table S5). According to the correlation between fluorescent intensities of rHb‐CysG^A^ fusion proteins and stability mutant energies of rHb mutants, the most suitable rHb‐CysG^A^ biosensor was determined for the enhanced stability of rHb.

### 2.3 The Establishment of High‐Throughput Screening Strategy for Enhanced Heme‐Binding Ability of rHb

The HS1_M7A_ (Hanna et al. 2016) was subcloned into pTf16 (p15A *ori*) or pTf16 (RSF *ori*) vector by Gibson assembly. The recombinant plasmids and pETDuet‐1 plasmid harbouring rHb_wild‐type_ were further co‐transformed into the heme‐supply enhanced *E. coli* HEME‐R11 strain, respectively. Thus, two strains with different dual‐plasmid expressional systems (rHb‐HS1_M7A_) were obtained. Then these two strains were cultured in TB medium (50 mg/L ampicillin and 17 mg/L chloramphenicol) and were induced using 40 mM IPTG and 10 mM arabinose until the value of OD_600_ reached 0.6 (220 rpm). The strains were cultured at 30°C for 5 h, and the cells were washed twice with 20.0 mM PBS buffer (pH 7.4).

The fluorescent ratios of eGFP/mKATE2 in these cells were respectively measured (eGFP: excitation wavelength 488 nm, emission wavelength 523 nm; mKATE2: excitation wavelength 588 nm, emission wavelength 620 nm). The dual‐plasmid system with the strongest fluorescent ratio of eGFP/mKATE2 was selected to enhance the heme‐binding capacity of rHb. In addition, seven reported rHb mutants with different degrees of heme‐binding capacity were constructed by the pETDuet‐1 vector. The binding mutation energies between these mutants and heme were determined through Discovery Studio 2019 (Accelrys Inc., San Diego, CA, USA) (Table S6). The screening efficiency of the dual‐plasmid system was verified based on the correlation between seven rHb mutants‐HS1_M7A_ and their binding mutation energies. Furthermore, to accelerate the rate of heme loss from rHb, different reagents were respectively added to the fermentation broth with expressed rHb‐HS1_M7A_, including cuprous chloride (1.0, 10.0, 100.0, 1000.0 μM), cetyltrimethylammonium bromide (2.0, 20.0, 200.0, 2000.0 μM), SDS (50.0, 500.0, 2000.0 μM) and acid alcohol (1.0, 5.0, 10.0 μL). The reagent and rHb‐HS1_M7A_ were incubated at 30°C for 5 h (220 rpm). The most suitable heme dissociation reagent for rHb‐HS1_M7A_ was selected based on the reduction in fluorescent ratio of eGFP/mKATE2 compared with the control without adding any reagent.

### 2.4 The Semi‐Rational Design Strategies to Screen the Stable rHb Variants

According to the structural and functional characteristics of rHb, the mutation sites were screened from four regions, including: (1) the intersubunit contact regions of rHb (34 sites in α1β1 interface and 19 sites in α1β2 interface); (2) the eight sites with B‐factor values higher than 40 calculated by PyMOL (DeLano Scientific LLC, CA, USA); (3) the surface residues of rHb (33 sites in α‐globin and 30 sites in β‐globin); (4) the potential 15 sites with increased stability through alanine scanning (Stability Mutation Energy < −0.40 kcal/mol). The potential key sites (Stability Mutation Energy< −2.00 kcal/mol) were further examined using virtual saturation mutagenesis through Discovery Studio 2019 (Accelrys Inc., San Diego, CA, USA).

In the following, the hot sites were determined for the stability modification of rHb. At first, the mutations constructed by the NNK degenerate bases were expressed in *E. coli* BL21(DE3) strain. Next, the best mutant with the strongest fluorescent value of CysG^A^ was selected as the starting point for the sequential rounds of mutations. Based on the ratio of CysG^A^ fluorescent intensity (> 1.2) between rHb mutants and rHb_wild‐type_, the candidate sites were determined for iterative saturation mutations.

### 2.5 The Semi‐Rational Design Strategies to Screen the Heme‐Binding Enhanced rHb Variants

The mutation sites were screened from two regions: (1) the sites within 4 Å of the heme pocket (α‐ and β‐globin); (2) the sites in the CE helix of β‐globin. The potential key sites (Binding Mutation Energy < −2.0 kcal/mol) were examined using the Calculate Mutation Energy (Binding) module of Discovery Studio 2019 (Accelrys Inc., San Diego, CA, USA). The mutations constructed by the NNK degenerate bases were expressed in *E. coli* R11‐HEME strain. Then, based on the fluorescent ratios of eGFP/mKATE2 between the mutants (> 1.5‐fold) and the control (rHb_6th_), the hot sites were confirmed for iterative saturation mutations to obtain the heme‐binding enhanced rHb variants.

### 2.6 Iterative Saturation Mutagenesis

To improve the stability of rHb, the saturation mutagenesis of hot sites was performed utilising NNK degenerate bases and the plasmids harbouring rHb mutants were transformed into *E. coli* BL21(DE3) strain. Then, at least 100 clones for each key site were selected to verify the effect of mutations. Each single colony was further transferred to a 96‐well plate containing 0.2 mL LB (100 mg/L ampicillin) medium per well and cultivated in a Microtron microplate oscillator (INFORS, Switzerland) at 37°C (220 rpm). The strains were induced by 10.0 μM IPTG until the value of OD_600_ reached 0.6 and 20.0 mg/L heme was supplemented for the synthesis of rHb. The strains were cultured at 30°C for 10 h (220 rpm) and the cells were washed twice with 20.0 mM PBS buffer (pH 7.4). The CysG^A^ fluorescent values of these cells were measured and the highest CysG^A^ fluorescent value of the mutant in each round of iterative saturation mutagenesis was selected. The next round of saturation mutagenesis was further performed for the best mutant until the fluorescent values of ideal mutants were nearly the same between two rounds of iterative saturation mutagenesis.

As for the enhancement of heme‐binding ability for rHb, the saturation mutagenesis of hot sites was performed by NNK degenerate bases and the plasmids harbouring rHb mutants were transformed into *E. coli* R11‐HEME strain, and at least 100 clones for each key site were selected to verify the effect of mutations. The single colony was inoculated into a 96‐well plate containing 0.2 mL TB (50 mg/L ampicillin, 17 mg/L chloramphenicol) medium per well and cultivated at 37°C (220 rpm) until the value of OD_600_ reached 0.6. The strains were induced using 40 mM IPTG and 10 mM arabinose and cultured at 30°C for 5 h (220 rpm). Next, 0.05 mM SDS was added into the fermentation broth to accelerate heme loss from rHb. The strains were sequentially cultured at 30°C for 8 h (220 rpm) and the cells were washed twice with 20.0 mM PBS buffer (pH 7.4). The eGFP/mKATE2 fluorescent ratios of these cells were measured and the highest fluorescent ratio of the mutant in each round of iterative saturation mutagenesis was selected. The next round of saturation mutagenesis was further performed for the best mutant until the fluorescent values of ideal mutants were nearly the same between the two rounds of iterative saturation mutagenesis.

### 2.7 Homology Alignment of Various Hemoglobins to Enhance the Oxygen Transport Capacity of rHb

The sequences of hemoglobins with lower oxygen affinity (cow, goat, deer, sheep, camel, mouse, cat and bat) (Giardina et al. 2005; Mairbäurl and Weber 2012) were obtained from the National Center for Biotechnology Information (https://www.ncbi.nlm.nih.gov/). Multiple sequence alignments were performed using the Do Complete Alignment module of ClustalW (European Bioinformatics Institute, www.ebi.ac.uk/clustalw/). The specially reported sites related to the oxygen affinity were selected to enhance the oxygen transport capacity of rHb. The features of these sites include the lower homology between rHb and most hemoglobins with lower oxygen affinity.

## 3 Results

### 3.1 Establish High‐Throughput Screening Strategies for Improving the Stability of rHb

**FIGURE 2:**
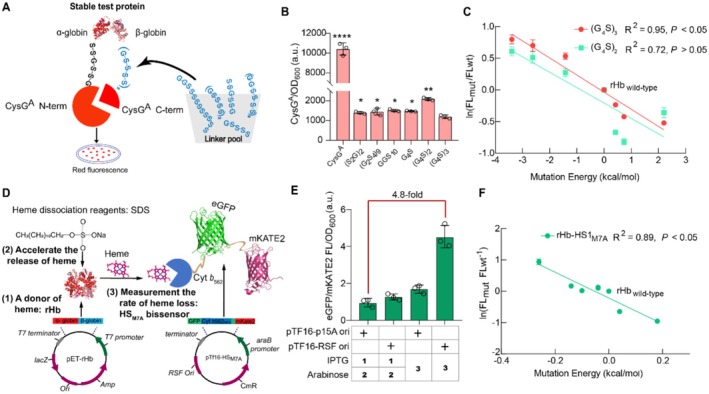
High‐throughput screening strategies for the continuous modifications in rHb stability. (A) The selection of rHb variants with strong stability through high‐throughput screening strategy. The linker between the β‐globin and C‐terminus of CysG^A^ was optimised from the linker pool. (B) Red fluorescent intensity of rHb‐CysG^A^ fusion proteins with different linkers. (C) Correlation analysis between the fluorescent intensity of rHb‐CysG^A^ fusion proteins and the mutational energy of mutants. Red line represents the (G_4_S)_3_ linker that connects C‐terminus of β‐globin and C‐terminus of CysG^A^, and green line represents (G_4_S)_2_ linker that connects C‐terminus of β‐globin and C‐terminus of CysG^A^. From the left to the right part, the used strains were pET‐rHb_α‐G15A/β‐G16A/β‐H116I_, pET‐rHb_α‐G15A/β‐G16A_, pET‐rHb_α‐G15A_, pET‐rHb_wild‐type_, pET‐rHb_β‐L28W_, pET‐rHb_α‐L29W_, pET‐rHb_α‐P119S_, respectively. (D) The construction of high‐throughput screening strategy for heme‐binding enhanced of rHb variants (PDB ID of Hb: 2DN2, PDB ID of red fluorescent protein: 3PJ5, PDB ID of green fluorescent protein: 2GX2). (E) The eGFP/mKATE2 fluorescence of rHb‐HS1_M7A_ system under the control of different replicons (RSF *ori* and p15A *ori*) or induction conditions. 1, 2 represents inducer (IPTG) is first added, followed by the addition of arabinose. Three represents the addition of two inducers (IPTG and arabinose) at the same time. (F) The correlation analysis between the fluorescent ratio of rHb‐HS1_M7A_ and the mutational energy of variants. From the left to the right part, the used strains were pET‐rHb_β‐S44H_, pET‐rHb_β‐K82D_, pET‐rHb_β‐T84Y_, pET‐rHb_β‐F41Y_, pET‐rHb_β‐V67T_, rHb_wild‐type_, pET‐rHb_α‐H58L_, pET‐rHb_β‐H92Q_, respectively. Data was presented as mean value ± SD from three independent biological replicates (*n* = 3) and Pearson correlation was applied to verify the relationship between the fluorescent intensity and mutational energy of variants. Statistical evaluation was performed through a two‐tailed *t*‐test (*p* value). *p < 0.05, **p < 0.01, ****p < 0.0001.

To efficiently achieve the modifications in rHb stability, including enhancing the assembly of the tetrameric structure and reducing the rate of heme loss, it is imperative to establish suitable high‐throughput strategies for the screening of stable mutants. A truncated siroheme synthase (CysG^A^) can convert uroporphyrin III into precorrin‐2, emitting bright red fluorescence in the cells (Bryant et al. 2020). Thus, tripartite folding sensors of monomer protein (CysG^A^_(G4S)2_) for stability screening have been established through inserting the protein of interest (POI) at the G_364_ site of CysG^A^ (N‐terminal of CysG^A^‐(SSG)_2_‐POI‐(GGGGS)_2_‐C‐terminal of CysG^A^) (Ren et al. 2021). However, when using multi‐subunit rHb as the POI, the correlation between CysG^A^ fluorescent values and stable mutation energies of rHb mutants in rHb‐CysG^A^_(G4S)2_ was insufficient (*R* ^2^ = 0.72, *p* > 0.05, Figure 2C) to screen stable rHb (Figure 2C, Table S5). According to the previous reports, the replacement of the linker will significantly affect the correlation of the POI and sensors (Malik et al. 2014). Thus, the modifications in the linker were crucial to overcoming the interference in the multi‐domain fusion proteins (rHb‐CysG^A^) (Chen et al. 2013).

To verify the effect of linker on the rHb variants, rHb‐CysG^A^_(G4S)2_ (N‐terminal of CysG^A^‐(SSG)_2_‐α‐globin, C‐terminal of CysG^A^‐(GGGGS)_2_‐β‐globin) was used as the reference. As the shorter linker (SSG)_2_ can promote the effective coupling of unstable α‐globin (Grawe and Stein 2021), only the linker between the C‐terminal of CysG^A^ and β‐globin was designed based on different lengths (5–54 bp) and the percent of glycine (33.0%–80.0%) (Figure 2A, Table S4); the variation in the percent of glycine affects the flexibility of linkers, which is conducive to designing more rational linkers to avoid protein misfolding and reduce steric hindrance (Choi et al. 2015; Rosmalen et al. 2017). The results showed that the fluorescent intensity of rHb‐CysG^A^_(G4S)3_ (N‐terminal of CysG^A^‐(SSG)_2_‐α‐globin, C‐terminal of CysG^A^‐(GGGGS)_3_‐β‐globin) is the lowest (Figure 2B), and it was an ideal starting point to screen more stable rHb mutants. Subsequently, six reported rHb mutants with different degrees of stability were selected, including three mutations with better stability (α‐G15A, α‐G15A/β‐G16A and α‐G15A/β‐G16A/β‐H116) and three mutations with weaker stability (α‐P119S, α‐L29W and β‐L28W) (Wiltrout et al. 2005; Giordano et al. 2007; Graves et al. 2008). Based on the molecular simulation tool (Discovery Studio 2019), the stability mutation energies of six mutants were determined (α‐P119S > α‐L29W > β‐L28W > wild‐type > α‐G15A > α‐G15A/β‐G16A > α‐G15A/β‐G16A/β‐H116I, Table S5). Furthermore, to verify the reliability of the folding sensor, the correlation between the fluorescent intensity of six rHb mutants‐CysG^A^_(G4S)3_ fusion protein and their stability mutation energy was investigated. The correlation of rHb‐CysG^A^_(G4S)3_ (*R* ^2^ = 0.95, *p* < 0.05, Figure 2C) was significantly higher than that of rHb‐CysG^A^_(G4S)2_ (*R* ^2^ = 0.72, *p* > 0.05, Figure 2C), indicating that the linker (G4S)_3_ is more suitable for the expression of fusion protein between the multi‐subunit of POI (rHb) and CysG^A^, which promotes the recovery of CysG^A^ enzymatic activity and the reemission of the fluorescent signal (Rosmalen et al. 2017). In other words, the optimised rHb‐CysG^A^_(G4S)3_ folding sensor can decrease the high proportion of false positives in the previous screening strategies (chloramphenicol acetyltransferase) (Seitz et al. 2007; Ren et al. 2019) and it is an ideal high‐throughput screening tool for evaluating the stability of multi‐subunit proteins.

Besides the instability of subunit assembling, the rate of heme loss in rHb was rapid because of the faster autoxidation (Kassa et al. 2016). Currently, due to the trace and transient nature of heme loss in the synthesis of rHb, the method to select the protein with the enhanced capacity for heme‐binding cannot meet the demand for high‐throughput screening. Thus, a unique dual‐plasmid screening system (rHb‐HS1_M7A_) was designed. In this system, the synthesis of rHb was performed by the pETDuet‐1 plasmid in the previously constructed HEME‐R11 strain with the enhanced level of heme synthesis (Hu et al. 2023), facilitating synthesised rHb to rapidly capture the intracellular heme. In addition, several reagents (SDS and cuprous chloride, etc.) were utilised to accelerate the dissociation rate of bound heme from rHb. Furthermore, the loss of heme was measured using the heme‐binding domain of cytochrome *b*_562_ from the HS1_M7A_ heme sensor expressed by the pTf16 plasmid that is induced by L‐arabinose and could coexist with the pETDuet‐1 plasmid in *E. coli* . The degree of heme loss was visualised through the fluorescent ratio (the heme‐sensitive eGFP and the insensitive mKATE2) (Xue et al. 2023) (Figure 2D).

Based on the characteristics of rHb‐HS1_M7A_, a higher fluorescent ratio of eGFP/mKATE2 indicates a rHb mutant with stronger heme‐binding capacity. However, the fluorescent ratio of eGFP/mKATE2 only reached 1.0 for rHb‐HS1_M7A_ controlled by pTf16 (p15A *ori*). To increase the response range, the lower copy p15A *ori* was replaced with the higher copy RSF *ori*, leading to the increase of the fluorescent ratio to 1.3. In addition, the induction condition of the system was optimised by simultaneously inducing both pETDuet‐1 (rHb_wild‐type_) and pTf16‐HS1_M7A_ (RSF *ori*), leading to a 4.8‐fold increase in the fluorescent ratio of eGFP/mKATE2 (Figure 2E). Furthermore, when 50.0 μM SDS was added, the fluorescent ratio of eGFP/mKATE2 for different rHb (rHb_wild‐type_ and rHb_β‐K82D_) decreased by 39.8% ± 2.5% and 41.9% ± 4.0%, indicating that SDS could accelerate the dissociation rate of heme loss from rHb (Figure S1). Finally, seven reported rHb mutants with different degrees of heme‐binding capacity were selected, including five mutants with enhanced heme‐binding capacity (β‐V67T, β‐F41Y, β‐T84Y, β‐K82D and β‐S44H) and two mutants with reduced heme‐binding capacity (β‐H92Q and α‐H58L) (de Weinstein et al. 2000; Varnado et al. 2013; Bisse et al. 2017). Using the molecular simulation tool (Discovery Studio 2019), the binding mutation energies between the seven mutants and heme were determined (β‐H92Q > α‐H58L > wild‐type > β‐V67T = β‐F41Y > β‐T84Y > β‐K82D > β‐S44H, Table S6). To verify the reliability of the dual‐plasmid screening system that seven rHb mutants and HS1_M7A_ were respectively co‐expressed, the correlation between the fluorescent ratio of eGFP/mKATE2 and the binding mutation energies of these mutants was investigated. The results showed that the correlation of rHb‐HS1_M7A_ reached 0.89 (*p* < 0.05) (Figure 2F), demonstrating that rHb‐HS1_M7A_ can be efficiently used to screen the rHb mutant with enhanced heme‐binding capacity.

### 3.2 The Improvement of rHb Stability by Semi‐Rational Design and Multi‐Round High‐Throughput Screening

**FIGURE 3:**
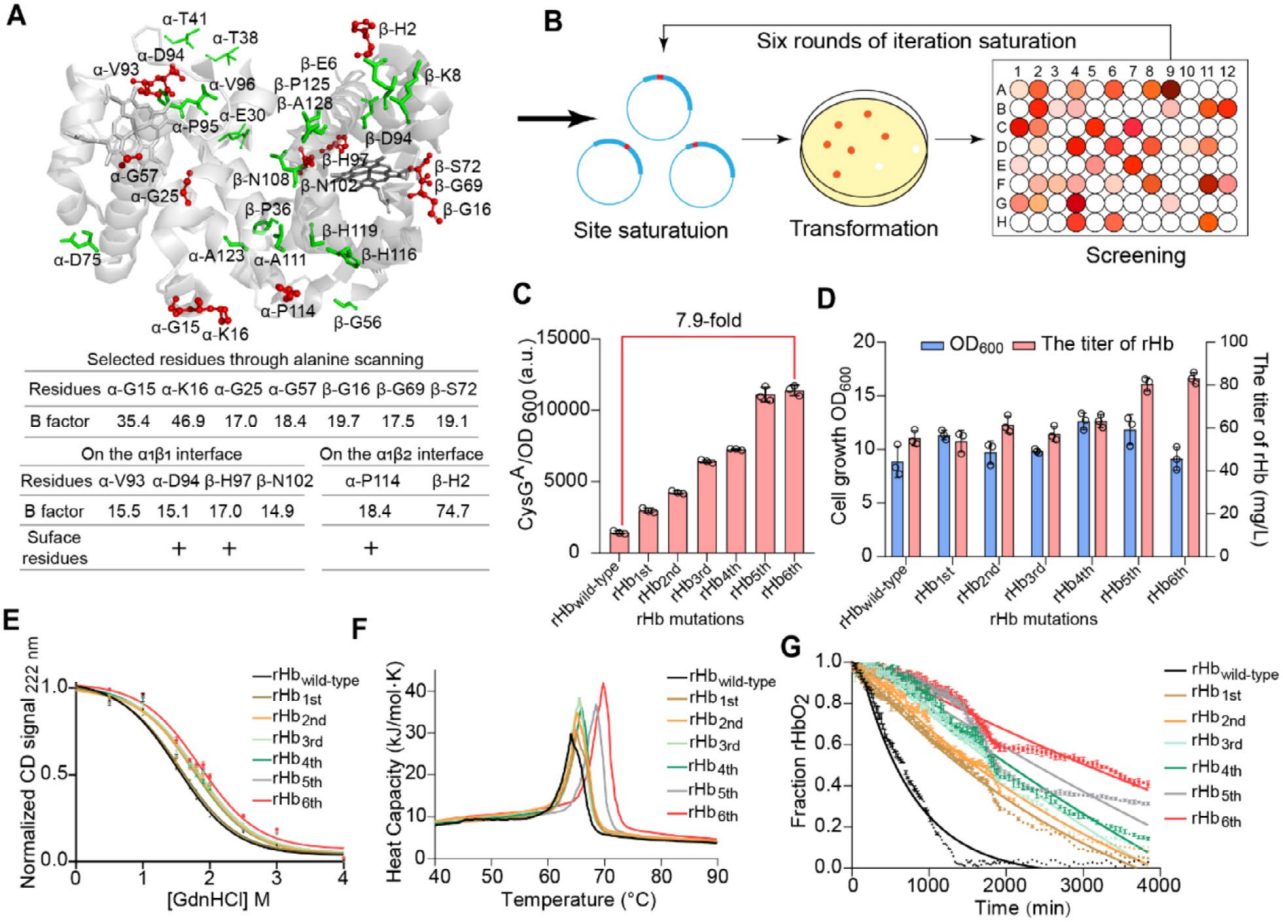
The design and modifications in rHb to improve its stability. (A) The determination of mutation sites for the improvement of rHb stability (PDB ID: 2DN2) by semi‐rational design. The green amino acids represent the candidates after virtual saturation mutagenesis and the red amino acids were ultimately used for the iterative saturation mutagenesis. (B) The process of iterative saturation mutagenesis. (C‐G) The properties of ideal mutants determined by each round of iterative saturation mutagenesis: (C) fluorescent intensity, (D) biomass and the titre of rHb, (E) secondary structure of mutants determined by circular dichroism (CD) spectroscopy, (F) thermal stability of mutants determined by differential scanning calorimetry (DSC) data, (G) the rate of autoxidation for rHb mutants. Data was expressed as mean value ± SD from three independent biological replicates (*n* = 3).

After the high‐throughput screening methods for the stable rHb were established, it is crucial to determine the key mutation sites to improve rHb stability. Based on the structural characteristics of rHb, several appropriate mutation sites can be efficiently determined by semi‐rational design. The selection of mutation sites for rHb was considered from four aspects: (1) the intersubunit contacts of α1β1 interface (34 amino acid residues), the intersubunit contacts of α1β2 interface (19 residues amino acid residues) (Samuel et al. 2017, 2020); (2) the surface sites of rHb (33 residues in α‐globin and 30 residues in β‐globin); (3) the residues with a B‐factor value higher than 40 (possible unstable sites, 8 sites) (Figure S2A); (4) potential amino acids that improve rHb stability (Stability Mutation Energy < −0.4 kcal/mol, 15 residues) through alanine scanning. Among all these residues, the sites with specific functions were removed, including the Bohr effect (α‐H45, β‐L141 and β‐H146) (Okonjo 2017) and the allosteric function (α‐K99, α‐R141), etc. (Ahmed et al. 2020). Taking the important influencing factors into consideration, 31 key residues were selected based on the mutations with significantly enhanced stability (Stability Mutation Energy < −2.0 kcal/mol) through virtual saturation mutagenesis (Figure S2B). To further enhance the efficiency of screening, a saturated mutation library was constructed utilising the NNK degenerate bases for the 31 key residues of rHb. Based on the ratio of CysG^A^ fluorescent intensity between the mutants (> 1.2) and control (rHb_wild‐type_), 13 residues were identified as the “hot spots” related to the stability of rHb (Figure 3A, Figure S2B), which can be subsequently used for the iterative saturation mutations (Figure 3B). In addition, the CysG^A^ fluorescent intensity of α‐P114I (rHb_1st_) was highest (1.8‐fold) compared with the value of rHb_wild‐type_ (Figure S2B), which means the mutant of α‐P114I is a proper starting variant for the following iterative saturation. The α‐114 site in rHb is located on the flexible loop region between the G and H helix of Hb, which has a significant effect on the stability of α subunit (Domingues‐Hamdi et al. 2014).

To obtain the ideal rHb mutants, a mutation library was constructed in the first round of iterative saturation for the 13 “hot spots” residues (Figure S2B) and 100 clones for each residue were screened to achieve 95% coverage in the library (Reetz et al. 2010). After six rounds of iteration saturation, the CysG^A^ fluorescent intensity of rHb_6th_ (α‐G25C/G57K/P114I, β‐H2E/H97W/N102T) was 7.9‐fold higher than the value of rHb_wild‐type_ (Figure 3C) and was similar to the value of rHb_5th_ (α‐G25C/G57K/P114I, β‐H2E/N102T), which indicates that an ideal stable rHb (rHb_6th_) was obtained. Meanwhile, the oxygen affinities of all ideal mutants in each iteration were checked to ensure their future applications in the field of artificial oxygen carriers (Figure S2C,D).

To further validate the applicable properties of rHb_6th_, its secondary structure, thermal stability, and autoxidation rate were detected, respectively. The 222 nm signal value of Circular dichroism (CD) was used to examine the secondary structure of rHb and guanidine hydrochloride (GdnHCl) was used as the denaturant for rHb (Samuel et al. 2015; Kanagarajan et al. 2021). The [GdnHCl]_midpoint_ of rHb_6th_ (the concentration of GdnHCl when 50% secondary structure dissociated) increased to 1.9 M, which was 26.7% higher than the value of rHb_wild‐type_ (1.5 M) (Figure 3E). In addition, the melting temperature (TM) of rHb_6th_ increased to 69.7°C ± 0.1°C, which was 8.9% higher than the value of rHb_wild‐type_ (64.0°C ± 0.1°C) (Figure 3F). The improvement of thermal stability in rHb is beneficial to the intracellular synthesis and long‐term storage at room temperature (Bhomia et al. 2016; Giuriato et al. 2024). Moreover, the rate of autoxidation for rHb_6th_ (*k*_autox_ = 0.01 ± 0.01 h^−1^) was reduced, which was 14.8‐fold lower than the rate for rHb_wild‐type_ (*k*_autox_ = 0.17 ± 0.03 h^−1^) (Figure 3G, Table S7), indicating that rHb_6th_ has good resistance to the peroxidation (Strader et al. 2017). Furthermore, due to the improvement of stability, the titre of rHb_6th_ (83.2 ± 1.5 mg/L) is 1.5‐fold higher than the value of rHb_wild‐type_ (Figure 3D). Therefore, compared with rHb_wild‐type_, rHb_6th_ possess significant advantages in stability, oxidation resistance and higher titre for commercial production and application.

### 3.3 The Mechanism Analysis of rHb Stability

**FIGURE 4:**
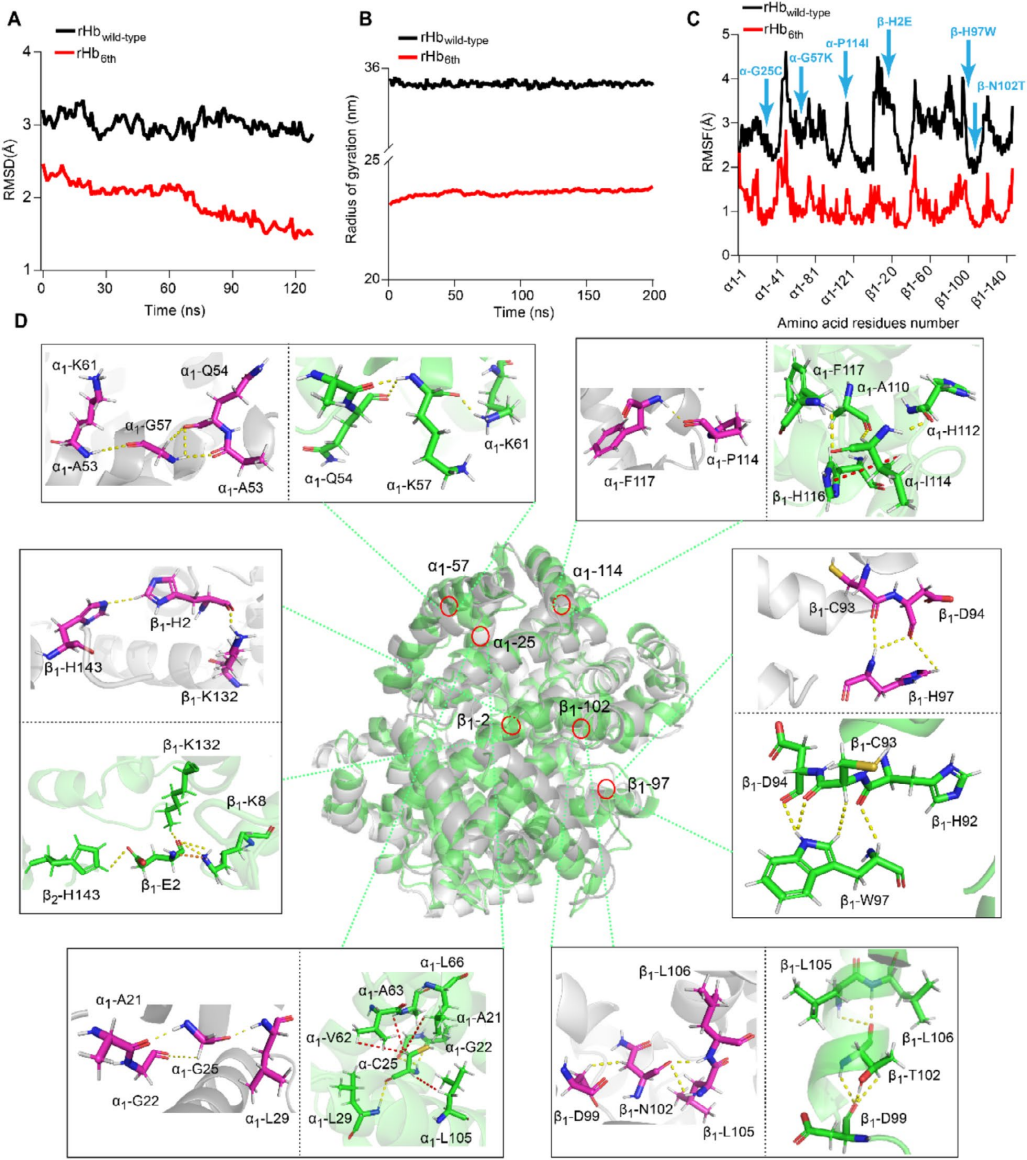
The mechanism for the improvement of rHb stability. (A) The results of RMSD for the rHb_wild‐type_ and rHb_6th_. (B) Compared with rHb_wild‐type_, radius of gyration in rHb_6th_. (C) The results of RMSF fluctuation for the rHb_wild‐type_ and rHb_6th_. (D) The non‐covalent bonds formed among the mutated amino acids in rHb_6th_ compared with the original amino acids in rHb_wild‐type_ (PDB ID: 2DN2) (yellow dotted line: Hydrogen bonding interaction, red dotted line: Hydrophobic interaction, orange dotted line: Electrostatic force).

To elucidate the mechanism for the improvement of rHb stability, the molecular dynamics (MD) simulations of rHb_wild‐type_ and rHb_6th_ were carried out based on the CHARMm force. The results showed that the average (1.9 Å) and final (1.5 Å) RMSD values of rHb_6th_ were significantly lower than the values of rHb_wild‐type_ (average value: 3.0 Å, final value: 2.9 Å) (Figure 4A). Meanwhile, the average radius of gyration in rHb_6th_ (23.7 ± 0.1 nm) is lower than the radius of gyration of rHb_wild‐type_ (35.8 ± 0.0 nm), indicating a tighter structure in rHb_6th_ (Figure 4B). Especially, in the regions of mutation sites, the RMSF value of rHb_6th_ was significantly lower than the value of rHb_wild‐type_, including the range of 5–40 (α‐G25C), 50–70 (α‐G57K), 101–130 (α‐P114I) sites for α‐globin, the range of 1–35 (β‐H2E), 94–120 (β‐H97W and β‐N102T) sites for β‐globin (Figure 4C, Figure S3A). It suggests that the structural rigidity of rHb_6th_ was significantly enhanced. Moreover, the hydrogen bonds (61 pairs), hydrophobic (11 pairs) and electrostatic (15 pairs) interactions in rHb_6th_ were respectively increased by 6.0%, 3.1% and 34.1% (Figure S3B), which means the differences in interacting forces are the crucial factors for the stability of rHb.

Moreover, the interaction forces of mutated sites with surrounding residues were analysed (Figure 4D, Figure S3C). The results showed that the formed numbers of non‐covalent bonds at the α‐G25C site of rHb_6th_ are 2.3‐fold higher than the numbers in rHb_wild‐type_, leading to the formation of an extra 4 pairs of hydrophobic interactions (between α‐C25 and α‐V62/α‐A63/α‐L66/α‐L105 residues). Similarly, the formed numbers of non‐covalent bonds at the α‐P114I site of rHb_6th_ increased by 4.0‐fold compared to the numbers in rHb_wild‐type_, including an extra 1 pair of hydrophobic interactions (between α‐I114 and β‐H116 residues) and 2 pairs of hydrogen bonds (between α‐I114 and α‐A110/α‐H112 residues). In addition, the mutation at the βH2E site increases an extra 1 pair of electrostatic interactions (between β‐E2 and β‐K8 residues) and 2 pairs of hydrogen bonds (between β‐E2 and β‐K8 residues). Moreover, the mutation at the β‐N102T site also increases 2 pairs of hydrogen bonds (between β‐T102 and β‐D99 residues). The increase in interaction forces can significantly promote the folding efficiency of globin (Dias et al. 2010). While the interaction forces at the α‐G57K and β‐H97W sites did not significantly change (Figure 4D) and the modifications at these two sites resulted in a rapid decrease in the surrounding RMSF (the range of 50–70 sites in α‐globin, 94–120 sites in β‐globin) (Figure 4C), which is related to the remote intervention on the secondary structure of rHb (Yu and Dalby 2018).

### 3.4 The Enhancement of Heme‐Binding Capacity for rHb by Semi‐Rational Design and Multi‐Round High‐Throughput Screening

**FIGURE 5:**
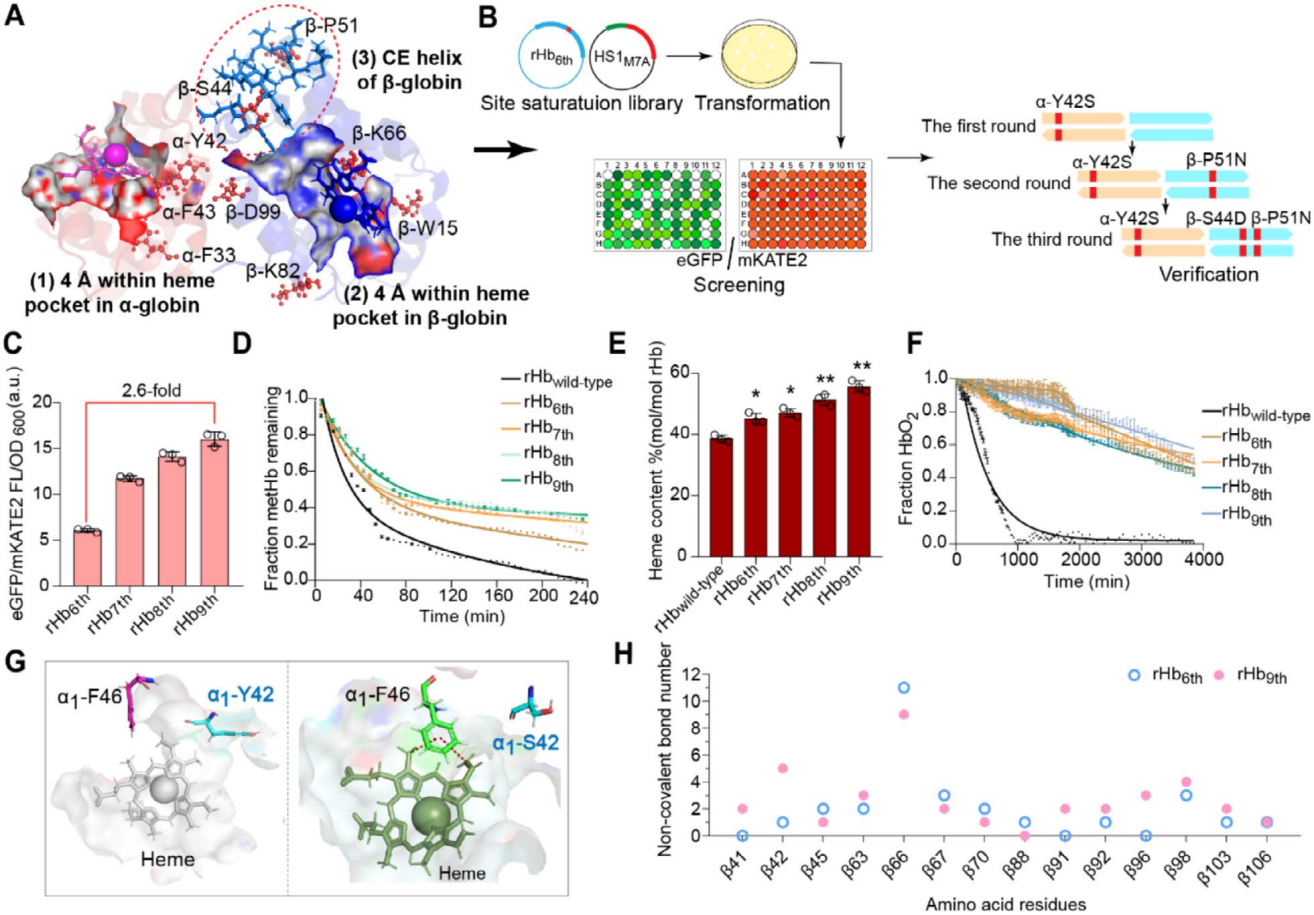
The enhanced heme‐binding capacity of rHb. (A) The strategy to select proper mutation sites for enhancing heme‐binding strength. The residues (black) were chosen for iterative saturation mutagenesis after virtual screening (PDB ID: 2DN2). (B) The process of iterative saturation mutagenesis and the information of ideal mutants after each round of mutagenesis. (C) Fluorescent intensities of ideal mutants in each round of iterative saturation mutagenesis. (D–F) Compared with rHb_wild‐type_, the properties of ideal mutants in each round of iterative saturation mutagenesis: (D) the rate of heme loss, (E) the binding rate of heme, (F) the rate of autoxidation. (G) Compared with rHb_6th_, the formed non‐covalent bonds at the mutated residues with significant changes in rHb_9th_. Yellow dotted line: Hydrogen bonding interaction, red dotted line: Hydrophobic interaction, orange dotted line: Electrostatic force. (H) The number of non‐covalent interactions related to heme in rHb_6th_ and rHb_9th_. Data were presented as mean value ± SD from three independent biological replicates (*n* = 3). Statistical evaluation was performed through a two‐tailed *t*‐test, compared to the rHb_wild‐type_ control (*p* value). *p < 0.05, **p < 0.01.

The constructed rHb‐HS1_M7A_ system was used to screen the rHb mutants with enhanced heme‐binding capacity. To identify suitable residues for the modifications by semi‐rational design, the virtual saturation mutagenesis was performed on the residues within 4 Å around the heme pocket in both α and β subunits of rHb_6th_, as well as on the C and E helices that affect heme binding in β‐globin (Samuel and Case 2020). Then, 26 sites were selected as candidates based on the mutations with significantly enhanced heme affinity (Binding Mutation Energy < −0.6 kcal/mol) through virtual saturation mutagenesis (Figure S4A). In addition, to narrow down the screening range, saturation mutagenesis was performed on all the 26 alternative residues, and nine sites further were chosen for the following iterative saturation mutagenesis based on the fluorescent ratio of eGFP/mKATE2 between the mutants (> 1.5‐fold) and control (rHb_6th_) (Figure 5A, Figure S4A). After three rounds of iterative saturation mutagenesis (Figure 5B), rHb_9th_ (α‐G25C/Y42S/G57K/P114I, β‐H2E/S44D/P51N/H97W/N102T) showed a 2.64‐fold increase in the fluorescent ratio of eGFP/mKATE2 compared to the value of rHb_6th_, which was only 13.5% higher than the value of rHb_8th_ from the second round (Figure 5C). Additionally, the CysG^A^ fluorescent intensity of rHb_9th_ was 3.6% higher than that of rHb_6th_, demonstrating that the following mutations did not affect the stability of rHb_6th_ (Figure S4B), and rHb_9th_ was an ideal mutant with significantly enhanced heme‐binding capacity.

Furthermore, the region of the heme pocket in rHb_9th_ had been changed, leading to a significant decrease in the rate of heme loss (*k*_‐H_ β = 1.32 ± 0.10 h^−1^, *k*_‐H_ α = 0.02 ± 0.004 h^−1^) by 1.4‐fold (β‐globin) and 6.0‐fold (α‐globin) compared to the value for rHb_6th_ (*k*_‐H_ β = 1.85 ± 0.25 h^−1^, *k*_‐H_ α = 0.12 ± 0.02 h^−1^), respectively (Figure 5D, Table S8). The enhancement of heme‐binding capacity in rHb_9th_ is also beneficial for improving the titre of rHb to 84.9 ± 2.7 mg/L, the binding rate of heme to 55.7% ± 1.8% mol heme/mol rHb (Figure 5E, Figure S4A) and reducing the rate of autoxidation to (*k*_autox_ = 0.01 ± 0.00 h^−1^) (Figure 5F, Table S8), while there was no significant difference in the other properties between rHb_9th_ and rHb_6th_ (Figure S4C–G).

As for the effect of mutations in rHb_9th_ on the interaction forces between these residues, the number of non‐covalent bonds associated with heme was obviously increased (Figures 5G–H and S4H,I and S5), which is an important factor to reduce the rate of heme loss. For instance, the α‐Y42S mutation that locates at located within the 4 Å region of heme pocket can enhance the binding ability between heme and nearby α‐F46 site, forming extra 2 pairs of hydrophobic interactions in rHb_9th_ (Figure 5G). In addition, the sites of β‐S44 and β‐P51 are located in the CE helical region that is more flexible than α‐globin due to the presence of 6 additional residues and contributes a lot in the faster rate of heme loss in β‐globin (Bisse et al. 2017). The β‐P51N and β‐S44D mutations increase the number of non‐covalent bonds related to the heme‐binding by 32.1%, significantly reducing the flexibility in the CE helix of β‐globin (Figure 5H).

### 3.5 Enhancing the Oxygen Transport Capacity of rHb Through the Combined Rational Design Strategies

Due to the lack of 2, 3‐DPG in red blood cells, the oxygen affinity of rHb (*P*_50_ = 14.0 mmHg) is higher than the value of native Hb (*P*_50_ = ~27.0 mmHg), leading to the reduced release of oxygen in tissues and severely affecting the efficiency of oxygen transport. Although the oxygen affinities of previously modified rHb1.1 (*P*_50_ = 35.0 mmHg) and rHb 2.0 (*P*_50_ = 33.0 mmHg) can partly meet the practical needs (Hermann et al. 2007; Meng et al. 2018), the challenges remain in commercial applications due to the various adverse effects of these mutants. Therefore, it is necessary to obtain rHb mutants with the capacity for efficient oxygen transport. The ideal rHb mutant should possess a similar property of oxygen transport to red blood cells and maintain the improved stable characteristics of rHb_9th_.

First, the effect of reported beneficial mutations needs to be verified in rHb_9th_. These modifications include: (1) the unique residues in haemoglobin from deer mice contributing to the lower oxygen affinity (Storz et al. 2007). The key sites of α‐K60G/A71G were modified in rHb_9th_ to construct rHb_10th_ (α‐G25C/Y42S/G57K/P114I/K60G/A71G, β‐H2E/S44D/P51N/H97W/N102T). (2) the special N‐terminal region related to the low oxygen affinity in cow β‐globin, the Cl^−^ binding site of β‐A76K that can make up the lack of 2,3‐DPG and the ideal rHb mutant (β‐V1M/H2_deleted_/T4I/P5A/A76K) (Fronticelli et al. 2002). The rHb_11th_(α‐G25C/Y42S/G57K/P114I, β‐H2E/S44D/P51N/H97W/N102T/V1M/H2_deleted_/T4I/P5A/A76K) was constructed. Additionally, to verify the impact of β‐H2E deletion on the stability of rHb, the rHb_12th_ (α‐G25C/Y42S/G57K/P114I, β‐H2E/S44D/P51N/H97W/N102T/V1M/T4I/P5A/A76K) was constructed. (3) The mutation at the surface residue (α‐V1E) associated with stability and oxygen affinity (Bellelli and Brunori 2020) and the modification in β‐N108K that can introduce a new Cl^−^ binding site (Tsai and Ho 2002). The rHb_13th_ (α‐G25C/Y42S/G57K/P114I/V1E, β‐H2E/S44D/P51N/H97W/N102T/N108K) was constructed.

Besides the reported available sites, more potential sites are waiting to be explored by the sequence alignments between rHb_wild‐type_ and eight hemoglobins with low oxygen affinities, including: (1) the differences (β‐T4S/P5G) in the key sites between rHb_wild‐type_ and bat haemoglobin that possesses the special region for its low oxygen affinity (Giardina et al. 2005). The rHb_14th_ (α‐G25C/Y42S/G57K/P114I, β‐H2E/S44D/P51N/H97W/N102T/T4S/P5G/A76K) was constructed. (2) The impact of methionine at 0 position of β‐globin. The rHb_15th_ (α‐G25C/Y42S/G57K/P114I, β‐H2E/S44D/P51N/H97W/N102T/0M_inserted_/T4S/P5G/A76K) was constructed. (3) The key surface amino acid (β‐H116). As the mutation at β‐H116L obviously increased the oxygen affinity (Wajcman et al. 2003), the other modifications in β‐H116 are promising to realise the decrease in oxygen affinity. Through homologous alignment, the arginine residue frequently appears at β‐H116 site in the hemoglobins with low oxygen affinities. Thus, the rHb_16th_ (α‐G25C/Y42S/G57K/P114I, β‐H2E/S44D/P51N/H97W/N102T/H116R) was constructed.

**FIGURE 6:**
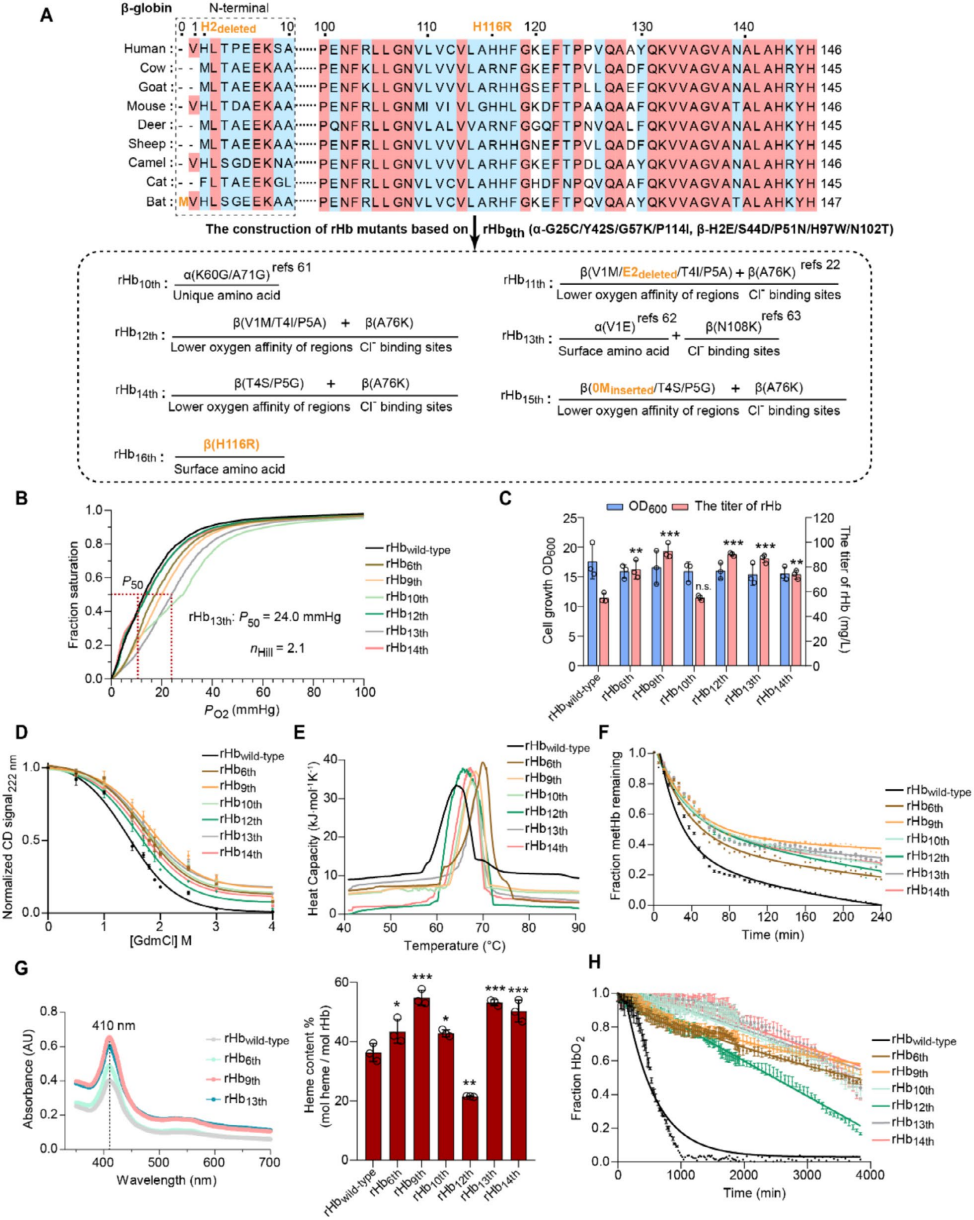
The enhancement of oxygen transport for rHb. (A) Homology alignment of sequences between rHb_wild‐type_ and eight hemoglobins with lower oxygen affinities. The red and blue background indicates the conserved residues and partially conserved residues, respectively. The orange font represents the conserved residues selected for the modification of oxygen transport capacity. The refs 22, 61–63 mean the modifications of sites that determined based on literatures. (B) The oxygen equilibrium curve of mutants. (C‐H) The properties of rHb variants with modified capability of oxygen transport: (C) the biomass and the titers of rHb, (D) the secondary structures of mutants determined by CD spectroscopy, (E) the thermal stabilities of mutants determined by DSC data, (F) the rates of heme loss, (G) the results of full‐wavelength scan (350–700 nm) and the binding rates of heme, (H) the rates of autoxidation for rHb mutants. Data was presented as mean value ± SD from three independent biological replicates (*n* = 3). Statistical evaluation was performed through a two‐tailed t‐test, compared to the rHb_wild‐type_ control (*p* value). *p < 0.05, **p < 0.01, ***p < 0.001. n. s. represents no statistical significance (*p* ≥ 0.05).

In a word, seven new versions of rHb were designed on the basis of rHb_9th_ (Figure 6A, Figure S6A), and the effect of these mutations on the expression and stability of rHb_9th_ was first tested. Among them, three variants (rHb_11th_, rHb_15th_ and rHb_16th_) were abandoned due to the significantly lower fluorescent intensities of CysG^A^ and eGFP/mKATE2 (Figure S6B). Next, the methionine aminopeptidase and five rHb variants (rHb_9th_, rHb_10th_, rHb_12th_, rHb_13th_ and rHb_14th_) were co‐expressed in *E. coli* BL21(DE3) strain to validate their oxygen affinities and cooperativities, respectively (Natarajan et al. 2020). Compared to rHb_wild‐type_, the oxygen equilibrium curve of rHb_9th_ shifted to the right and the value of *P*_50_ increased from 13.1 ± 0.2 mmHg to 20.1 ± 0.3 mmHg, demonstrating that the oxygen affinity was reduced due to the optimization of the heme pocket region. In addition, the oxygen affinity of rHb_13th_ (*P*_50_ = 24.0 ± 0.2 mmHg) is significantly reduced and the cooperative binding ability of rHb_13th_ (Hill coefficient (*n*_Hill_) = 2.1 ± 0.1) was stronger than that of rHb_wild‐type_ (*n*_Hill_ = 1.8 ± 0.1) and rHb_9th_ (*n*_Hill_ = 1.4 ± 0.1) (Figure 6B, Table S9).

Except for the oxygen affinity, the other excellent properties in rHb_9th_, including the titre of rHb, the stability and heme‐binding capacity, were maintained in rHb_13th_ (Figure 6C–G and S6C, Table S9). In particular, although the autoxidation rate of rHb_13th_ (*k*_autox_ = 0.02 ± 0.00 h^−1^) was a little higher than the rate of rHb_9th_ (*k*_autox_ = 0.01 ± 0.00 h^−1^), the value still was reduced by 13.9‐fold compared to the value of rHb_wild‐type_ (*k*_autox_ = 0.22 ± 0.05 h^−1^) (Figure 6H, Table S9). Therefore, rHb_13th_ has a good potential to be used as an artificial oxygen carrier, laying a foundation for following modifications and commercial applications.

### 3.6 The Synthesis of Stable and High‐Activity rHb in Prokaryotic Microorganism

**FIGURE 7:**
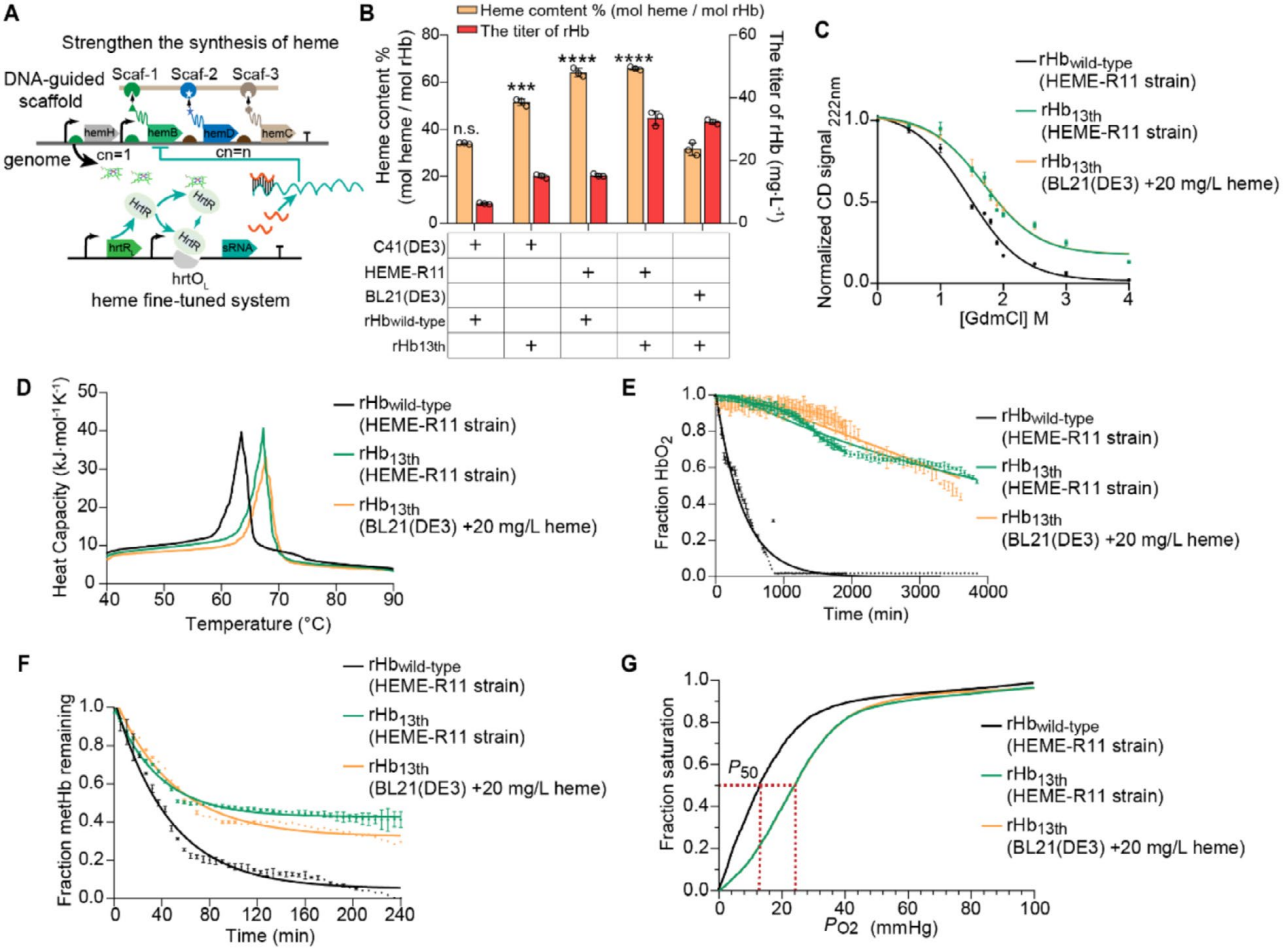
The synthesis of stable and high‐activity rHb in *E. coli* . (A) The *E. coli* strain with enhanced and fine‐tuned heme supply (Hu et al. 2023). (B) The binding rates of heme and the titers of rHb_wild‐type_ and rHb_13th_ were verified in different kinds of *E. coli* hosts. (C‐G) Compared with the values of rHb_wild‐type_, the properties of rHb_13th_ were analysed in HEME‐R11strain with enhanced heme supply, including: (C) the secondary structure of mutants determined by CD spectroscopy, (D) the thermal stability of mutants determined by DSC data, (E) the rates of autoxidation for rHb mutants, (F) the rates of heme loss, (G) the oxygen equilibrium curve of rHb mutants. Data was presented as mean value ± SD from three independent biological replicates (*n* = 3). Statistical evaluation was performed through a two‐tailed t‐test (*p*‐value). ***p < 0.001, ****p < 0.0001, n. s. represents no statistical significance (*p* ≥ 0.05).

In previous research, the higher titre of rHb and shorter fermentation period were achieved in the model microorganism *E. coli* . Thus, *E. coli* was widely applied in the study of rHb properties. As the higher proportion of active hemoproteins contributes to their physiological and biochemical functions, the enhancement of intracellular heme is beneficial to synthesise stable and high‐activity rHb (Yu et al. 2024). To determine the appropriate hosts for the synthesis of rHb, rHb_13th_ was expressed in *E. coli* BL21(DE3) and C41(DE3) strains, respectively. Although the titre of rHb_13th_ in C41(DE3) was lower than the titre in BL21(DE3), the proportion of active rHb_13th_ (51.5% ± 1.4% mol heme/mol rHb) was 1.6‐fold higher than the proportion in BL21(DE3)‐rHb_13th_ (31.5% ± 2.7% mol heme/mol rHb) (Figure 7B), indicating that C41(DE3) was more suitable to synthesise active rHb. Among C41(DE3) derived strains (Hu et al. 2023), the HEME‐R11 strain with the enhanced heme synthetic pathway and fine‐tuned heme biosensor was an ideal platform to synthesise rHb_13th_ (Figure 7A). The titre of rHb_13th_ (33.5 ± 2.2 mg/L) in the HEME‐R11 strain increased by 2.2‐fold compared to the titre in C41(DE3), and the binding rate of heme (65.9% ± 0.7% mol heme/mol rHb) increased by 1.3‐fold (Figure 7B, Figure S7A,B).

In addition, the stability, autoxidation rate, rHb titers and oxygen transport capability of rHb_13th_ in the HEME‐R11 strain were similar to those of the rHb_13th_ obtained in BL21(DE3) control strain with the addition of 20 mg/L heme (Figure S7B–G, Table S10), such as the titre of rHb_13th_ was increased by 2.2‐fold compared to rHb_wild‐type_ in the HEME‐R11 strain. Furthermore, the rate of heme loss (*k*_‐H_ α = 0.02 ± 0.00 h^−1^) of rHb_13th_ in the HEME‐R11 strain was reduced by 66.7% compared to rHb_13th_ in the control strain with the addition of 20 mg/L heme (Figure 7F, Table S10). Therefore, the superior properties of rHb_13th_ in the HEME‐R11 strain can provide a good material for its future medical applications.

## 4 Discussion

Due to the advantages of safety and convenience, the modifications in rHb are a promising approach to promote its applications in the preparation of stable and reliable artificial oxygen carriers (Li et al. 2023). However, there are several drawbacks that exist in the current rHb variants, including the instability of the tetramer, the loss of heme, the inefficient capability of oxygen transport, and the lower activity of synthesised rHb (Ma et al. 2023). The ideal rHb mutant requires rational design and high‐throughput screening methods to meet commercial availability. However, the effect of currently reported strategies is unsatisfactory. To address this challenge, new high‐throughput screening strategies for improving the structural stability and heme‐binding capacity of rHb were developed in this study. In addition, based on rational molecular simulation, the multi‐properties of rHb were stepwise enhanced, including the stability of the tetramer structure, the affinity of heme‐binding, the resistance to auto‐oxidation and the efficiency of oxygen transport. Furthermore, the supply of the crucial cofactor (heme) was moderately increased in the *E. coli* strain. Finally, the high‐activity and robust rHb_13th_ mutant (α‐V1E/G25C/Y42S/G57K/P114I, β‐H2E/S44D/P51N/H97W/N102T/N108K) was obtained in the *E. coli* strain.

In previous research, diverse biosensors have been widely used in high‐throughput screening and modifications of protein stability. For example, the enhancement of thermal stability for imidazole glycerol phosphate synthase through chloramphenicol acetyltransferase reporter (Liu et al. 2020), the optimization of folding ability for 8‐barrel proteins using a β‐lactamase tripartite reporter (Wang et al. 2017) and the improvement of stability for SteT protein by a split GFP reporter, etc. (Rodríguez‐Banqueri et al. 2016). However, these biosensors have some defects in the higher rate of false‐positive results and poor compatibility, which are not suitable for the engineering of rHb stability. Thus, based on the fluorescent sensor CysG^A^ and optimised linker, we designed the biosensor rHb‐CysG^A^_(G4S)3_, combining it with the construction of an available dual‐plasmid system (rHb‐HS1_M7A_) to achieve the high‐throughput screening of stable rHb mutants. This rapid and universal strategy can be further applied in the enhancement of protein stability with multi‐subunits and can efficiently eliminate a large amount of negative mutants that affect rHb stability for the following rHb modifications (Benitez et al. 2019). For instance, the stable rHb_6th_ mutant was effectively screened by rHb‐CysG^A^_(G4S)3_ and its excellent stability was continuously detected and maintained in the final rHb_13th_ mutant during the subsequent improvements of other properties. Moreover, since the CysG^A^ gene was widely used in various organisms (Ren et al. 2021) and HS1_M7A_ is capable of functioning in both prokaryotic and eukaryotic systems (Hanna et al. 2016), the strategy can be applied to other hosts.

Furthermore, dependent on the heme supply to enhance *E. coli* strain R11‐HEME, the high‐activity rHb_13th_ mutant (65.9% ± 0.7% mol heme/mol rHb) was obtained. Interestingly, the enhancement of structural stability and heme‐binding stability in rHb_13th_ compared rHb_wild‐type_, the binding rate of heme (51.5% ± 1.4% mol heme/mol rHb) and rHb titers (15.1 ± 0.6 mg/L) increased by 1.5‐fold and 2.4‐fold in C41(DE3) (Figure 7B). This advantage of rHb_13th_ has greater tolerance to the harsh growth conditions (such as heme‐deficient), further demonstrating its potential as an ideal artificial oxygen carrier. Besides, the engineering strategy in this study also contributes to addressing the potential challenges in the production of rHb, such as optimising the storage stability in vitro to promote the commercial application of rHb (Savla and Palmer 2022), and stabilising the extracellular secretory expression of rHb to achieve the higher titre (Besada‐Lombana and Da Silva 2019).

## 5 Conclusion

In this study, to obtain an ideal structurally stable rHb mutant, the high‐throughput screening tool Hb‐CysG^A^_(G4S)3_ was designed through linker engineering. To obtain rHb variants with enhanced heme‐binding ability, the corresponding high‐throughput screening tool rHb‐HS1_M7A_ was designed through the optimisation of expression systems and expression elements for heme detection. To enhance the oxygen transport efficiency of rHb, a homology sequence alignment was conducted between haemoglobin with low oxygen affinity and rHb; the key amino acid sites that influence the oxygen transport efficiency were identified.

To improve the supply level of the cofactor heme for haemoglobin in the production host, furthermore, the application of the heme‐supply enhanced *E. coli* strain HEME‐R11 resulted in the overall improvement of rHb properties and biosynthesis, which was successfully accomplished in *E. coli* , including stronger stability and heme‐binding strength, lower autoxidation tendency, more efficient oxygen transport efficiency and higher titre and activity of rHb. The constructed and synthesised rHb_13th_ will pave the way for the practical applications of rHb as an artificial oxygen carrier and will benefit the optimisation of relevant properties of other medical proteins.

## Author Contributions

**Fan Liu:** investigation, validation, writing – original draft, writing – review and editing. **Jingwen Zhou:** conceptualization. **Jianghua Li:** conceptualization. **Jian Chen:** conceptualization. **Guocheng Du:** conceptualization. **Xinrui Zhao:** conceptualization, methodology, writing – review and editing, writing – original draft, funding acquisition.

## Conflicts of Interest

The authors declare no conflicts of interest.

## Supporting information


Appendix S1.

## Data Availability

The authors state that data supporting the results of this study are included in this paper and Supporting Information. All data are available from the corresponding author upon request. Source data are provided in this article.
