# Supplementary material for: Precise Engineering and Efficient Biosynthesis of Robust and High‐Activity Human Haemoglobin for Artificial Oxygen Carriers
Source: Microb Biotechnol. 2025 Mar 12;18(3):e70128. doi: 10.1111/1751-7915.70128 (PMC11900719; doi:10.1111/1751-7915.70128)
Supplement: Supplementary file 1 — Appendix S1. [file MBT2-18-e70128-s001.docx]

**Supplementary information**

**Precise engineering and efficient biosynthesis of robust and high-activity human hemoglobin for artificial oxygen carriers**

Fan Liu^1,2,3,4^, Jingwen Zhou^1,2,3,4^, Jianghua Li^1,2,3,4^, Jian Chen^1,2,3,4^, Guocheng Du^1,2,3,4,5^, Xinrui Zhao^1,2,3,4*^

^1^ Science Center for Future Foods, Jiangnan University, 1800 Lihu Road, Wuxi, Jiangsu 214122, China;

^2^ Key Laboratory of Industrial Biotechnology, Ministry of Education, School of Biotechnology, Jiangnan University, 1800 Lihu Road, Wuxi, Jiangsu 214122, China;

^3^ Jiangsu Province Engineering Research Center of Food Synthetic Biotechnology, Jiangnan University, 1800 Lihu Road, Wuxi, Jiangsu 214122, China;

^4^ Engineering Research Center of Ministry of Education on Food Synthetic Biotechnology, Jiangnan University, 1800 Lihu Road, Wuxi, Jiangsu 214122, China;

^5^ Key Laboratory of Carbohydrate Chemistry and Biotechnology, Ministry of Education, Jiangnan University, 1800 Lihu Road, Wuxi, Jiangsu 214122, China;

^*^ Corresponding authors: zhaoxinrui@jiangnan.edu.cn

**Table of Contents**

Supplementary Experimental Section

Figures. S1 to S7

Tables S1 to S10

Notes S1 to S9

**Supplementary Experimental Section**

**The heterologous expression of rHb in *E. coil***

The rHb gene controlled by the T7*lac* promoter in pETDuet-1 vector was transformed into *E. coil* BL21(DE3) strain or heme-supply enhanced HEME-R11 strain, and 10 short peptides of his-tag was fused at the C-terminal of rHb for protein purification. The heterologous expression of rHb was successfully achieved using a modified method(Natarajan, et al., 2020). Terrific Broth (TB) medium (1.2% tryptone, 2.4% yeast extract, 0.4% v/v glycerol, 0.2% KH_2_PO_4_ and 1.3% K_2_HPO_4_) supplemented with 50 µg/mL ampicillin was used to synthesize rHb at 37°C (220 rpm). The engineered *E. coli* strains were induced by 0.4 mM IPTG when the value of OD_600_ reached 0.6 and 20 g/L glucose, 50 mg/L sodium hydrosulfite were supplemented to the fermentation broth for rHb synthesis. For the BL21(DE3) strain, 20 mg/L heme was added. Then, the induced strains were grown at 28°C for 16 h (220 rpm).

**Protein purification and quantitation**

The synthesized rHb was suspended into 20.0 mM phosphate buffered saline (PBS, 5.0 mM NaH_2_PO_4_, 48.0 mM Na_2_HPO_4_, 0.5 M NaCl, pH 7.4) and the final concentration of 1 mM phenylmethylsulfonyl fluoride was added as protease inhibitor to prevent the degradation of rHb. The cell wall was broken at 4°C by high pressure homogenizers (Union Biotech, Shanghai, China). The supernatant was collected by centrifugation at 4°C for 10 min (7000 rpm) and was added to His-tag affinity beads (Dianchuang Biotech, Shanghai, China). The 10-fold column volumes of PBS buffer A (pH 7.4, 20.0 mM imidazole in the final concentration) was added to wash the beads. Then, the other proteins were washed out by 10-fold column volume of PBS buffer B (pH 7.4, 80.0 mM imidazole in the final concentration), and rHb was eluted by PBS buffer C (pH 7.4, 0.5 M imidazole). The concentration of imidazole in rHb purification solution was reduced to lower than 50.0 mM by Amicon ®Ultra-0.5 Centrifugal (Merck KGaA, Germany), and the concentration of protein was determined by the Bradford protein Assay Kit (Beyotime Biotech, Shanghai, China). The quality of purified rHb was verified by sodium dodecyl sulfate-polyacrylamide (SDS) gel electrophoresis.

**Measurement of oxygen saturation for recombinant rHb**

Sodium dithionite (SDT) was used as a deoxidizer and reductant for methemoglobin(Correddu, et al., 2023). The rHb and SDT solutions were mixed at the ratio of 1:13 in 96-well black plates (Qianbai Biotech, Shanghai, China) and then rHb was converted to deoxyhemoglobin. Subsequently, the mixture solutions were incubated at 37°C and the values of *A*_556_ and *A*_523_ absorbance were measured through a microplate reader (BioTek synergy H1). The plates were shaken at 300 rpm for 1 min between each round of measurement. The oxygen saturation measure of rHb is defined as the previous method(Okamoto, et al., 2017):

**The measurement of structural stability for rHb through circular dichroism**

CD was performed by a Chirascan spectropolarimeter (Applied Photophysics, JASCO Corporation, Japan), 6.0 M GdnHCl was prepared in 0.2 M potassium phosphate buffer (68.0 mM KH_2_PO_4_, 131.0 mM K_2_HPO_4_, pH 7.0), and the pH value of potassium phosphate buffer was adjusted to 7.0 after adding denaturant GdnHCl. The mixture was prepared including 12.0 µM rHb and different concentrations of GdnHCl (0.5, 1.0, 1.5, 1.7, 1.8, 1.9, 2.0, 2.5, 3.0 and 4.0 M) in 0.2 M potassium phosphate (pH 7.0). The rHb-GdnHCl mixture was incubated in water bath at 10°C for 1 h before any spectral measurement. The CD spectrum of each sample was recorded within the range of 190 to 250 nm by JASCO 1700 CD spectral polarizer (JASCO Corporation, Japan), and the negative ellipticity change at 222 nm was monitored to characterize the change of secondary structure for rHb(Samuel, et al., 2017).

**The measurement of thermal stability for rHb through** **differential scanning calorimetry**

The thermal denaturation process of rHb in the temperature ranges (40-90°C) was studied by nano-DSC (TA Instruments, USA)(Kanagarajan, et al., 2021). The instrument is equipped with a load capacity of 0.3 mL rHb (15.5 µM). The DSC scan of degassed sample (equilibrium sample 600 seconds) was recorded at a constant external pressure of 3 atm at a scanning increase rate of 1°C/min. The rHb_wild-type_ was used as a control, and the Nano analyzer software was used to analyze the data.

**The measurements of rHb autoxidation**

The autoxidation of rHb was carried out by previous method with slight modifications(Yan, et al., 2020). The rHb solution (10.0 µM) was diluted in 0.2 M potassium phosphate (pH 7.0) buffer. Deoxyhemoglobin was prepared by the concentration ratio of 1:13 (rHb solution: SDT solution). The complete spectral recording was carried out in wavelength scanning of 400-700 nm by a microplate reader (BioTek synergy H1) at 37°C for 64 h. The values of oxyhemoglobin and metHb were calculated using a multicomponent analytical method, and the rate of autoxidation in the percentage of oxidized hemoglobin was fitted to a single exponential expression through nonlinear least-squares curve fitting.

**The determination of heme binding rate**

The binding rate of heme was measured by pyridine hemochrome assay with slight modifications(Tupta, et al., 2022). The rHb (0.13 µM) was added to a pyridine solution, including 3.5 mL pyridine and 1.5 mL of 1 M NaOH, then mixed with 0.2 M SDT. The characteristic absorption peak at 410 nm was immediately detected, and the binding rate of heme was characterized by determining (%) mol heme/mol rHb.

**The measurement of heme loss from rHb**

The rate of heme loss from rHb was carried out by previous method(Pires, et al., 2020). Firstly, 6.5 µM metHb and 40.0 µM sperm whale apomyoglobin_H64Y/V68F_ was mixed in 0.1 M PBS buffer with the addition of 0.5 M sucrose (pH 7.4). The sperm whale apomyoglobin_H64Y/V68F_ was served as a high-affinity heme scavenger, and the high concentration of sucrose was used as an osmotic agent to prevent the precipitation of apohemoglobin. The absorbance was recorded in wavelength scanning of 400-650 nm at 37°C for 4 h, and the rate of heme loss was calculated based on the specific absorbance of *A*_410_ through a double exponential expression of nonlinear least-squares curve fitting.

**Molecular dynamics simulation and mechanism analysis related to rHb stability**

MD simulations were performed for 20 ns at 375 K(Kelvins, reaction temperature) through the NAMD Program (http://www.ks.uiuc.edu/Research/namd/). The modeling system was filled with SPC/E water in a cubic box, and the distance was 12 Å between the boundary and rHb. Then, 150 mM Cl^−^ and Na^+^ counter ions were added to ensure electrical neutrality, and molecular interactions were described using CHARMm force field (http://mackerell.umaryland.edu/charmm ff.shtml). The RMSD and RMSF were calculated using the Geo Measures Plugin (https://pymolwiki.org/index.php/Geo_Measures_Plugin). The non-covalent bonds between different mutated sites and the average radius of gyration were analyzed through Discovery Studio 2019 (Accelrys Inc., San Diego, CA, USA).

**Oxygen equilibration curve of rHb**

The oxygen equilibrium curve of rHb was measured using an oxygen binding/releasing instrument (BL00DOX-2018, Beijing, China), and the ferrous hemoglobin was prepared by 0.1 M SDT solution. During the measurement, the partial pressures of air and nitrogen were 0.1 MPa, and the reaction temperature was maintained at 37°C. A mixture of rHb (3.0 mg) and 4.0 mL buffer solution (50.0 mM HEPES, 0.1 M NaCl, 0.1 mM EDTA and 0.1 mM bovine albumin) was injected into the sample pool of oxygen binding/releasing instrument. The air valve was opened to introduce air at a constant rate (~13.0 mL/min) until the value of *P*_O2_ was stable. Subsequently, the instrument was programmed to introduce nitrogen at a constant rate (~13.0 mL/min), to measure the dissociation rate of oxygen until the value of *P*_O2_ was stable. The Hill coefficient of rHb was measured by previous method(Bisse, et al., 2017).

**Supplementary Figures**

**
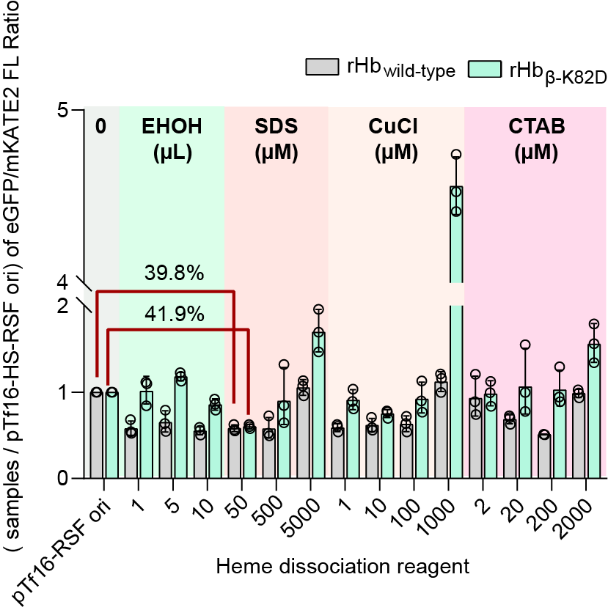
**

**Figure S1.** The heme dissociation reagent accelerated the dissociation of heme from recombinant human hemoglobin (rHb). The gray bands represent rHb_wild-type_, the green bands represent rHb_β-K82D_ with lower rate of autoxidation and heme loss. The numbers (39.8%, 41.9%) above red lines represent the decreased fold of eGFP/mKATE2 relative to rHb treated with 50 µM SDS compared to the control pTf16-RSF *ori* (without heme dissociation reagent). Four heme dissociation reagents, EHOH: acid alcohol, SDS: sodium dodecyl sulfate, CuCl: Cuprous chloride, CTAB: Cetyltrimethylammonium Bromide. Statistical evaluation was performed through a two-tailed t-test (*p* value).

**
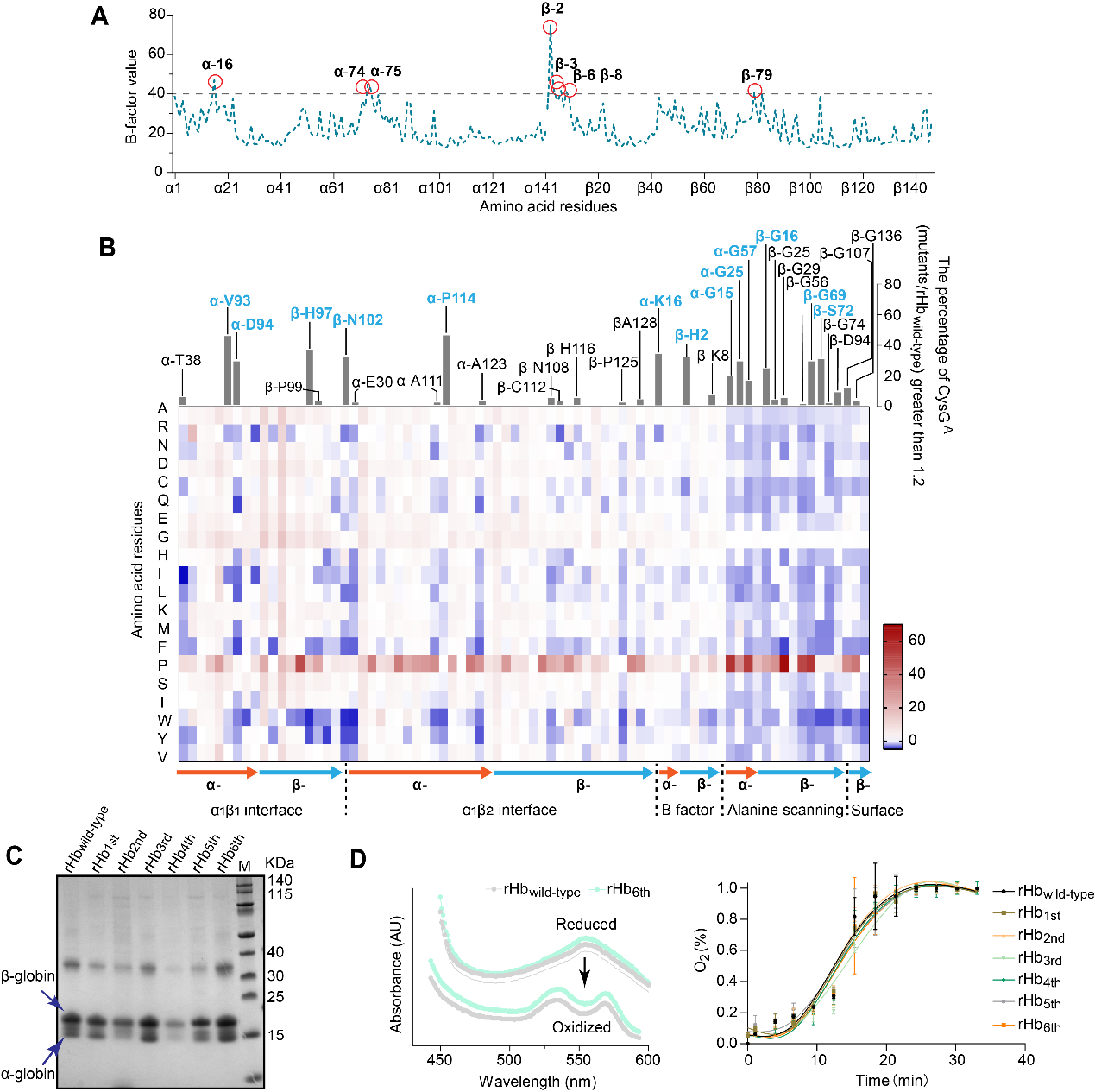
**

**Figure S2.** Identification of mutation sites and verification of properties for stability modification of rHb. (A) Identification of sites that affect hemoglobin stability based on the B-factor value of amino acids. (B) Virtual saturation mutagenesis of sites for stability modification of rHb, and hot residues were selected to conduct iterative saturation mutagenesis based on the ratio of CysG^A^ fluorescent intensity between the mutants (> 1.2) and control (rHb_wild-type_). Among these residues, blue font represents the sites used to enhance the stability of rHb. (C) SDS-PAGE analysis of expressed rHb mutants. (D) Oxygen affinity curves of rHb mutants.

**
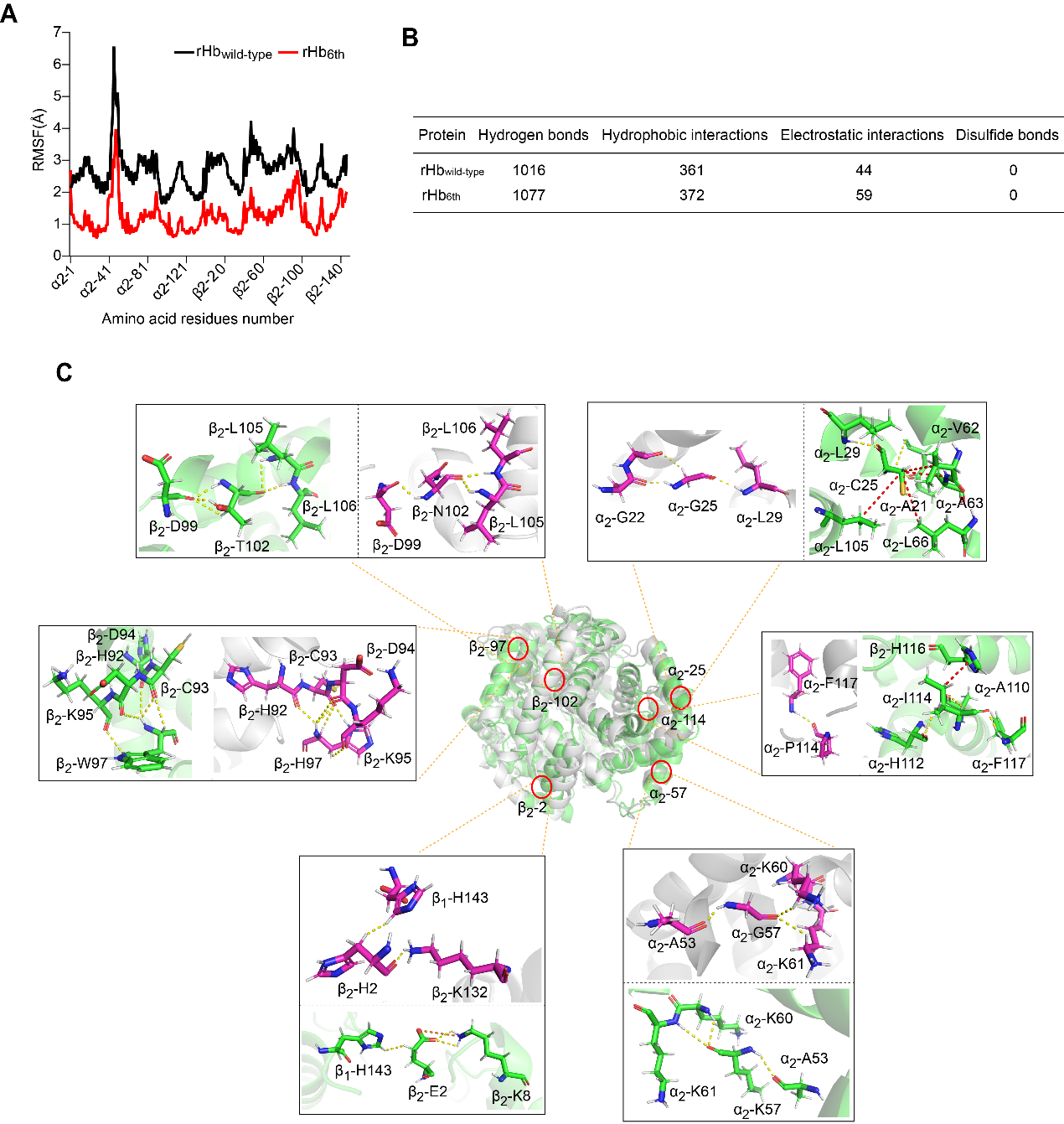
**

**Figure S3.** The stability mechanism of rHb. (A) RMSF results in α2β2 dimer of rHb_wild-type_ and rHb_6th_. (B) Changes of covalent bonds between rHb_wild-type_ and rHb_6th_. (C) The non-covalent bonds formed among the mutated residues in α_2_β_2_ dimer of rHb_6th_ and rHb_wild-type_ (PDB ID: 2DN2). Yellow dotted line: hydrogen bonding interaction, red dotted line: hydrophobic interaction, orange dotted line: electrostatic force.

**
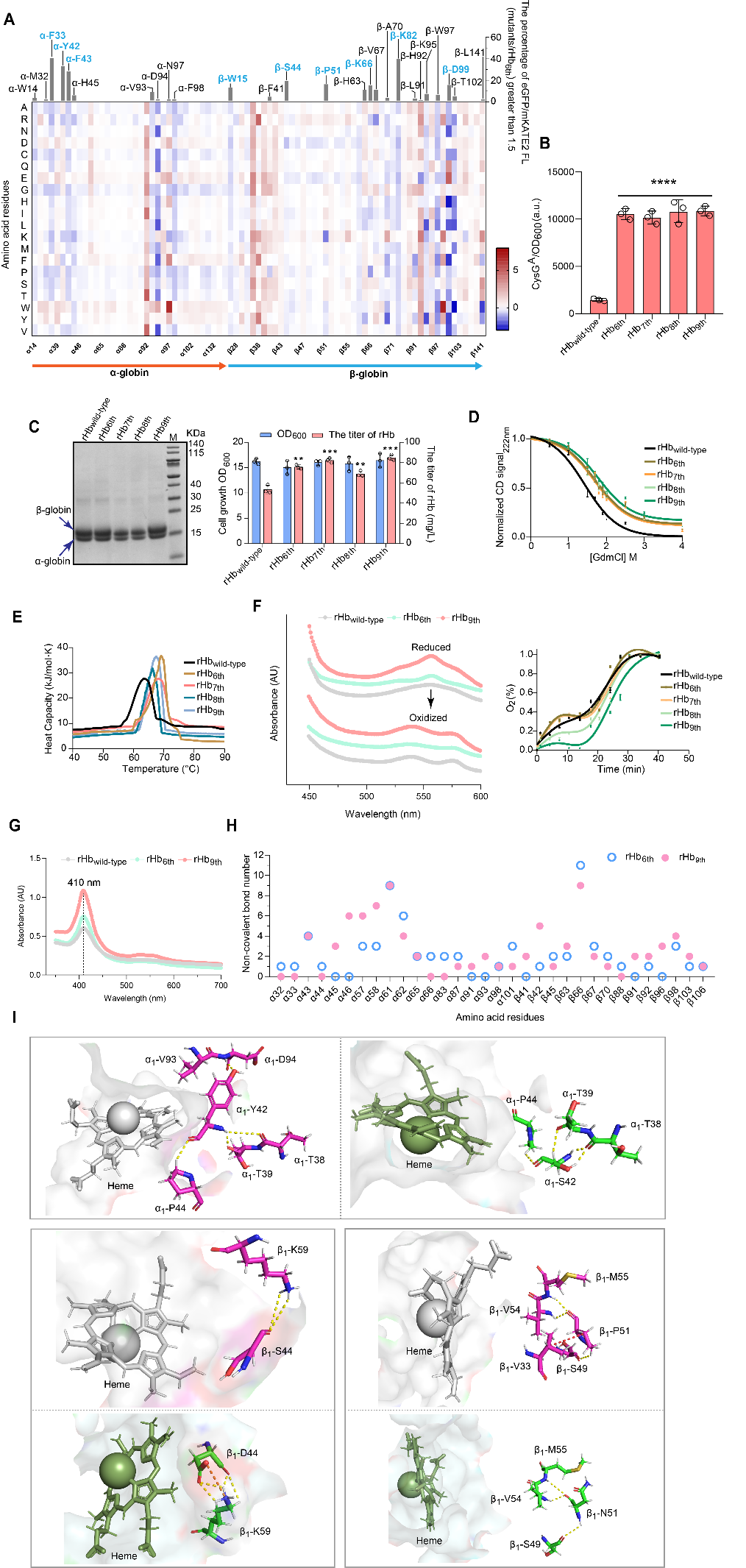
**

**Figure S4.** Identification of mutation sites and verification of properties for heme-binding enhanced of rHb mutants. (A) Virtual saturation mutagenesis of sites for stability modification of rHb, and hot sites were selected for iterative saturation mutagenesis based on the fluorescent ratio of eGFP/mKATE2 between the mutants (> 1.50-fold) and control (rHb_6th_). Among these residues, blue font represents the sites used to screen heme-binding enhanced mutants. (B) Compare with the fluorescent values of rHb_wild-type_, the fluorescent values of CysG^A^ for rHb mutants (rHb_6th_, rHb_7th_, rHb_8th_ and rHb_9th_). (C–F) The properties of ideal mutants determined by each round of iterative saturation mutagenesis: (C) The titers of rHb and SDS-PAGE analysis, (D) secondary structure of mutants determined by CD spectroscopy, (E) thermal stability of mutants determined by DSC data, (F) oxygen affinity curves. (G) Full-wavelength scan (350-700 nm) results of rHb mutants. (H) The numbers of non-covalent interactions related to heme in rHb_6th_ and rHb_9th_. (I) Compared with rHb_6th_, the formed non-covalent bonds at the mutated residues at α1β1 dimer of rHb_9th_. Yellow dotted line: hydrogen bonding interaction, red dotted line: hydrophobic interaction. Data was expressed as mean value ± SD from three independent biological replicates (n = 3). Statistical evaluation was performed through a two-tailed t-test, compared to the control rHb_wild-type_ (*p* value). ***p* < 0.01, ****p* < 0.001.

**
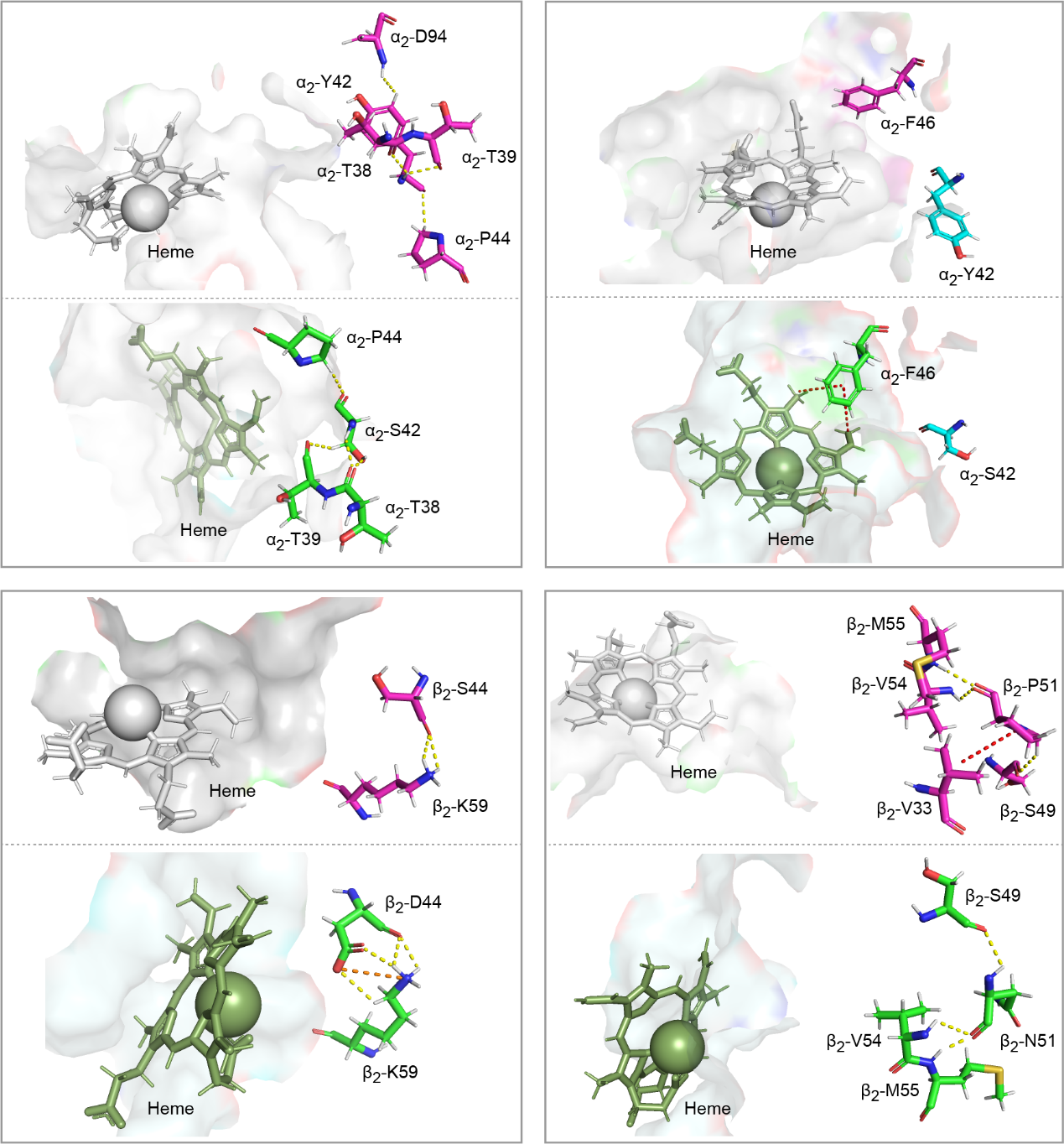
**

**Figure S5.** Compared with rHb_6th_, the non-covalent bonds formed at the mutated residues in α_2_β_2_ dimer of rHb_9th_ (PDB ID: 2DN2). Yellow dotted line: hydrogen bonding interaction, red dotted line: hydrophobic interaction.

**
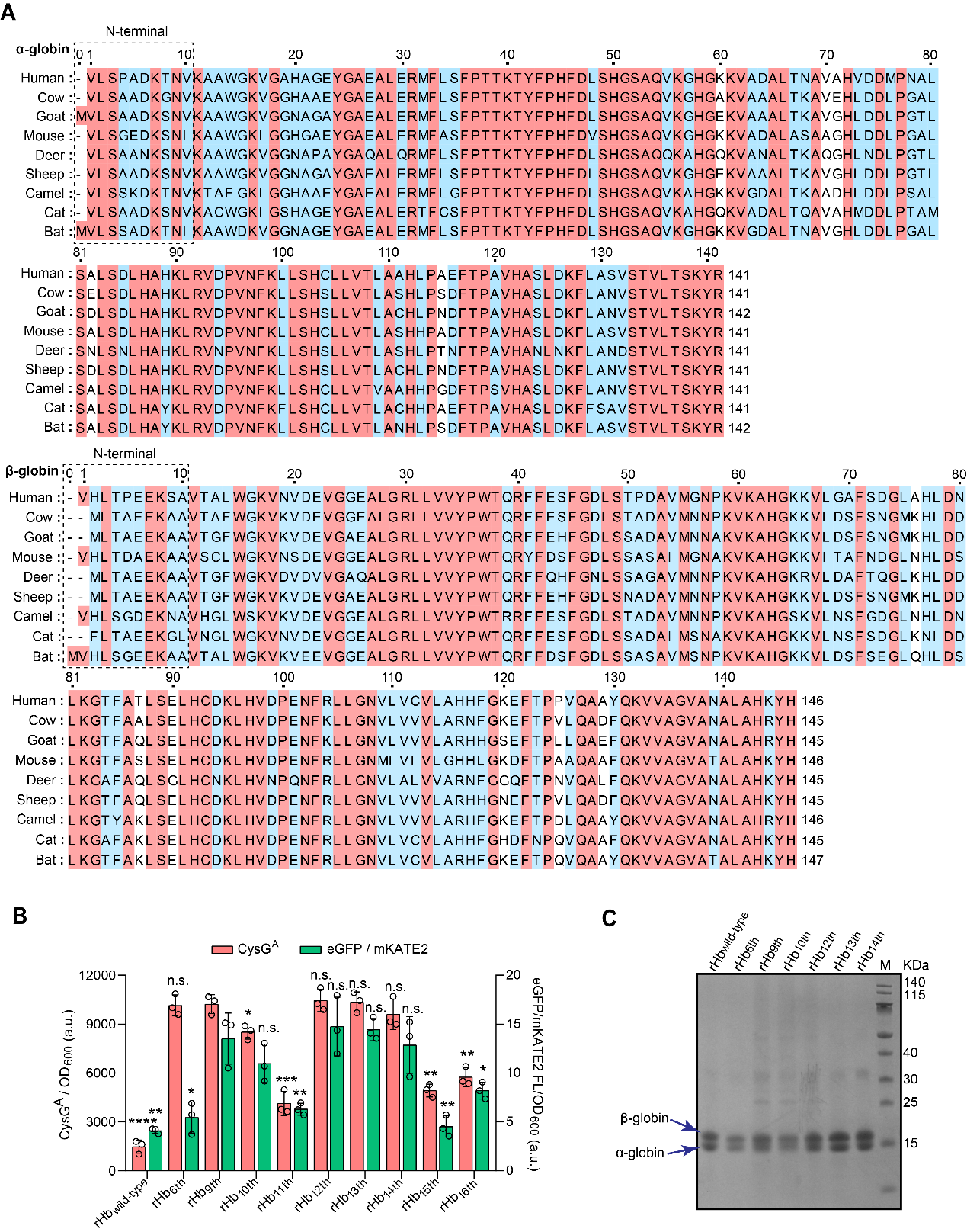
**

**Figure S6.** Design and screen of rHb mutants with enhanced oxygen-carrying capacity. (A) Homology alignment of sequences between rHb_wild-type_ and eight hemoglobins with lower oxygen affinities. The red and blue background indicates the conserved residues and partially conserved residues, respectively. (B) The stability of rHb mutants with enhanced oxygen-carrying capacity were verified through fluorescent intensity of CysG^A^ and the fluorescent ratio of eGFP/mKATE2. (C) SDS-PAGE analysis of expressed rHb. Data was presented as mean value ± SD from three independent biological replicates (n = 3). Statistical evaluation was performed through a two-tailed t-test, compared to the control rHb_9th_ (*p*-value). **p* < 0.05, ***p* < 0.01, ****p* < 0.001, *****p* < 0.0001, n. s. represents no statistical significance (*p* ≥ 0.05).

**
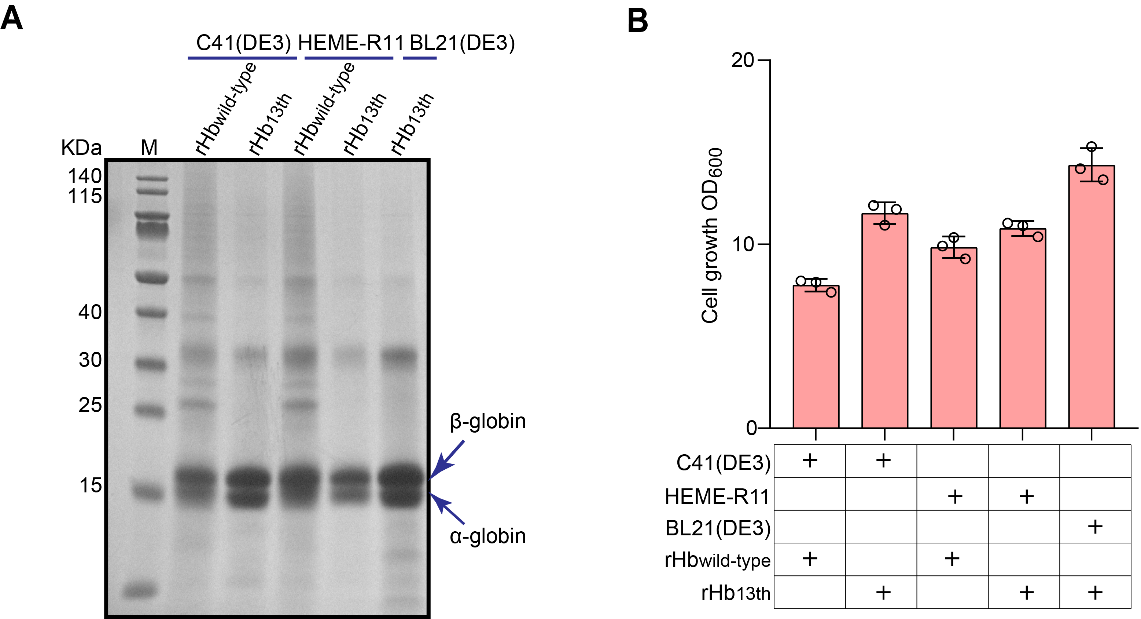
**

**Figure S7.** Expression of rHb in heme-supply enhanced *E. coli* strains. (A) SDS-PAGE analysis of rHb_wild-type_ and rHb_13th_ expressed. (B) Biomass of rHb_wild-type_ and rHb_13th_. Data was expressed as mean value ± SD from three independent biological replicates (n = 3).

**Supplementary Tables:**

Table S1. The plasmids used in the study. Abbreviations: Ap, ampicillin; Km, kanamycin; Cm, chloramphenicol; R, resistance

| Plasmids | Description | Source |
| --- | --- | --- |
| pUC57 | Cloning vector, pUC *ori*, Ap^R^; *E. coli* | Lab stock |
| pETDuet-1 | Expression vector, ColE1 *ori*, double T7 *lac* promoters, Ap^R^; *E. coli* | Lab stock |
| pTf16 | Expression vector, p15A *ori*, araB promoters, L-Arabinose induction, Cm^R^; *E. coli* | Lab stock |
| pMAL-c5X | Expression vector, pUC *ori*, tac *lac* promoters, MBP label, Ap^R^; *E. coli* | Lab stock |
| pRSFDuet-1 | Expression vector, RSF1030 *ori*, double T7 *lac* promoters, Km^R^; *E. coli* | Lab stock |
| pACYCDuet-1 | Expression vector, p15A *ori*, double T7 *lac* promoters, Cm^R^; *E. coli* | Lab stock |
| pET-rHb_wild-type_ | pETDuet-1derivative, human adult hemoglobin α-globin and β-globin,10 X His tag, Ap^R^ | This study |
| pET-rHb_wild-type_-MAP | pET-rHb_wild_ derivative, methionine aminopeptidase, Ap^R^ | This study |
| pET-rHb_wild-type_-CysG^A^_G4S_ | pET-rHb_wild-type_ derivative, CysG^A^, G_4_S  Linker for β-globin, Ap^R^ | This study |
| pET-rHb_wild-type_-CysG^A^_(G4S)2_ | pET-rHb_wild-type_ derivative, CysG^A^, (G_4_S)_2_  Linker for β-globin, Ap^R^ | This study |
| pET-rHb_wild-type_-CysG^A^_(G4S)3_ | pET-rHb_wild-type_ derivative, CysG^A^, (G_4_S)_3_  Linker for β-globin, Ap^R^ | This study |
| pET-rHb_wild-type_-CysG^A^_(S2G)2_ | pET-rHb_wild-type_ derivative, CysG^A^, (S_2_G)_2_  Linker for β-globin, Ap^R^ | This study |
| pET-rHb_wild-type_-CysG^A^_(G2S4)9_ | pET-rHb_wild-type_ derivative, CysG^A^, (G_2_S_4_)_9_  Linker for β-globin, Ap^R^ | This study |
| pET-rHb_wild-type_-CysG^A^_GGS10_ | pET-rHb_wild-type_ derivative, CysG^A^, GGS_10_  Linker for β-globin, Ap^R^ | This study |
| pET-CysG^A^_(S2G)2_- rHb_α-P119S_ | pET-rHb_wild-type_-CysG^A^_(G4S)2_ derivative,  rHb_α-P119S_, Ap^R^ | This study |
| pET-CysG^A^_(S2G)2_- rHb_α-L29W_ | pET-rHb_wild-type_-CysG^A^_(G4S)2_ derivative,  rHb_α-L29W_, Ap^R^ | This study |
| pET-CysG^A^_(S2G)2_- rHb_β-L28W_ | pET-rHb_wild-type_-CysG^A^_(G4S)2_ derivative,  rHb_β-L28W_, Ap^R^ | This study |
| pET-CysG^A^_(S2G)2_- rHb_α-G15A_ | pET-rHb_wild-type_-CysG^A^_(G4S)2_ derivative,  rHb_α-G15A_, Ap^R^ | This study |
| pET-CysG^A^_(S2G)2_- rHb_α-G15A/β-G16A/β-H116I_ | pET-rHb_wild-type_-CysG^A^_(G4S)2_ derivative,  rHb_α-G15A/β-G16A/β-H116I_, Ap^R^ | This study |
| pET-CysG^A^_(S2G)2_- rHb_α-G15A/β-G16A_ | pET-rHb_wild-type_-CysG^A^_(G4S)2_ derivative,  rHb_α-G15A/β-G16A_, Ap^R^ | This study |
| pET-CysG^A^_(S2G)3_- rHb_α-P119S_ | pET-rHb_wild-type_-CysG^A^_(G4S)3_ derivative,  rHb_α-P119S_, Ap^R^ | This study |
| pET-CysG^A^_(S2G)3_- rHb_α-L29W_ | pET-rHb_wild-type_-CysG^A^_(G4S)3_ derivative,  rHb_α-L29W_, Ap^R^ | This study |
| pET-CysG^A^_(S2G)3_- rHb_β-L28W_ | pET-rHb_wild-type_-CysG^A^_(G4S)3_ derivative,  rHb_β-L28W_, Ap^R^ | This study |
| pET-CysG^A^_(S2G)3_- rHb_α-G15A_ | pET-rHb_wild-type_-CysG^A^_(G4S)3_ derivative,  rHb_α-G15A_, Ap^R^ | This study |
| pET-CysG^A^_(S2G)3_- rHb_α-G15A/β-G16A/β-H116I_ | pET-rHb_wild-type_-CysG^A^_(G4S)3_ derivative,  rHb_α-G15A/β-G16A/β-H116I_, Ap^R^ | This study |
| pET-CysG^A^_(S2G)3_- rHb_α-G15A/β-G16A_ | pET-rHb_wild-type_-CysG^A^_(G4S)3_ derivative,  rHb_α-G15A/β-G16A_, Ap^R^ | This study |
| pTf16-HS_M7A_ | pTf16 derivative, HS_M7A,_ Cm^R^ | This study |
| pTf16-HS_M7A_-RSF | pTf16-HS_M7A_ derivative, p15A *ori* was  replaced to RSF *ori*, Cm^R^ | This study |
| pET-rHb_β-F41Y_ | pET-rHb_wild-type_-MAP derivative, rHb_β-F41Y_, Ap^R^ | This study |
| pET-rHb_β-T84Y_ | pET-rHb_wild-type_-MAP derivative, rHb_β-T84Y_, Ap^R^ | This study |
| pET-rHb_β-H92Q_ | pET-rHb_wild-type_-MAP derivative, rHb_β-H92Q_, Ap^R^ | This study |
| pET-rHb_β-S44H_ | pET-rHb_wild-type_-MAP derivative, rHb_β-S44H_, Ap^R^ | This study |
| pET-rHb_β-V67T_ | pET-rHb_wild-type_-MAP derivative, rHb_β-V67T_, Ap^R^ | This study |
| pET-rHb_α-H58L_ | pET-rHb_wild-type_-MAP derivative, rHb_α-H58L_, Ap^R^ | This study |
| pET-rHb_β-K82D_ | pET-rHb_wild-typed_-MAP derivative, rHb_β-K82D_, Ap^R^ | This study |
| pET-CysG^A^-  rHb_α-G15_ | pET-rHb_wild-type_-CysG^A^_(G4S)3_ derivative,  rHb_α-G15_ saturation mutation, Ap^R^ | This study |
| pET-CysG^A^-  rHb_α-K16_ | pET-rHb_wild-type_-CysG^A^_(G4S)3_ derivative,  rHb_α-K16_ saturation mutation, Ap^R^ | This study |
| pET-CysG^A^-  rHb_α-G25_ | pET-rHb_wild-type_-CysG^A^_(G4S)3_ derivative,  rHb_α-G25_ saturation mutation, Ap^R^ | This study |
| pET-CysG^A^-  rHb_α-G57_ | pET-rHb_wild-type_-CysG^A^_(G4S)3_ derivative,  rHb_α-G57_ saturation mutation, Ap^R^ | This study |
| pET-CysG^A^-  rHb_α-V93_ | pET-rHb_wild-type_-CysG^A^_(G4S)3_ derivative,  rHb_α-V93_ saturation mutation, Ap^R^ | This study |
| pET-CysG^A^-  rHb_α-D94_ | pET-rHb_wild-type_-CysG^A^_(G4S)3_ derivative,  rHb_α-D94_ saturation mutation, Ap^R^ | This study |
| pET-CysG^A^-  rHb_α-P114_ | pET-rHb_wild-type_-CysG^A^_(G4S)3_ derivative,  rHb_α-P114_ saturation mutation, Ap^R^ | This study |
| pET-CysG^A^-rHb_β-H2_ | pET-rHb_wild-type_-CysG^A^_(G4S)3_ derivative,  rHb_β-H2_ saturation mutation, Ap^R^ | This study |
| pET-CysG^A^-  rHb_β-G16_ | pET-rHb_wild-type_-CysG^A^_(G4S)3_ derivative,  rHb_β-G16_ saturation mutation, Ap^R^ | This study |
| pET-CysG^A^-  rHb_β-G69_ | pET-rHb_wild-type_-CysG^A^_(G4S)3_ derivative,  rHb_β-G69_ saturation mutation, Ap^R^ | This study |
| pET-CysG^A^-  rHb_β-S72_ | pET-rHb_wild-type_-CysG^A^_(G4S)3_ derivative,  rHb_β-S72_ saturation mutation, Ap^R^ | This study |
| pET-CysG^A^-  rHb_β-H97_ | pET-rHb_wild-type_-CysG^A^_(G4S)3_ derivative,  rHb_β-H97_ saturation mutation, Ap^R^ | This study |
| pET-CysG^A^-  rHb_β-N102_ | pET-rHb_wild-type_-CysG^A^_(G4S)3_ derivative,  rHb_β-N102_ saturation mutation, Ap^R^ | This study |
| pET-rHb_1st_-CysG^A^ | pET-rHb_wild-type_-CysG^A^_(G4S)3_ derivative, rHb_α-P114I_, Ap^R^ | This study |
| pET-rHb_2nd_-CysG^A^ | pET-rHb_1st_-CysG^A^ derivative, rHb_1st/α-G25C_, Ap^R^ | This study |
| pET-rHb_3rd_-CysG^A^ | pET-rHb_2nd_-CysG^A^ derivative, rHb_2nd/β-H2E_, Ap^R^ | This study |
| pET-rHb_4th_-CysG^A^ | pET-rHb_3rd_-CysG^A^ derivative, rHb_3rd/α-G57K_, Ap^R^ | This study |
| pET-rHb_5th_-CysG^A^ | pET-rHb_4th_-CysG^A^ derivative, rHb_4th/β-N102T_, Ap^R^ | This study |
| pET-rHb_6th_-CysG^A^ | pET-rHb_5th_-CysG^A^ derivative, rHb_5th/β-H97W_, Ap^R^ | This study |
| pET-rHb_1st_ | pET-rHb_wild-type_-MAP derivative, rHb_α-P114I_, Ap^R^ | This study |
| pET-rHb_2nd_ | pET-rHb_1st_ derivative, rHb_1st/α-G25C_, Ap^R^ | This study |
| pET-rHb_3rd_ | pET-rHb_2nd_ derivative, rHb_2nd/β-H2E_, Ap^R^ | This study |
| pET-rHb_4th_ | pET-rHb_3rd_ derivative, rHb_3rd/α-G57K_, Ap^R^ | This study |
| pET-rHb_5th_ | pET-rHb_4th_ derivative, rHb_4th/β-N102T_, Ap^R^ | This study |
| pET-rHb_6th_ | pET-rHb_5th_ derivative, rHb_5th/β-H97W_, Ap^R^ | This study |
| pACYC-sRNA_HemB_-HrtR_H149K_ | pACYC-R1 derivative, *eGFP* was replaced with *sRNA-HemB-micC*, HrtR_H149K_, Cm^R^ | (Hu, et al., 2023) |
| pET-rHb_α-F33_ | pET-rHb_6th_ derivative, rHb_α-F33_ saturation mutation, Ap^R^ | This study |
| pET-rHb_α-Y42_ | pET-rHb_6th_ derivative, rHb_α-Y42_ saturation mutation, Ap^R^ | This study |
| pET-rHb_α-F43_ | pET-rHb_6th_ derivative, rHb_α-F43_ saturation mutation, Ap^R^ | This study |
| pET-rHb_β-W15_ | pET-rHb_6th_ derivative, rHb_β-W15_ saturation mutation, Ap^R^ | This study |
| pET-rHb_β-S44_ | pET-rHb_6th_ derivative, rHb_β-S44_ saturation mutation, Ap^R^ | This study |
| pET-rHb_β-P51_ | pET-rHb_6th_ derivative, rHb_β-P51_ saturation mutation, Ap^R^ | This study |
| pET-rHb_β-K66_ | pET-rHb_6th_ derivative, rHb_β-K66_ saturation mutation, Ap^R^ | This study |
| pET-rHb_β-K82_ | pET-rHb_6th_ derivative, rHb_β-K82_ saturation mutation, Ap^R^ | This study |
| pET-rHb_β-D99_ | pET-rHb_6th_ derivative, rHb_β-D99_ saturation mutation, Ap^R^ | This study |
| pET-rHb_7th_ | pET-rHb_6th_ derivative, rHb_6th /α-Y42S_, Ap^R^ | This study |
| pET-rHb_8th_ | pET-rHb_7th_ derivative, rHb_7th/β-P51N_, Ap^R^ | This study |
| pET-rHb_9th_ | pET-rHb_8th_ derivative, rHb_8th/β-S44D_, Ap^R^ | This study |
| pET-rHb_7th_-CysG^A^ | pET-rHb_6th_-CysG^A^ derivative, rHb_6th/α-Y42S_, Ap^R^ | This study |
| pET-rHb_8th_-CysG^A^ | pET-rHb_7th_-CysG^A^ derivative, rHb_7th/β-P51N_, Ap^R^ | This study |
| pET-rHb_9th_-CysG^A^ | pET-rHb_8th_-CysG^A^ derivative, rHb_8th/β-S44D_, Ap^R^ | This study |
| pET-sMb | pETDuet-1derivative, sperm whale apomyoglobin_H64Y/V68F_,10 X His tag, Ap^R^ | This study |
| pET-rHb_10th_ | pET-rHb_9th_ derivative, rHb_9th/α-K60G/A71G_, Ap^R^ | This study |
| pET-rHb_11th_ | pET-rHb_9th_ derivative, rHb_9th/β-V1M/E2deleted/T4I/P5A/A76K_, Ap^R^ | This study |
| pET-rHb_12th_ | pET-rHb_9th_ derivative, rHb_9th/β-V1M/T4I/P5A/A76K_, Ap^R^ | This study |
| pET-rHb_13th_ | pET-rHb_9th_ derivative, rHb_9th/(α-V1E, β-N108K)_, Ap^R^ | This study |
| pET-rHb_14th_ | pET-rHb_9th_ derivative, rHb_9th/β-T4S/P5G/A76K_, Ap^R^ | This study |
| pET-rHb_15th_ | pET-rHb_9th_ derivative, rHb_9th/β-0Minserted/T4S/P5G/A76K_, Ap^R^ | This study |
| pET-rHb_16th_ | pET-rHb_9th_ derivative, rHb_9th/β-H116R_, Ap^R^ | This study |
| pET-rHb_10th_-CysG^A^ | pET-rHb_9th_-CysG^A^ derivative, rHb_9th/α-K60G/A71G_, Ap^R^ | This study |
| pET-rHb_11th_-CysG^A^ | pET-rHb_9th_-CysG^A^ derivative, rHb_9th/β-V1M/E2deleted/T4I/P5A/A76K_, Ap^R^ | This study |
| pET-rHb_12th_-CysG^A^ | pET-rHb_9th_-CysG^A^ derivative, rHb_9th/β-V1M/T4I/P5A/A76K_, Ap^R^ | This study |
| pET-rHb_13th_-CysG^A^ | pET-rHb_9th_-CysG^A^ derivative, rHb_9th/(α-V1E, β-N108K)_, Ap^R^ | This study |
| pET-rHb_14th_- CysG^A^ | pET-rHb_9th_-CysG^A^ derivative, rHb_9th/β-T4S/P5G/A76K_, Ap^R^ | This study |
| pET-rHb_15th_-CysG^A^ | pET-rHb_9th_-CysG^A^ derivative, rHb_9th/β-0Minserted/T4S/P5G/A76K_, Ap^R^ | This study |
| pET-rHb_16th_-CysG^A^ | pET-rHb_9th_-CysG^A^ derivative, rHb_9th/β-H116R_, Ap^R^ | This study |
| pRSF-rHb_wild-type_-MAP | pRSFDuet-1 derivative, methionine aminopeptidase, human adult hemoglobin α-globin and β-globin, 10 X His tag, Km^R^ | This study |
| pRSF-rHb_13th_-MAP | pRSFDuet-1 derivative, methionine aminopeptidase, rHb_13th_, 10 X His tag, Km^R^ | This study |

Table S2. The strains used in the study

| Strains | Description | Source |
| --- | --- | --- |
| DH5α | *E. coli* str. K-12 F^–^ *endA1* *glnV44 thi-1 recA1 relA1 gyrA96 deoR nupG purB20* φ80d*lacZ*ΔM15 Δ(*lacZYA*-*argF*) U169 *hsdR17*(*_rK_*^–^*_mK_*^+^) λ^–^ | Lab stock |
| BL21(DE3) | *E. coli str*. B F^–^ *ompT gal dcm lon hsdS_B_*(_rB_^–^_mB_^–^) λ (DE3 [*lacI lacUV5-T7p07 ind1 sam7 nin5*]) [*malB*^+^] _K-12_(λ^S^) | Lab stock |
| C41(DE3) | Derived from BL21(DE3) by selecting for resistance to OGCP overexpression | Lab stock |
| BL21-pET-rHb_wild-type_ | BL21(DE3) harboring plasmid pET-rHb_wild-type_, Ap^R^ | This study |
| BL21-pET-rHb_wild-type_-MAP | BL21(DE3) harboring plasmid pET-rHb_wild-type_-MAP, Ap^R^ | This study |
| BL21-pET- CysG^A^_G4S_ | BL21(DE3) harboring plasmid pET-rHb_wild-type_-CysG^A^_G4S_, Ap^R^ | This study |
| BL21-pET- CysG^A^_(G4S)2_ | BL21(DE3) harboring plasmid pET-rHb_wild-type_-CysG^A^_(G4S)2_, Ap^R^ | This study |
| BL21-pET- CysG^A^_(G4S)3_ | BL21(DE3) harboring plasmid pET-rHb_wild-type_-CysG^A^_(G4S)3_, Ap^R^ | This study |
| BL21-pET- CysG^A^_(S2G)2_ | BL21(DE3) harboring plasmid pET-rHb_wild-type_-CysG^A^_(S2G)2_, Ap^R^ | This study |
| BL21-pET- CysG^A^_(G2S4)9_ | BL21(DE3) harboring plasmid pET-rHb_wild-type_-CysG^A^_(G2S4)9_, Ap^R^ | This study |
| BL21-pET- CysG^A^_GGS10_ | BL21(DE3) harboring plasmid pET-rHb_wild-type_-  CysG^A^_GGS10_, Ap^R^ | This study |
| BL21-pET- CysG^A^_(S2G)2_-  rHb_α-G15A_ | BL21(DE3) harboring plasmid pET-CysG^A^_(S2G)2_-  rHb_α-G15A_, Ap^R^ | This study |
| BL21-pET- CysG^A^_(S2G)2_-rHb_α-G15A/β-G16A/β-H116I_ | BL21(DE3) harboring plasmid pET-CysG^A^_(S2G)2_-  rHb_α-G15A/β-G16A/β-H116I_, Ap^R^ | This study |
| BL21-pET- CysG^A^_(S2G)2_-  rHb_α-G15A/β-G16A_ | BL21(DE3) harboring plasmid pET-CysG^A^_(S2G)2_-  rHb_α-G15A/β-G16A_, Ap^R^ | This study |
| BL21-pET- CysG^A^_(S2G)2_-  rHb_α-P119S_ | BL21(DE3) harboring plasmid pET-CysG^A^_(S2G)2_-  rHb_α-P119S_, Ap^R^ | This study |
| BL21-pET- CysG^A^_(S2G)2_-  rHb_α-L29W_ | BL21(DE3) harboring plasmid pET-CysG^A^_(S2G)2_-  rHb_α-L29W_, Ap^R^ | This study |
| BL21-pET- CysG^A^_(S2G)2_-  rHb_β-L28W_ | BL21(DE3) harboring plasmid pET-CysG^A^_(S2G)2_-  rHb_β-L28W_, Ap^R^ | This study |
| BL21-pET- CysG^A^_(S2G)3_-  rHb_α-G15A_ | BL21(DE3) harboring plasmid pET-CysG^A^_(S2G)3_-  rHb_α-G15A_, Ap^R^ | This study |
| BL21-pET- CysG^A^_(S2G)3_-rHb_α-G15A/β-G16A/β-H116I_ | BL21(DE3) harboring plasmid pET-CysG^A^_(S2G)3_-  rHb_α-G15A/β-G16A/β-H116I_, Ap^R^ | This study |
| BL21-pET- CysG^A^_(S2G)3_-  rHb_α-G15A/β-G16A_ | BL21(DE3) harboring plasmid pET-CysG^A^_(S2G)3_-  rHb_α-G15A/β-G16A_, Ap^R^ | This study |
| BL21-pET- CysG^A^_(S2G)3_-  rHb_α-P119S_ | BL21(DE3) harboring plasmid pET-CysG^A^_(S2G)3_-  rHb_α-P119S_, Ap^R^ | This study |
| BL21-pET- CysG^A^_(S2G)3_-  rHb_α-L29W_ | BL21(DE3) harboring plasmid pET-CysG^A^_(S2G)3_-  rHb_α-L29W_, Ap^R^ | This study |
| BL21-pET- CysG^A^_(S2G)3_-  rHb_β-L28W_ | BL21(DE3) harboring plasmid pET-CysG^A^_(S2G)3_-  rHb_β-L28W_, Ap^R^ | This study |
| HEME-R11 | *hemA^fbr^-hemL-scaffold1-scaffold1-scaffold3-scaffold2* integrated into the *cheY* locus and the fragment of pT7-*hemH*-pT7-*ADB1-hemB-ADB3-hemD-ADB2-hemC* multicopy chromosomal integrated in C41(DE3) (9 copies), harboring plasmid pACYC-sRNA_HemB_-HrtR_H149K_, Cm^R^ | (Hu, et al., 2023) |
| R11-pTf16-HS_M7A_ | HEME-R11 harboring plasmid pTf16-HS_M7A_, Cm^R^ | This study |
| R11-pTf16- HS_M7A_-RSF | HEME-R11 harboring plasmid pTf16- HS_M7A_-RSF, Cm^R^ | This study |
| R11-pET-  rHb_β-F41Y_ | R11-pTf16-HS_M7A_-RSF harboring plasmid  pET-rHb_β-F41Y_, Cm^R^, Ap^R^ | This study |
| R11-pET-  rHb_β-T84Y_ | R11-pTf16-HS_M7A_-RSF harboring plasmid  pET-rHb_β-T84Y_, Cm^R^, Ap^R^ | This study |
| R11-pET-  rHb_β-H92Q_ | R11-pTf16-HS_M7A_-RSF harboring plasmid  pET-rHb_β-H92Q_, Cm^R^, Ap^R^ | This study |
| R11-pET-  rHb_β-S44H_ | R11-pTf16-HS_M7A_-RSF harboring plasmid  pET-rHb_β-S44H_, Cm^R^, Ap^R^ | This study |
| R11-pET-  rHb_β-V67T_ | R11-pTf16-HS_M7A_-RSF harboring plasmid  pET-rHb_β-V67T_, Cm^R^, Ap^R^ | This study |
| R11-pET-  rHb_α-H58L_ | R11-pTf16-HS_M7A_-RSF harboring plasmid  pET-rHb_α-H58L_, Cm^R^, Ap^R^ | This study |
| R11-pET-  rHb_β-K82D_ | R11-pTf16-HS_M7A_-RSF harboring plasmid  pET-rHb_β-K82D_, Cm^R^, Ap^R^ | This study |
| BL21-pET-CysG^A^-rHb_α-G15_ | BL21(DE3) harboring plasmid  pET-CysG^A^-rHb_α-G15_, Ap^R^ | This study |
| BL21-pET-CysG^A^-rHb_α-K16_ | BL21(DE3) harboring plasmid  pET-CysG^A^-rHb_α-K16_, Ap^R^ | This study |
| BL21-pET-CysG^A^-rHb_α-G25_ | BL21(DE3) harboring plasmid  pET-CysG^A^-rHb_α-G25_, Ap^R^ | This study |
| BL21-pET-CysG^A^-rHb_α-G57_ | BL21(DE3) harboring plasmid  pET-CysG^A^-rHb_α-G57_, Ap^R^ | This study |
| BL21-pET-CysG^A^-rHb_α-V93_ | BL21(DE3) harboring plasmid  pET-CysG^A^-rHb_α-V93_, Ap^R^ | This study |
| BL21-pET-CysG^A^-rHb_α-D94_ | BL21(DE3) harboring plasmid  pET-CysG^A^-rHb_α-D94_, Ap^R^ | This study |
| BL21-pET-CysG^A^-rHb_α-P114_ | BL21(DE3) harboring plasmid  pET-CysG^A^-rHb_α-P114_, Ap^R^ | This study |
| BL21-pET-CysG^A^-rHb_β-H2_ | BL21(DE3) harboring plasmid  pET-CysG^A^-rHb_β-H2_, Ap^R^ | This study |
| BL21-pET-CysG^A^-rHb_β-G16_ | BL21(DE3) harboring plasmid  pET-CysG^A^-rHb_β-G16_, Ap^R^ | This study |
| BL21-pET-CysG^A^-rHb_β-G69_ | BL21(DE3) harboring plasmid  pET-CysG^A^-rHb_β-G69_, Ap^R^ | This study |
| BL21-pET-CysG^A^-rHb_β-S72_ | BL21(DE3) harboring plasmid  pET-CysG^A^-rHb_β-S72_, Ap^R^ | This study |
| BL21-pET-CysG^A^-rHb_β-H97_ | BL21(DE3) harboring plasmid  pET-CysG^A^-rHb_β-H97_, Ap^R^ | This study |
| BL21-pET-CysG^A^-rHb_β-N102_ | BL21(DE3) harboring plasmid  pET-CysG^A^-rHb_β-N102_, Ap^R^ | This study |
| BL21-pET-rHb_1st_- CysG^A^ | BL21(DE3) harboring plasmid  pET-rHb_1st_-CysG^A^, Ap^R^ | This study |
| BL21-pET-rHb_2nd_- CysG^A^ | BL21(DE3) harboring plasmid  pET-rHb_2nd_-CysG^A^, Ap^R^ | This study |
| BL21-pET-rHb_3rd_- CysG^A^ | BL21(DE3) harboring plasmid  pET-rHb_3rd_-CysG^A^, Ap^R^ | This study |
| BL21-pET-rHb_4th_- CysG^A^ | BL21(DE3) harboring plasmid  pET-rHb_4th_-CysG^A^, Ap^R^ | This study |
| BL21-pET-rHb_5th_- CysG^A^ | BL21(DE3) harboring plasmid  pET-rHb_5th_-CysG^A^, Ap^R^ | This study |
| BL21-pET-rHb_6th_- CysG^A^ | BL21(DE3) harboring plasmid  pET-rHb_6th_-CysG^A^, Ap^R^ | This study |
| BL21-pET-rHb_1st_ | BL21(DE3) harboring plasmid  pET-rHb_1st_, Ap^R^ | This study |
| BL21-pET-rHb_2nd_ | BL21(DE3) harboring plasmid  pET-rHb_2nd_, Ap^R^ | This study |
| BL21-pET-rHb_3rd_ | BL21(DE3) harboring plasmid  pET-rHb_3rd_, Ap^R^ | This study |
| BL21-pET-rHb_4th_ | BL21(DE3) harboring plasmid  pET-rHb_4th_, Ap^R^ | This study |
| BL21-pET-rHb_5th_ | BL21(DE3) harboring plasmid  pET-rHb_5th_, Ap^R^ | This study |
| BL21-pET-rHb_6th_ | BL21(DE3) harboring plasmid  pET-rHb_6th_, Ap^R^ | This study |
| R11-pET-rHb_α-F33_ | R11-pTf16-HS_M7A_-RSF harboring plasmid  pET-rHb_α-F33_, Cm^R^, Ap^R^ | This study |
| R11-pET-rHb_α-Y42_ | R11-pTf16-HS_M7A_-RSF harboring plasmid  pET-rHb_α-Y42_, Cm^R^, Ap^R^ | This study |
| R11-pET-rHb_α-F43_ | R11-pTf16-HS_M7A_-RSF harboring plasmid  pET-rHb_α-F43_, Cm^R^, Ap^R^ | This study |
| R11-pET-rHb_β-W15_ | R11-pTf16-HS_M7A_-RSF harboring plasmid  pET-rHb_β-W15_, Cm^R^, Ap^R^ | This study |
| R11-pET-rHb_β-S44_ | R11-pTf16-HS_M7A_-RSF harboring plasmid  pET-rHb_β-S44_, Cm^R^, Ap^R^ | This study |
| R11-pET-rHb_β-P51_ | R11-pTf16-HS_M7A_-RSF harboring plasmid  pET-rHb_β-P51_, Cm^R^, Ap^R^ | This study |
| R11-pET-rHb_β-K66_ | R11-pTf16-HS_M7A_-RSF harboring plasmid  pET-rHb_β-K66_, Cm^R^, Ap^R^ | This study |
| R11-pET-rHb_β-K82_ | R11-pTf16-HS_M7A_-RSF harboring plasmid  pET-rHb_β-K82_, Cm^R^, Ap^R^ | This study |
| R11-pET-rHb_β-D99_ | R11-pTf16-HS_M7A_-RSF harboring plasmid  pET-rHb_β-D99_, Cm^R^, Ap^R^ | This study |
| R11-pET-rHb_7th_ | R11-pTf16-HS_M7A_-RSF harboring plasmid  pET-rHb_7th_, Cm^R^, Ap^R^ | This study |
| R11-pET-rHb_8th_ | R11-pTf16-HS_M7A_-RSF harboring plasmid  pET-rHb_8th_, Cm^R^, Ap^R^ | This study |
| R11-pET-rHb_9th_ | R11-pTf16-HS_M7A_-RSF harboring plasmid  pET-rHb_9th_, Cm^R^, Ap^R^ | This study |
| BL21-pET-rHb_7th_- CysG^A^ | BL21(DE3) harboring plasmid  pET-rHb_7th_-CysG^A^, Ap^R^ | This study |
| BL21-pET-rHb_8th_- CysG^A^ | BL21(DE3) harboring plasmid  pET-rHb_8th_-CysG^A^, Ap^R^ | This study |
| BL21-pET-rHb_9th_- CysG^A^ | BL21(DE3) harboring plasmid  pET-rHb_9th_-CysG^A^, Ap^R^ | This study |
| BL21-pET-sMb | BL21(DE3) harboring plasmid  pET-sMb, Ap^R^ | This study |
| BL21-pET-rHb_10th_ | BL21(DE3) harboring plasmid  pET-rHb_10th_, Ap^R^ | This study |
| BL21-pET-rHb_11th_ | BL21(DE3) harboring plasmid  pET-rHb_11th_, Ap^R^ | This study |
| BL21-pET-rHb_12th_ | BL21(DE3) harboring plasmid  pET-rHb_12th_, Ap^R^ | This study |
| BL21-pET-rHb_13th_ | BL21(DE3) harboring plasmid  pET-rHb_13th_, Ap^R^ | This study |
| BL21-pET-rHb_14th_ | BL21(DE3) harboring plasmid  pET-rHb_14th_, Ap^R^ | This study |
| BL21-pET-rHb_15th_ | BL21(DE3) harboring plasmid  pET-rHb_15th_, Ap^R^ | This study |
| BL21-pET-rHb_16th_ | BL21(DE3) harboring plasmid  pET-rHb_16th_, Ap^R^ | This study |
| BL21-pET-rHb_10th_-CysG^A^ | BL21(DE3) harboring plasmid  pET-rHb_10th_-CysG^A^, Ap^R^ | This study |
| BL21-pET-rHb_11th_-CysG^A^ | BL21(DE3) harboring plasmid  pET-rHb_11th_-CysG^A^, Ap^R^ | This study |
| BL21-pET-rHb_12th_-CysG^A^ | BL21(DE3) harboring plasmid  pET-rHb_12th_-CysG^A^, Ap^R^ | This study |
| BL21-pET-rHb_13th_-CysG^A^ | BL21(DE3) harboring plasmid  pET-rHb_13th_-CysG^A^, Ap^R^ | This study |
| BL21-pET-rHb_14th_-CysG^A^ | BL21(DE3) harboring plasmid  pET-rHb_14th_-CysG^A^, Ap^R^ | This study |
| BL21-pET-rHb_15th_-CysG^A^ | BL21(DE3) harboring plasmid  pET-rHb_15th_-CysG^A^, Ap^R^ | This study |
| BL21-pET-rHb_16th_-CysG^A^ | BL21(DE3) harboring plasmid  pET-rHb_16th_-CysG^A^, Ap^R^ | This study |
| C41-pRSF-  rHb_wild-type_-MAP | C41(DE3) harboring plasmid  pRSF-rHb_wild-type_-MAP, Km^R^ | This study |
| C41-pRSF-rHb_13th_-MAP | C41(DE3) harboring plasmid  pRSF-rHb_13th_-MAP, Km^R^ | This study |
| R11-pRSF-  rHb_wild-type_-MAP | HEME-R11 harboring plasmid  pRSF-rHb_wild-type_-MAP, Cm^R^, Km^R^ | This study |
| R11-pRSF-rHb_13th_-MAP | HEME-R11 harboring plasmid  pRSF-rHb_13th_-MAP, Cm^R^, Km^R^ | This study |

Table S3. The primers used in the study

| Primers | Sequence (5'-3') ^a^ |
| --- | --- |
| pET-rHb_wild-type_-F1 | TATAAGATAGGAGGAATTCCATATGGTACTATCACCCGCTGATAAAACAAATG |
| pET-rHb_wild-type_-R1 | CCGTTCTGACCAGCAAATACCGTTAAGCATAATGCTTAAGTC |
| pET-rHb_wild-type_-F2 | GAAGGAGATATACAATGGTACACCTAACACCCGAGG |
| pET-rHb_wild-type_-R2 | TCTCATCCGCCCCGCTCGAGTTAGTGATGATGATGGTGGTGGTGATGATGATGATGGTATTTGTGCGCCAACGCATTAG |
| pET-MAP-F | GTTTAACTTTAAGAAGGAGATATACCATGGCTATCTCAATCAAGACCCCAG |
| pET-MAP-R | ATCCGCCCGCGGATCCTTATTCGTCGTGCGAGATTATCGCC |
| pET-rHb_wild-type_-CysG^A^_(G4S)2_-F1 | GTTTAACTTTAAGAAGGAGATATACATGGAAACGACCGAACAGTTAATCAACGAAC |
| pET-rHb_wild-type_-CysG^A^_(G4S)2_-R1 | CATACCGCTGCTGCCTGAGCTGCCACCGGTTTTTAAGTGTCCG |
| pET-rHb_wild-type_-CysG^A^_(G4S)2_-F2 | GACACTTAAAAACCGGTGGCAGCTCAGGCAGCAGCGGTATGGTACTATCACCCGCTGATAAAAC |
| pET-rHb_wild-type_-CysG^A^_(G4S)2_-R2 | CAGCTCGCTACCCCCGCCGCCTGAACCGCCACCACCATGGTATTTGTGCGCCAACGC |
| pET-rHb_wild-type_-CysG^A^_(G4S)2_-F3 | GGTTCAGGCGGCGGGGGTAGCGAGCTGGACTGGGAAAACCTGG |
| pET-rHb_wild-type_-CysG^A^_(G4S)2_-R3 | CGCAGCAGCGGTTTCTTTACCAGACTTAATGGTTGGAGAACCAGTTCAGTTTATCG |
| pET-rHb_wild-type_-CysG^A^_G4S_-F | CTAATGCGTTGGCGCACAAATACCATGGTGGTGGCGGGTCTGAGCTG |
| pET-rHb_wild-type_-CysG^A^_G4S_-R | CTAATGCGTTGGCGCACAAATACCAT |
| pET-rHb_wild-type_-  CysG^A^ _(G4S)3_-F | CGTTGGCGCACAAATACCATGGAGGTGGCGGATCTGGTGGTGGCGGTTCAGGCGG |
| pET-rHb_wild-type_-  CysG^A^ _(G4S)3_-R | CTAATGCGTTGGCGCACAAATACCAT |
| pET-rHb_wild-type_-  CysG^A^ _(S2G)2_-F | GGATCCTCGGGATCATCAGAGCTGGACTGGGAAAACCTGG |
| pET-rHb_wild-type_-  CysG^A^ _(S2G)2_-R | CTGATGATCCCGAGGATCCATGGTATTTGTGCGCCAACGCATTAG |
| pET-rHb_wild-type_-  CysG^A^ _(G2S4)9_-F | CATCAGGGGGATCCTCTAGCTCAGAGCTGGACTGGGAAAACCTGG |
| pET-rHb_wild-type_-  CysG^A^ _(G2S4)9_-R | GAGCTAGAGGATCCCCCATGGTATTTGTGCGCCAACGCATTAG |
| pET-rHb_wild-type_-CysG^A^_GGS10_-F | GGCGGGTCCGGCGGTTCAGGTGGGAGCGGTGAGCTGGACTGGGAAAACCTG |
| pET-rHb_wild-type_-CysG^A^_GGS10_-R | ACCGCTCCCACCTGAACCGCCGGACCCGCCATGGTATTTGTGCGCCAACGC |
| pET-CysG^A^-  rHb_α-P119S_-F | GCGGAATTCACTAGCGCGGTTCATGCGAGCCTGGATAAATTC |
| pET-CysG^A^-  rHb-_αP119S_-R | GCATGAACCGCGCTAGTGAATTCCGCCGGCAGGTG |
| pET-CysG^A^-  rHb_α-L29W_-F | GCGTGGGAGCGCATGTTCCTGTCTTTTCC |
| pET-CysG^A^-  rHb_α-L29W_-R | GAACATGCGCTCCCACGCCTCAGCACCATATTCACC |
| pET-CysG^A^-  rHb_β-L28W_-F | GTGGTGAAGCATGGGGTCGTTTGCTGGTCGTGTACC |
| pET-CysG^A^-  rHb_β-L28W_-R | CAGCAAACGACCCCATGCTTCACCACCAACCTCGTC |
| pET-CysG^A^-  rHb_α-G15A_-F | GCGGCTTGGGCCAAGGTGGGTGCGCACGCC |
| pET-CysG^A^-  rHb_α-G15A_-R | GCACCCACCTTGGCCCAAGCCGCCTTAACATTTGTTTTATC |
| pET-CysG^A^-  rHb_β-G16A_-F | GACTGCGCTGTGGGCCAAGGTGAATGTTGACGAGGTTGG |
| pET-CysG^A^-  rHb_β-G16A_-R | CAACATTCACCTTGGCCCACAGCGCAGTCACAGCTG |
| pET-CysG^A^-  rHb_β-H116I_-F | GTTTGTGTTTTGGCGATTCATTTTGGCAAGGAGTTCACCC |
| pET-CysG^A^-  rHb_β-H116I_-R | CCTTGCCAAAATGAATCGCCAAAACACAAACTAACACGTTG |
| pTf16-HS_M7A_-F | GATTTTTTGAGGTAACAAGCACATGGTTTCTGAACTGATCAAAGAAAAC |
| pTf16-HS_M7A_-R | GCGCGTTATGCTGCGTAAATTATTTGTATAGTTCATCCATGCCATGTGTAATC |
| pTf16- HS_M7A_-RSF-F | ATTAATAAGATGATCTTCCGCTCTTCCGCTTCCTCGCTCACTG |
| pTf16- HS_M7A_-RSF-R | ATTGCACTGAAATCTAGAGTAACGGAATAGCTGTTCGTTGACTTG |
| pET-  rHb_β-F41Y_-F | GAAGCTCTCGAAGTAACGTTGGGTCCACGGGTACAC |
| pET-  rHb_β-F41Y_-R | CCGTGGACCCAACGTTACTTCGAGAGCTTCGGCGATCTG |
| pET-  rHb_β-T84Y_-F | GACAACCTGAAAGGTTACTTTGCGACCCTCTCTGAACTGC |
| pET-  rHb_β-T84Y_-R | CAGAGAGGGTCGCAAAGTAACCTTTCAGGTTGTCCAGGTGG |
| pET-  rHb_β-H92Q_-F | CCTCTCTGAACTGCAGTGCGACAAGCTGTGGGTTGATC |
| pET-  rHb_β-H92Q_-R | CACAGCTTGTCGCACTGCAGTTCAGAGAGGGTCGCAAACG |
| pET-  rHb_β-S44H_-F | ACGTTTTTTCGAGCATTTCGGCGATCTGTCCACCCCG |
| pET-  rHb_β-S44H_-R | GACAGATCGCCGAAATGCTCGAAAAAACGTTGGGTCCACG |
| pET-  rHb_β-V67T_-F | GCACGGCAAGAAGACCTTGGGCGCATTTAGCGACGG |
| pET-  rHb_β-V67T_-R | CTAAATGCGCCCAAGGTCTTCTTGCCGTGCGCTTTTAC |
| pET-  rHb_α-H58L_-F | CGCACAGGTCAAAAAGCTGGGCAAGAAGGTCGCAGATGC |
| pET-  rHb_α-H58L_-R | ACCTTCTTGCCCAGCTTTTTGACCTGTGCGCTGCC |
| pET-  rHb_β-K82D_-F | CCTGGACAACCTGGATGGTACGTTTGCGACCCTCTC |
| pET-  rHb_β-K82D_-R | GTCGCAAACGTACCATCCAGGTTGTCCAGGTGGGCCAG |
| pET-CysG^A^-  rHb_α-G15_-F | AACAAATGTTAAGGCGGCTTGGNNKAAGGTGGGTGCGCACGCCG |
| pET-CysG^A^-  rHb_α-G15_-R | CCAAGCCGCCTTAACATTTGTTTTATCAGCG |
| pET-CysG^A^-  rHb_α-K16_-F | AACAAATGTTAAGGCGGCTTGGGGTNNKGTGGGTGCGCACGCCG |
| pET-CysG^A^-  rHb_α-K16_-R | ACCCCAAGCCGCCTTAACATTTG |
| pET-CysG^A^-  rHb_α-G25_-F | GCACGCCGGTGAATATNNKGCTGAGGCGCTTGAGCGCATG |
| pET-CysG^A^-  rHb_α-G25_-R | ATATTCACCGGCGTGCGCACC |
| pET-CysG^A^-  rHb_α-G57_-F | TCCCACGGCAGCGCACAGGTCAAANNKCATGGCAAGAAGGTCGCAGATG |
| pET-CysG^A^-  rHb_α-G57_-R | TTTGACCTGTGCGCTGCCG |
| pET-CysG^A^-  rHb_α-V93_-F | GATCTGCATGCGCACAAACTGCGTNNKGACCCGGTAAACTTTAAGTTGCTGAGCC |
| pET-CysG^A^-  rHb_α-V93_-R | ACGCAGTTTGTGCGCATGCA |
| pET-CysG^A^-  rHb_α-D94_-F | GATCTGCATGCGCACAAACTGCGTGTGNNKCCGGTAAACTTTAAGTTGCTGAGCC |
| pET-CysG^A^-  rHb_α-D94_-R | CACACGCAGTTTGTGCGCATG |
| pET-CysG^A^-  rHb_α-P114_-F | GCCGCGCACCTGNNKGCGGAATTCACTCCGGCG |
| pET-CysG^A^-  rHb_α-P114_-R | GTGAATTCCGCNNKCAGGTGCGCGGCCAGGGTA |
| pET-CysG^A^-  rHb_β-H2_-F | ATAAGAAGGAGATATACAATGGTANNKCTAACACCCGAGGAAAAATCAGCTG |
| pET-CysG^A^-  rHb_β-H2_-R | TACCATTGTATATCTCCTTCTTATACTTAACTAATATACTAAGATGG |
| pET-CysG^A^-  rHb_β-G16_-F | AAATCAGCTGTGACTGCGCTGTGGNNKAAGGTGAATGTTGACGAGGTTGG |
| pET-CysG^A^-  rHb_β-G16_-R | ACATTCACCTTNNKCCACAGCGCAGTCACAGCTG |
| pET-CysG^A^-  rHb_β-G69_-F | GAAGGTGTTGNNKGCATTTAGCGACGGCCTGG |
| pET-CysG^A^-  rHb_β-G69_-R | GTCGCTAAATGCNNKCAACACCTTCTTGCCGTGCG |
| pET-CysG^A^-  rHb_β-S72_-F | TTGGGCGCATTTNNKGACGGCCTGGCCCACCTG |
| pET-CysG^A^-  rHb_βS-72_-R | CCAGGCCGTCNNKAAATGCGCCCAACACCTTCTTG |
| pET-CysG^A^-  rHb_β-H97_-F | TGAACTGCACTGCGACAAGCTGNNKGTTGATCCGGAAAACTTCCGCC |
| pET-CysG^A^-  rHb_β-H97_-R | GGAAGTTTTCCGGATCAACNNKCAGCTTGTCGCAGTGCAGTTC |
| pET-CysG^A^-  rHb_β-N102_-F | ATGTTGATCCGGAANNKTTCCGCCTGCTTGGCAACG |
| pET-CysG^A^-  rHb_β-N102_-R | GTTGCCAAGCAGGCGGAANNKTTCCGGATCAACATGCAGCTTG |
| pET-rHb_α-P114I_-F | GCCGCGCACCTGATTGCGGAATTCACTCCGGCGG |
| pET-rHb_α-P114I_-R | CCGGAGTGAATTCCGCAATCAGGTGCGCGGCCAGGGTAAC |
| pET-rHb_α-G25C_-F | GCACGCCGGTGAATATTGTGCTGAGGCGCTTGAGCGCATG |
| pET-rHb_α-G25C_-R | AAGCGCCTCAGCACAATATTCACCGGCGTGCGCACC |
| pET-rHb_β-H2E_-F | AGATATACAATGGTAGAGCTAACACCCGAGGAAAAATCAGCTG |
| pET-rHb_β-H2E_-R | CGGGTGTTAGCTCTACCATTGTATATCTCCTTCTTATACTTAACTAATATAC |
| pET-rHb_α-G57K_-F | CAGCGCACAGGTCAAAAAGCATGGCAAGAAGGTCGCAGATG |
| pET-rHb_α-G57K_-R | CCTTCTTGCCATGCTTTTTGACCTGTGCGCTGCCGTG |
| pET-rHb_β-N102T_-F | GCTGCATGTTGATCCGGAAACTTTCCGCCTGCTTGGCAACGTG |
| pET-rHb_β-N102T_-R | GGCGGAAAGTTTCCGGATCAACATGCAGCTTG |
| pET-rHb_β-H97W_-F | CACTGCGACAAGCTGTGGGTTGATCCGGAAAACTTCCGCC |
| pET-rHb_β-H97W_-R | CCGGATCAACCCACAGCTTGTCGCAGTGCAGTTC |
| pET-rHb_α-F33_-F | CTTGAGCGCATGNNKCTGTCTTTTCCAACGACCAAAACCTAC |
| pET-rHb_α-F33_-R | CGTTGGAAAAGACAGMNNCATGCGCTCAAGCGCCTCAG |
| pET-rHb_α-Y42_-F | CGACCAAAACCNNKTTCCCGCATTTTGACCTGTCC |
| pET-rHb_α-Y42_-R | GGTCAAAATGCGGGAAMNNGGTTTTGGTCGTTGGAAAAGACAGG |
| pET-rHb_α-F43_-F | GACCAAAACCTACNNKCCGCATTTTGACCTGTCCCAC |
| pET-rHb_α-F43_-R | CAGGTCAAAATGCGGMNNGTAGGTTTTGGTCGTTGGAAAAGACAG |
| pET-rHb_β-W15_-F | CTGTGACTGCGCTGNNKGGTAAGGTGAATGTTGACGAGGTTG |
| pET-rHb_β-W15_-R | CATTCACCTTACCMNNCAGCGCAGTCACAGCTGATTTTTC |
| pET-rHb_β-S44_-F | CCAACGTTTTTTCGAGNNKTTCGGCGATCTGTCCACCCCG |
| pET-rHb_β-S44_-R | GATCGCCGAAMNNCTCGAAAAAACGTTGGGTCCACG |
| pET-rHb_β-P51_-F | GATCTGTCCACCNNKGATGCAGTTATGGGTAACCCGAAAG |
| pET-rHb_β-P51_-R | ACCCATAACTGCATCMNNGGTGGACAGATCGCCGAAG |
| pET-rHb_β-K66_-F | CGCACGGCAAGNNKGTGTTGGGCGCATTTAGCGAC |
| pET-rHb_β-K66_-R | CTAAATGCGCCCAACACMNNCTTGCCGTGCGCTTTTACTTTCG |
| pET-rHb_β-K82_-F | GGACAACCTGNNKGGTACGTTTGCGACCCTCTCTG |
| pET-rHb_β-K82_-R | GAGGGTCGCAAACGTACCMNNCAGGTTGTCCAGGTGGGCCAG |
| pET-rHb_β-D99_-F | GACAAGCTGTGGGTTNNKCCGGAAACTTTCCGCCTGCTTG |
| pET-rHb_β-D99_-R | GAAAGTTTCCGGMNNAACCCACAGCTTGTCGCAGTG |
| pET-rHb_α-Y42S_-F | CGACCAAAACCAGTTTCCCGCATTTTGACCTGTCC |
| pET-rHb_α-Y42S_-R | GTCAAAATGCGGGAAACTGGTTTTGGTCGTTGGAAAAGACAGGAAC |
| pET-rHb_β-P51N_-F | GATCTGTCCACCAATGATGCAGTTATGGGTAACCCGAAAG |
| pET-rHb_β-P51N_-R | CATAACTGCATCATTGGTGGACAGATCGCCG |
| pET-rHb_β-S44D_-F | CAACGTTTTTTCGAGGATTTCGGCGATCTGTCCACC |
| pET-rHb_β-S44D_-R | GACAGATCGCCGAAATCCTCGAAAAAACGTTGGGTCCACG |
| pET-sMb-F1 | TTAAGAAGGAGATATACCATGGTGCTGAGCGAAGGCGAATG |
| pET-sMb-R1 | CCCAAGGGGTTATGCTAGTTATTTAGTGATGATGATGGTGGTGGTGATG |
| pET-rHb_α-K60G_-F | CAATGCATCTGCGACCTTACCGCCATGCTTTTTGACCTGTGCG |
| pET-rHb_α-K60G_-R | TCAAAAAGCATGGCGGTAAGGTCGCAGATGCATTGACGAAC |
| pET-rHb_α-A71G_-F | CATGTCGTCAACGTGGGTCACCGCGTTCGTCAATGCAT |
| pET-rHb_α-A71G_-R | CATTGACGAACGCGGTGACCCACGTTGACGACATGCCGAATG |
| pET-  rHb_β-V1M/T4I/P5A_-F | AGGAGATATACAATGATGGAGCTAATTGCTGAGGAAAAATCAGCTGTGACTGCG |
| pET-  rHb_β-V1M/T4I/P5A_-R | CCTCAGCAATTAGCTCCATCATTGTATATCTCCTTTAACTAATATACTAAGATG |
| pET-rHb_β-A76K_-F | CAGGTTGTCCAGGTGTTTCAGGCCGTCGCTAAATGCGCCCAAC |
| pET-rHb_β-A76K_-R | CATTTAGCGACGGCCTGAAACACCTGGACAACCTGAAAGGTACGTTTGCG |
| pET-rHb_β-T4S/P5G_-F | AGCTGATTTTTCCTCACCGCTTAGCTCTACCATTGTATATCTCCTTCTTATACTTAAC |
| pET-rHb_β-T4S/P5G_-R | CAATGGTAGAGCTAAGCGGTGAGGAAAAATCAGCTGTGACTGCG |
| pET-rHb_α-V1E_-F | GATAGTTCCATGTATATCTCCTTCTTAAAGTTAAACAAAATTATTTCTAG |
| pET-rHb_α-V1E_-R | GTTTAACTTTAAGAAGGAGATATACATGGAACTATCACCCGCTGATAAAACAAATG |
| pET-rHb_β-N108K_-F | CAAAACACAAACTAACACCTGGCCAAGCAGGCGGAAAGTTTC |
| pET-rHb_β-N108K_-R | TCCGCCTGCTTGGCCAGGTGTTAGTTTGTGTTTTGGCGCAC |
| pET-rHb  _β-V1M/E2deleted/T4I/P5A_-F | AGGAGATATACAATGATGCTAATTGCGGAGGAAAAATCAGCTGTGACTGCG |
| pET-rHb  _β-V1M/E2deleted/T4I/P5A_-R | TTCCTCCGCAATTAGCATCATTGTATATCTCCTTCTTATACTTAACTAATATACTAAG |
| pET-rHb  _β-0Minserted/T4S/P5G_-F | TGATGGTAGAGCTAAGCGGTGAGGAAAAATCAGCTGTGACTGCG |
| pET-rHb  _β-0Minserted/T4S/P5G_-R | AGCTGATTTTTCCTCACCGCTTAGCTCTACCATCATTGTATATCTCCTTCTTATACTTAAC |
| pET-rHb_β-H116R_-F | GTTTTGGCGCGCCATTTTGGCAAGGAGTTCACCCCAC |
| pET-rHb_β-H116R_-R | GTGAACTCCTTGCCAAAATGGCGCGCCAAAACACAAACTAACACGTTGC |
| pRSF-MAP-F | AAGAAGGAGATATACCATGGCTATCTCAATCAAGACCCCAG |
| pRSF-MAP-R | CTAATGCAGGAGTCGCATAAGGGTTATTCGTCGTGCGAGATTATCGCC |
| pRSF-rHb_wild-type_-  MAP-F | AATAAGGAGATATACCATGGTACTATCACCCGCTGATAAAAC |
| pRSF-rHb_wild-type_-  MAP-R | TTACCAGACTCGAGTTAGTGATGATGATGGTGGTGGTGATG |
| pRSF-rHb_13th_-  MAP-F | AATAAGGAGATATACCATGGAACTATCACCCGCTGATAAAAC |
| pRSF-rHb_13th_-  MAP-R | CTTTACCAGACTCGAGTTAGTGATGATGATGGTGGTGGTGATG |

a) The mutation sites were shown in underline.

Table S4. The linkers with different length and glycine ratio used for C-terminal fusion expression of CysG^A^-β-globin with rHb

| Index | Linker | Linker sequence | The percent of glycine (%) | Length |
| --- | --- | --- | --- | --- |
| 1 | (S_2_G)_2_ | SSGSSG | 33 | 6 |
| 2 | (G_2_S_4_)_9_ | (GGSSSS)_9_ | 33 | 54 |
| 3 | GGS_10_ | GGSGGSGGSG | 70 | 10 |
| 4 | G_4_S | GGGGS | 80 | 5 |
| 5 | (G_4_S)_2_ | GGGGSGGGGS | 80 | 10 |
| 6 | (G_4_S)_3_ | GGGGSGGGGSGGGGS | 80 | 15 |

Table S5. Stability mutation energy of rHb variants

| Index | Mutation site | Mutation Energy (kcal/mol) | Source |
| --- | --- | --- | --- |
| 1 | α-P119S | 2.2 | (Looker, et al., 1992) |
| 2 | α-L29W | 0.8 | (Dou, et al., 2002) |
| 3 | β-L28W | 0.4 | (Varnado, et al., 2013) |
| 4 | wild-type | 0.0 | This study |
| 5 | α-G15A | -1.4 | (Graves, et al., 2008) |
| 6 | α-G15A/β-G16A | -2.6 | (Graves, et al., 2008) |
| 7 | α-G15A/β-G16A/β-H116I | -3.4 | (Graves, et al., 2008) |

Table S6. Binding Mutant Energy of rHb variants

| Index | Mutation site | Binding Mutation Energy (kcal/mol) | Source |
| --- | --- | --- | --- |
| 1 | β-H92Q | 0.18 | (de Weinstein, et al., 2000) |
| 2 | α-H58L | 0.04 | (Bissé, et al., 2017) |
| 3 | wild-type | 0.00 | This study |
| 4 | β-V67T | -0.04 | (Gattoni, et al., 2001) |
| 5 | β-F41Y | -0.04 | (Silkstone, et al., 2016) |
| 6 | β-T84Y | -0.10 | (Cooper, et al., 2019) |
| 7 | β-K82D | -0.14 | (Varnado, et al., 2013) |
| 8 | β-S44H | -0.26 | (Varnado, et al., 2013) |

Table S7. The autoxidation rate of rHb variants expressed in *E. coli*

| Protein | *k*_autox_ (h^-1^) |
| --- | --- |
| rHb_wild-type_ | 0.17 ± 0.03 |
| rHb_1st_ | 0.03 ± 0.00 |
| rHb_2nd_ | 0.03 ± 0.00 |
| rHb_3rd_ | 0.02 ± 0.00 |
| rHb_4th_ | 0.02 ± 0.00 |
| rHb_5th_ | 0.02 ± 0.01 |
| rHb_6th_ | 0.01 ± 0.01 |

Table S8. The autoxidation and heme loss rates of rHb variants with enhanced heme-binding capability in *E. coli*

| Protein | *k*_autox_ (h^-1^) | *k*_-H_ α (h^-1^) | *k*_-H_ β (h^-1^) |
| --- | --- | --- | --- |
| rHb_wild-type_ | 0.12 ± 0.03 | 0.31 ± 0.02 | 3.04 ± 0.16 |
| rHb_6th_ | 0.01 ± 0.00 | 0.12 ± 0.02 | 1.85 ± 0.25 |
| rHb_7th_ | 0.01 ± 0.00 | 0.06 ± 0.01 | 2.13 ± 0.16 |
| rHb_8th_ | 0.01 ± 0.00 | 0.06 ± 0.01 | 2.27 ± 0.18 |
| rHb_9th_ | 0.01 ± 0.00 | 0.02 ± 0.00 | 1.32 ± 0.10 |

Table S9. The properties of rHb variants with enhanced oxygen-carrying capacity in *E. coli*, including the rate of autoxidation (*k*_autox_ (h^-1^)), the rate of heme loss (*k*_-H_ α (h^-1^), *k*_-H_ β (h^-1^)), oxygen equilibration curve (*P*_50_) and Hill coefficient (*n*_Hill_)

| Protein | *k*_autox_ (h^-1^) | *k*_-H_ α (h^-1^) | *k*_-H_ β (h^-1^) | *P*_50_ (mmHg) | *n*_Hill_ |
| --- | --- | --- | --- | --- | --- |
| rHb_wild-type_ | 0.22 ± 0.05 | 0.29 ± 0.02 | 2.87 ± 0.11 | 13.1 ± 0.2 | 1.8 ± 0.1 |
| rHb_6th_ | 0.02 ± 0.00 | 0.12 ± 0.01 | 1.89 ± 0.15 | 17.7 ± 0.6 | 1.4 ± 0.1 |
| rHb_9th_ | 0.01 ± 0.00 | 0.03 ± 0.01 | 1.28 ± 0.04 | 20.1 ± 0.3 | 1.4 ± 0.1 |
| rHb_10th_ | 0.02 ± 0.00 | 0.12 ± 0.01 | 2.00 ± 0.18 | 28.4 ± 0.8 | 1.6 ± 0.1 |
| rHb_12th_ | 0.02 ± 0.00 | 0.09 ± 0.01 | 1.78 ± 0.14 | 13.8 ± 1.3 | 1.4 ± 0.0 |
| rHb_13th_ | 0.02 ± 0.00 | 0.04 ± 0.01 | 1.45 ± 0.16 | 24.0 ± 0.2 | 2.1 ± 0.1 |
| rHb_14th_ | 0.03 ± 0.00 | 0.15 ± 0.01 | 2.00 ± 0.22 | 14.5 ± 0.5 | 1.5 ± 0.1 |

Table S10. The properties of rHb variants in heme-supply enhanced *E. coli*, including the rate of autoxidation (*k*_autox_ (h^-1^)), the rate of heme loss (*k*_-H_ α (h^-1^), *k*_-H_ β (h^-1^)), oxygen equilibration curve (*P*_50_) and Hill coefficient (*n*_Hill_)

| Strains | Protein | *k*_autox_ (h^-1^) | *k*_-H_ α (h^-1^) | *k*_-H_ β (h^-1^) | *P*_50_ (mmHg) | *n*_Hill_ |
| --- | --- | --- | --- | --- | --- | --- |
| HEME-R11 | rHb_wild-type_ | 0.26 ± 0.07 | 0.33 ± 0.14 | 3.55 ± 0.38 | 13.1 ± 0.1 | 2.3 ± 0.0 |
| HEME-R11 | rHb_13th_ | 0.02 ± 0.00 | 0.03 ± 0.01 | 2.08 ± 0.24 | 24.1 ± 0.3 | 2.8 ± 0.1 |
| BL21(DE3) | rHb_13th_ | 0.02 ± 0.00 | 0.05 ± 0.01 | 1.79 ± 0.20 | 24.0 ± 0.2 | 2.6 ± 0.1 |

**Supplementary Notes**

**1. Sequence** **of gene encoding methionine aminopeptidase**

Promoter: highlighted in yellow

Gene coding region: red font

Terminator: highlighted in blue

TTGACAATTAATCATCGGCTCGTATAATGGGGGAATTGTGAGCGGATAACAATTCCCCTCTAGAAATAATTTTGTTTAACTTTAAGAAGGAGATATACATGCCATGGCTATCTCAATCAAGACCCCAGAAGATATCGAAAAAATGCGCGTCGCTGGCCGACTGGCTGCCGAAGTGCTGGAGATGATCGAACCGTATGTTAAACCGGGCGTCAGCACCGGCGAGCTGGATCGCATCTGTAATGATTACATTGTTAATGAACAACACGCGGTTTCTGCCTGCCTCGGCTATCACGGCTATCCGAAATCCGTTTGCATCTCTATTAATGAAGTGGTGTGCCACGGTATCCCGGACGATGCTAAGCTGCTGAAAGATGGCGATATCGTTAACATTGATGTCACCGTAATCAAAGATGGTTTCCACGGCGATACCTCGAAAATGTTTATCGTCGGTAAGCCGACCATCATGGGCGAACGTCTGTGCCGCATCACGCAAGAAAGCCTGTACCTGGCGCTACGCATGGTAAAACCAGGCATTAATCTGCGCGAAATCGGTGCGGCGATTCAGAAATTTGTCGAAGCAGAAGGCTTCTCCGTCGTTCGTGAATATTGCGGACACGGTATTGGTCGCGGCTTCCATGAAGAACCGCAGGTGCTGCACTATGACTCCCGTGAAACCAACGTCGTACTGAAACCTGGGATGACGTTCACCATCGAGCCAATGGTCAACGCGGGTAAAAAAGAGATCCGCACCATGAAAGATGGCTGGACGGTAAAAACCAAAGATCGCAGCTTGTCTGCACAATATGAGCATACTATTGTGGTGACTGATAACGGCTGCGAAATTCTGACGCTACGCAAGGATGACACCATCCCGGCGATAATCTCGCACGACGAATAAGGATCCGCGGGCGGATGAGAGAAGATTTTCAGCCTGATACAGATTAAATCAGAACGCAGAAGCGGTCTGATAAAACAGAATTTGCCTGGCGGCAGTAGCGCGGTGGTCCCACCTGACCCCATGCCGAACTCAGAAGTGAAACGCCGTAGCGCCGATGGTAGTGTGGGGTCTCCCCATGCGAGAGTAGGGAACTGCCAGGCATCAAATAAAACGAAAGGCTCAGTCGAAAGACTGGGCCTTTCGTTTTATCTGTTGTTTGTCGGTGAACGCTCTCCTGAGTAGGACAAATCCGCCGGGAGCGGATTTGAACGTTGCGAAGCAACGGCCCGGAGGGTGGCGGGCAGGACGCCCGCCATAAACTGCCAGGCATCAAATTAAGCAGAAGGCCATCCTGACGGATGGCCTTTTTGCGTTTC

**2. Sequence of gene encoding rHb_wild-type_ expressed in *E. coil***

Promoter: highlighted in yellow

α-globin gene coding region: red font

β-globin gene coding region: orange font

His Tag: highlighted in green

Terminator: highlighted in blue

TAATACGACTCACTATAGGGGAATTGTGAGCGGATAACAATTCCCCTCTAGAAATAATTTTGTTTAACTTTAAGAAGGAGATATACATGGTACTATCACCCGCTGATAAAACAAATGTTAAGGCGGCTTGGGGTAAGGTGGGTGCGCACGCCGGTGAATATGGTGCTGAGGCGCTTGAGCGCATGTTCCTGTCTTTTCCAACGACCAAAACCTACTTCCCGCATTTTGACCTGTCCCACGGCAGCGCACAGGTCAAAGGCCATGGCAAGAAGGTCGCAGATGCATTGACGAACGCGGTGGCCCACGTTGACGACATGCCGAATGCATTGTCGGCTCTCTCTGATCTGCATGCGCACAAACTGCGTGTGGACCCGGTAAACTTTAAGTTGCTGAGCCACTGCCTGTTGGTTACCCTGGCCGCGCACCTGCCGGCGGAATTCACTCCGGCGGTTCATGCGAGCCTGGATAAATTCCTGGCTTCCGTGAGCACCGTTCTGACCAGCAAATACCGTTAAGCATAATGCTTAAGTCGAACAGAAAGTAATCGTATTGTACACGGCCGCATAATCGAAATTAATACGACTCACTATAGGGGAATTGTGAGCGGATAACAATTCCCCATCTTAGTATATTAGTTAAGTATAAGAAGGAGATATACAATGGTACACCTAACACCCGAGGAAAAATCAGCTGTGACTGCGCTGTGGGGTAAGGTGAATGTTGACGAGGTTGGTGGTGAAGCACTGGGTCGTTTGCTGGTCGTGTACCCGTGGACCCAACGTTTTTTCGAGAGCTTCGGCGATCTGTCCACCCCGGATGCAGTTATGGGTAACCCGAAAGTAAAAGCGCACGGCAAGAAGGTGTTGGGCGCATTTAGCGACGGCCTGGCCCACCTGGACAACCTGAAAGGTACGTTTGCGACCCTCTCTGAACTGCACTGCGACAAGCTGCATGTTGATCCGGAAAACTTCCGCCTGCTTGGCAACGTGTTAGTTTGTGTTTTGGCGCACCATTTTGGCAAGGAGTTCACCCCACCGGTTCAGGCTGCGTATCAGAAAGTGGTGGCCGGTGTCGCTAATGCGTTGGCGCACAAATACCATCATCATCATCACCACCACCATCATCATCACTAAGTCTGGTAAAGAAACCGCTGCTGCGAAATTTGAACGCCAGCACATGGACTCGTCTACTAGCGCAGCTTAATTAACCTAGGCTGCTGCCACCGCTGAGCAATAACTAGCATAACCCCTTGGGGCCTCTAAACGGGTCTTGAGGGGTTTTTTG

**3. Sequence of gene encoding rHb-CysG^A^_(G4S)3_**

Promoter: highlighted in yellow

α-globin gene coding region: red font

β-globin gene coding region: orange font

CysG^A^ gene: **Bold red font**

Linker: highlighted in gray

Terminator: highlighted in blue

TAATACGACTCACTATAGGGGAATTGTGAGCGGATAACAATTCCCCTCTAGAAATAATTTTGTTTAACTTTAAGAAGGAGATATAC**ATGGAAACGACCGAACAGTTAATCAACGAACCGCTCGACCATCGCGGTGAAGTGGTGCTGGTTGGTGCAGGTCCGGGCGATGCCGGGCTGCTGACACTGAAAGGACTGCAACAAATTCAGCAGGCAGATGTGGTGGTCTACGACCGTCTGGTTTCTGACGATATTATGAATCTGGTACGCCGCGATGCGGACCGTGTTTTCGTCGGCAAACGCGCGGGATACCACTGCGTACCCCAGGAAGAGATTAACCAGATCCTGCTGCGGGAAGCGCAAAAAGGCAAACGCGTGGTGCGGCTGAAAGGTGGCGATCCGTTTATTTTTGGCCGTGGTGGCGAAGAGCTGGAAACACTGTGCAACGCGGGTATTCCGTTCTCGGTGGTTCCGGGTATTACCGCAGCTTCTGGTTGCTCTGCCTATTCGGGTATTCCACTCACGCATCGCGATTATGCCCAGAGCGTACGCTTAATTACCGGACACTTAAAAACCGGTGGC**AGCTCAGGCAGCAGCGGTATGGTACTATCACCCGCTGATAAAACAAATGTTAAGGCGGCTTGGGGTAAGGTGGGTGCGCACGCCGGTGAATATGGTGCTGAGGCGCTTGAGCGCATGTTCCTGTCTTTTCCAACGACCAAAACCTACTTCCCGCATTTTGACCTGTCCCACGGCAGCGCACAGGTCAAAGGCCATGGCAAGAAGGTCGCAGATGCATTGACGAACGCGGTGGCCCACGTTGACGACATGCCGAATGCATTGTCGGCTCTCTCTGATCTGCATGCGCACAAACTGCGTGTGGACCCGGTAAACTTTAAGTTGCTGAGCCACTGCCTGTTGGTTACCCTGGCCGCGCACCTGCCGGCGGAATTCACTCCGGCGGTTCATGCGAGCCTGGATAAATTCCTGGCTTCCGTGAGCACCGTTCTGACCAGCAAATACCGTTAAGCATAATGCTTAAGTCGAACAGAAAGTAATCGTATTGTACACGGCCGCATAATCGAAATTAATACGACTCACTATAGGGGAATTGTGAGCGGATAACAATTCCCCATCTTAGTATATTAGTTAAGTATAAGAAGGAGATATACAATGGTACACCTAACACCCGAGGAAAAATCAGCTGTGACTGCGCTGTGGGGTAAGGTGAATGTTGACGAGGTTGGTGGTGAAGCACTGGGTCGTTTGCTGGTCGTGTACCCGTGGACCCAACGTTTTTTCGAGAGCTTCGGCGATCTGTCCACCCCGGATGCAGTTATGGGTAACCCGAAAGTAAAAGCGCACGGCAAGAAGGTGTTGGGCGCATTTAGCGACGGCCTGGCCCACCTGGACAACCTGAAAGGTACGTTTGCGACCCTCTCTGAACTGCACTGCGACAAGCTGCATGTTGATCCGGAAAACTTCCGCCTGCTTGGCAACGTGTTAGTTTGTGTTTTGGCGCACCATTTTGGCAAGGAGTTCACCCCACCGGTTCAGGCTGCGTATCAGAAAGTGGTGGCCGGTGTCGCTAATGCGTTGGCGCACAAATACCATGGAGGTGGCGGATCTGGTGGTGGCGGTTCAGGCGGCGGGGGTAGC**GAGCTGGACTGGGAAAACCTGGCGGCAGAAAAACAGACGCTGGTGTTCTATATGGGGTTGAATCAGGCCGCGACTATTCAGCAAAAGCTGATTGAACACGGAATGCCAGGCGAAATGCCGGTGGCAATTGTCGAAAACGGTACGGCAGTCACGCAGCGCGTGATTGACGGTACGCTCACACAGCTGGGAGAACTGGCGCAGCAAATGAACAGTCCATCGCTAATTATTATTGGTCGGGTTGTTGGCCTGCGCGATAAACTGAACTGGTTCTCCAACCATTAA**GTCTGGTAAAGAAACCGCTGCTGCGAAATTTGAACGCCAGCACATGGACTCGTCTACTAGCGCAGCTTAATTAACCTAGGCTGCTGCCACCGCTGAGCAATAACTAGCATAACCCCTTGGGGCCTCTAAACGGGTCTTGAGGGGTTTTTTG

**4. Sequence of gene encoding rHb_6th_ (α-G25C/G57K/P114I, β-H2E/H97W/**

**N102T) expressed in *E. coil***

Promoter: highlighted in yellow

α-globin gene coding region: red font

β-globin gene coding region: orange font

Mutant sites: underlined

His Tag: highlighted in green

Terminator: highlighted in blue

TAATACGACTCACTATAGGGGAATTGTGAGCGGATAACAATTCCCCTCTAGAAATAATTTTGTTTAACTTTAAGAAGGAGATATACATGGTACTATCACCCGCTGATAAAACAAATGTTAAGGCGGCTTGGGGTAAGGTGGGTGCGCACGCCGGTGAATATTGTGCTGAGGCGCTTGAGCGCATGTTCCTGTCTTTTCCAACGACCAAAACCTACTTCCCGCATTTTGACCTGTCCCACGGCAGCGCACAGGTCAAAAAGCATGGCAAGAAGGTCGCAGATGCATTGACGAACGCGGTGGCCCACGTTGACGACATGCCGAATGCATTGTCGGCTCTCTCTGATCTGCATGCGCACAAACTGCGTGTGGACCCGGTAAACTTTAAGTTGCTGAGCCACTGCCTGTTGGTTACCCTGGCCGCGCACCTGATTGCGGAATTCACTCCGGCGGTTCATGCGAGCCTGGATAAATTCCTGGCTTCCGTGAGCACCGTTCTGACCAGCAAATACCGTTAAGCATAATGCTTAAGTCGAACAGAAAGTAATCGTATTGTACACGGCCGCATAATCGAAATTAATACGACTCACTATAGGGGAATTGTGAGCGGATAACAATTCCCCATCTTAGTATATTAGTTAAGTATAAGAAGGAGATATACAATGGTAGAGCTAACACCCGAGGAAAAATCAGCTGTGACTGCGCTGTGGGGTAAGGTGAATGTTGACGAGGTTGGTGGTGAAGCACTGGGTCGTTTGCTGGTCGTGTACCCGTGGACCCAACGTTTTTTCGAGAGCTTCGGCGATCTGTCCACCCCGGATGCAGTTATGGGTAACCCGAAAGTAAAAGCGCACGGCAAGAAGGTGTTGGGCGCATTTAGCGACGGCCTGGCCCACCTGGACAACCTGAAAGGTACGTTTGCGACCCTCTCTGAACTGCACTGCGACAAGCTGTGGGTTGATCCGGAAACTTTCCGCCTGCTTGGCAACGTGTTAGTTTGTGTTTTGGCGCACCATTTTGGCAAGGAGTTCACCCCACCGGTTCAGGCTGCGTATCAGAAAGTGGTGGCCGGTGTCGCTAATGCGTTGGCGCACAAATACCATCATCATCATCACCACCACCATCATCATCACTAAGTCTGGTAAAGAAACCGCTGCTGCGAAATTTGAACGCCAGCACATGGACTCGTCTACTAGCGCAGCTTAATTAACCTAGGCTGCTGCCACCGCTGAGCAATAACTAGCATAACCCCTTGGGGCCTCTAAACGGGTCTTGAGGGGTTTTTTG

**5. Sequence of** **gene encoding rHb-HS1_M7A_**

mKATE2 gene coding region: red font

eGFP gene coding region: green font

cytochrome *b*_562_: blue font

Mutant sites: underlined

Linker: highlighted in gray

ATGGTTTCTGAACTGATCAAAGAAAACATGCACATGAAACTGTACATGGAAGGTACCGTTAACAACCACCACTTCAAATGCACCTCTGAAGGTGAAGGTAAACCGTACGAAGGTACCCAGACCATGCGTATCAAAGCTGTTGAAGGTGGTCCGCTGCCGTTCGCTTTTGACATCCTGGCTACCTCTTTCATGTACGGTTCTAAAACCTTCATCAACCACACCCAGGGTATCCCGGACTTTTTCAAACAGTCTTTCCCGGAAGGTTTCACCTGGGAACGTGTTACCACCTACGAAGACGGTGGTGTTCTGACCGCTACCCAGGACACCTCTCTGCAAGACGGTTGCCTGATCTACAACGTTAAAATCCGTGGTGTTAACTTCCCGTCTAACGGTCCGGTTATGCAGAAAAAAACCCTGGGTTGGGAAGCTTCTACCGAAACCCTGTACCCGGCTGACGGTGGTCTGGAAGGTCGTGCTGACATGGCTCTGAAACTGGTTGGTGGTGGTCACCTGATCTGCAACCTGAAAACCACCTACCGTTCTAAAAAACCGGCTAAAAACCTGAAAATGCCGGGTGTTTACTACGTTGACCGTCGTCTGGAACGTATCAAAGAAGCTGACAAAGAAACCTACGTTGAACAGCACGAAGTTGCTGTTGCTCGTTACTGCGACCTGCCGTCTAAACTGGGTCACCGTGGGGGCAGCATGGGTAAGGGAGAAGAACTTTTCACTGGAGTTGTCCCAATTCTTGTTGAATTAGATGGTGATGTTAATGGGCACAAATTTTCTGTCAGTGGAGAGGGTGAAGGTGATGCAACATACGGTGGCTCTGCTGATCTTGAAGACAATGCTGAAACCCTCAACGACAATTTAAAAGTGATCGAAAAAGCGGATAACGCGGCGCAAGTCAAAGACGCGTTAACGAAGATGCGCGCCGCAGCGCTGGATGCGCAAAAAGCAACGCCGCCGAAGCTCGAAGATAAATCACCGGACAGCCCGGAAATGAAAGATTTCCGCCACGGTTTCGACATTCTGGTCGGTCAGATTGACGACGCGCTGAAGCTGGCAAATGAAGGTAAAGTAAAAGAAGCGCAGGCTGCTGCAGAGCAACTGAAAACGACCCGCAACGCCTATCACCAGAAGTATCGTGGTGGAAAACTTACCCTTAAATTTATTTGCACTACTGGAAAGCTTCCTGTTCCTTGGCCAACACTTGTCACTACTCTTACTTATGGTGTTCAATGCTTTTCAAGATACCCAGATCATATGAAGCGGCACGACTTCTTCAAGAGCGCCATGCCTGAGGGATACGTGCAGGAGAGGACCATCTTCTTCAAGGACGACGGGAACTACAAGACACGTGCTGAAGTCAAGTTTGAGGGAGACACCCTCGTCAACAGAATCGAGCTTAAGGGAATCGATTTCAAGGAGGACGGAAACATCCTCGGCCACAAGTTGGAATACAACTACAACTCCCACAACGTATACATCATGGCAGACAAACAAAAGAATGGAATCAAAGTTAACTTCAAAATTAGACACAACATTGAAGATGGAAGCGTTCAACTAGCAGACCATTATCAACAAAATACTCCAATTGGCGATGGCCCTGTCCTTTTACCAGACAACCATTACCTGTCCACACAATCTGCCCTTTCGAAAGATCCCAACGAAAAGAGAGACCACATGGTCCTTCTTGAGTTTGTAACAGCTGCTGGGATTACACATGGCATGGATGAACTATACAAATAA

**6. Sequence of gene encoding rHb_9th_ (α-G25C/Y42S/G57K/P114I, β-H2E/S44D/P51N/H97W/N102T) expressed in *E. coil***

Promoter: highlighted in yellow

α-globin gene coding region: red font

β-globin gene coding region: orange font

Mutant sites: underlined

His Tag: highlighted in green

Terminator: highlighted in blue

TAATACGACTCACTATAGGGGAATTGTGAGCGGATAACAATTCCCCTCTAGAAATAATTTTGTTTAACTTTAAGAAGGAGATATACATGGTACTATCACCCGCTGATAAAACAAATGTTAAGGCGGCTTGGGGTAAGGTGGGTGCGCACGCCGGTGAATATTGTGCTGAGGCGCTTGAGCGCATGTTCCTGTCTTTTCCAACGACCAAAACCAGTTTCCCGCATTTTGACCTGTCCCACGGCAGCGCACAGGTCAAAAAGCATGGCAAGAAGGTCGCAGATGCATTGACGAACGCGGTGGCCCACGTTGACGACATGCCGAATGCATTGTCGGCTCTCTCTGATCTGCATGCGCACAAACTGCGTGTGGACCCGGTAAACTTTAAGTTGCTGAGCCACTGCCTGTTGGTTACCCTGGCCGCGCACCTGATTGCGGAATTCACTCCGGCGGTTCATGCGAGCCTGGATAAATTCCTGGCTTCCGTGAGCACCGTTCTGACCAGCAAATACCGTTAAGCATAATGCTTAAGTCGAACAGAAAGTAATCGTATTGTACACGGCCGCATAATCGAAATTAATACGACTCACTATAGGGGAATTGTGAGCGGATAACAATTCCCCATCTTAGTATATTAGTTAAGTATAAGAAGGAGATATACAATGGTAGAGCTAACACCCGAGGAAAAATCAGCTGTGACTGCGCTGTGGGGTAAGGTGAATGTTGACGAGGTTGGTGGTGAAGCACTGGGTCGTTTGCTGGTCGTGTACCCGTGGACCCAACGTTTTTTCGAGGATTTCGGCGATCTGTCCACCAATGATGCAGTTATGGGTAACCCGAAAGTAAAAGCGCACGGCAAGAAGGTGTTGGGCGCATTTAGCGACGGCCTGGCCCACCTGGACAACCTGAAAGGTACGTTTGCGACCCTCTCTGAACTGCACTGCGACAAGCTGTGGGTTGATCCGGAAACTTTCCGCCTGCTTGGCAACGTGTTAGTTTGTGTTTTGGCGCACCATTTTGGCAAGGAGTTCACCCCACCGGTTCAGGCTGCGTATCAGAAAGTGGTGGCCGGTGTCGCTAATGCGTTGGCGCACAAATACCATCATCATCATCACCACCACCATCATCATCACTAAGTCTGGTAAAGAAACCGCTGCTGCGAAATTTGAACGCCAGCACATGGACTCGTCTACTAGCGCAGCTTAATTAACCTAGGCTGCTGCCACCGCTGAGCAATAACTAGCATAACCCCTTGGGGCCTCTAAACGGGTCTTGAGGGGTTTTTTG

**7. Sequence of gene encoding sperm whale apomyoglobin_H64Y/V68F_ expressed in *E. coil***

Promoter: highlighted in yellow

Gene coding region: red font

Mutant sites: underlined

His Tag: highlighted in green

Terminator: highlighted in blue

TAATACGACTCACTATAGGGGAATTGTGAGCGGATAACAATTCCCCTCTAGAAATAATTTTGTTTAACTTTAAGAAGGAGATATACCATGGTGCTGAGCGAAGGCGAATGGCAGCTGGTGCTGCATGTGTGGGCGAAAGTGGAAGCGGATGTGGCGGGCCATGGCCAAGATATTCTGATTCGCCTGTTTAAAAGCCATCCGGAAACCCTGGAAAAATTTGATCGCTTTAAACATCTGAAAACCGAAGCGGAAATGAAAGCGAGCGAAGATCTGAAAAAATATGGCGTGACCTTTCTGACCGCGCTGGGCGCGATTCTGAAAAAGAAAGGCCATCATGAAGCGGAACTGAAACCGCTGGCGCAGAGCCATGCGACCAAACATAAAATTCCGATTAAATATCTGGAATTTATTAGCGAAGCGATTATTCATGTGCTGCATAGCCGCCATCCGGGCGATTTTGGCGCGGATGCGCAAGGCGCGATGAACAAAGCGCTGGAACTGTTTCGCAAAGATATTGCGGCGAAATATAAAGAACTGGGCTATCAAGGCCATCATCATCACCACCACCATCATCATCACTAAATAACTAGCATAACCCCTTGGGGCCTCTAAACGGGTCTTGAGGGGTTTTTTG

**8. Sequence of gene encoding rHb_13th_ (α-V1E/G25C/Y42S/G57K/P114I, β-H2E/S44D/P51N/H97W/N102T/N108K) expressed in *E. coil***

Promoter: highlighted in yellow

α-globin gene coding region: red font

β-globin gene coding region: orange font

Mutant sites: underlined

His Tag: highlighted in green

Terminator: highlighted in blue

TAATACGACTCACTATAGGGGAATTGTGAGCGGATAACAATTCCCCTCTAGAAATAATTTTGTTTAACTTTAAGAAGGAGATATACATGGAACTATCACCCGCTGATAAAACAAATGTTAAGGCGGCTTGGGGTAAGGTGGGTGCGCACGCCGGTGAATATTGTGCTGAGGCGCTTGAGCGCATGTTCCTGTCTTTTCCAACGACCAAAACCAGTTTCCCGCATTTTGACCTGTCCCACGGCAGCGCACAGGTCAAAAAGCATGGCAAGAAGGTCGCAGATGCATTGACGAACGCGGTGGCCCACGTTGACGACATGCCGAATGCATTGTCGGCTCTCTCTGATCTGCATGCGCACAAACTGCGTGTGGACCCGGTAAACTTTAAGTTGCTGAGCCACTGCCTGTTGGTTACCCTGGCCGCGCACCTGATTGCGGAATTCACTCCGGCGGTTCATGCGAGCCTGGATAAATTCCTGGCTTCCGTGAGCACCGTTCTGACCAGCAAATACCGTTAAGCATAATGCTTAAGTCGAACAGAAAGTAATCGTATTGTACACGGCCGCATAATCGAAATTAATACGACTCACTATAGGGGAATTGTGAGCGGATAACAATTCCCCATCTTAGTATATTAGTTAAGTATAAGAAGGAGATATACAATGGTAGAGCTAACACCCGAGGAAAAATCAGCTGTGACTGCGCTGTGGGGTAAGGTGAATGTTGACGAGGTTGGTGGTGAAGCACTGGGTCGTTTGCTGGTCGTGTACCCGTGGACCCAACGTTTTTTCGAGGATTTCGGCGATCTGTCCACCAATGATGCAGTTATGGGTAACCCGAAAGTAAAAGCGCACGGCAAGAAGGTGTTGGGCGCATTTAGCGACGGCCTGGCCCACCTGGACAACCTGAAAGGTACGTTTGCGACCCTCTCTGAACTGCACTGCGACAAGCTGTGGGTTGATCCGGAAACTTTCCGCCTGCTTGGCAAAGTGTTAGTTTGTGTTTTGGCGCACCATTTTGGCAAGGAGTTCACCCCACCGGTTCAGGCTGCGTATCAGAAAGTGGTGGCCGGTGTCGCTAATGCGTTGGCGCACAAATACCATCATCATCATCACCACCACCATCATCATCACTAAGTCTGGTAAAGAAACCGCTGCTGCGAAATTTGAACGCCAGCACATGGACTCGTCTACTAGCGCAGCTTAATTAACCTAGGCTGCTGCCACCGCTGAGCAATAACTAGCATAACCCCTTGGGGCCTCTAAACGGGTCTTGAGGGGTTTTTTG

**9. Sequence of gene encoding HrtR_L_**

ATGCCCAAATCTACATATTTTAGTCTATCAGACGAGAAACGTAAACGTGTTTACGATGCGTGCCTGCTGGAGTTTCAGACCCACTCGTTCCATGAGGCTAAAATCATGCACATCGTGAAAGCACTGGACATCCCGCGTGGTAGCTTCTATCAGTACTTTGAAGATCTTAAGGACAGCTATTACTACATTCTGTCCCAAGAAACGGTTGAAATTCATGATCTGTTTTTCAACCTGTTGAAGGAGTACCCGCTGGAGGTTGCCCTCAACAAGTACAAGTATCTGTTACTGGAAAATTTGGTTAATTCTCCACAGTATAACCTGTATAAGTACCGCTTTCTGGACTGGACTTATGAACTGGAACGTGATTGGAAACCGAAAGGTGAAGTAACCGTGCCGGCGAGAGAACTGGACAACCCGATTTCCCAAGTGCTGAAGAGCGTTATTAGTAATCTGGTCTATCGCATGTTCAGCGAAAACTGGGATGAGCAAAAATTCATCGAGACGTACGACAAGGAGATCAAACTGTTGACCGAGGGCTTGTTGAACTACGTGACCGAGAGCAAAAAGTAA

**References**

Bisse, E., Schaeffer-Reiss, C., Van Dorsselaer, A., Alayi, T.D., Epting, T., Winkler, K., et al. (2017) Hemoglobin Kirklareli (alpha H58L), a new variant associated with iron deficiency and increased CO binding. *J Biol Chem* 292: 2542-2555.

Bissé, E., Schaeffer-Reiss, C., Van Dorsselaer, A., Alayi, T.D., Epting, T., Winkler, K., et al. (2017) Hemoglobin Kirklareli (α H58L), a new variant associated with Iron deficiency and increased CO binding. *J Biol Chem* 292: 2542-2555.

Cooper, C.E., Silkstone, G.G.A., Simons, M., Rajagopal, B., Syrett, N., Shaik, T., et al. (2019) Engineering tyrosine residues into hemoglobin enhances heme reduction, decreases oxidative stress and increases vascular retention of a hemoglobin based blood substitute. *Free Radic Biol Med* 134: 106-118.

Correddu, D., Catucci, G., Giuriato, D., Di Nardo, G., Ciaramella, A., and Gilardi, G. (2023) Catalytically self-sufficient CYP116B5: Domain switch for improved peroxygenase activity, *Biotechnol J* 18: e2200622.

de Weinstein, B.I., Plaseska-Karanfilska, D., and Efremov, G.D. (2000) Hb saint etienne or Hb istanbul [β92(F8)His→Gln] found in an Argentinean family. *Hemoglobin* 24: 149-152.

Dou, Y., Maillett, D.H., Eich, R.F., and Olson, J.S. (2002) Myoglobin as a model system for designing heme protein based blood substitutes. *Biophys Chem* 98: 127-148.

Gattoni, M., Piro, M.C., Boffi, A., Brinigar, W.S., Fronticelli, C., and Chiancone, E. (2001) The heme-globin and dimerization equilibria of recombinant human hemoglobins carrying site-specific beta chains mutations. *Arch Biochem Biophys* 386: 172-178.

Graves, P.E., Henderson, D.P., Horstman, M.J., Solomon, B.J., and Olson, J.S. (2008) Enhancing stability and expression of recombinant human hemoglobin in *E. coli*: Progress in the development of a recombinant HBOC source. *Biochim Biophys Acta* 1784: 1471-1479.

Hu, B.D., Yu, H.B., Zhou, J.W., Li, J.H., Chen, J., Du, G.C., et al. (2023) Whole-cell P450 biocatalysis using engineered with fine-tuned heme biosynthesis. *Adv Sci* 10: e2205580.

Kanagarajan, S., Carlsson, M.L.R., Chakane, S., Kettisen, K., Smeds, E., Kumar, R., et al. (2021) Production of functional human fetal hemoglobin in Nicotiana benthamiana for development of hemoglobin-based oxygen carriers. *Int J Biol Macromol* 184: 955-966.

Looker, D., Abbottbrown, D., Cozart, P., Durfee, S., Hoffman, S., Mathews, A.J., et al. (1992) A human recombinant hemoglobin designed for use as a blood substitute. *Nature* 356: 258-260.

Natarajan, C., Signore, A.V., Kumar, V., and Storz, J.F. (2020) Synthesis of recombinant human hemoglobin with NH(2) -terminal acetylation in *Escherichia coli*. *Curr Protoc Protein Sci* 101: e112.

Okamoto, Y., Sugisaki, S., Suga, K., and Umakoshi, H. (2017) Development of time-course oxygen binding analysis for hemoglobin-based oxygen carriers. *Anal Sci* 33: 953-956.

Pires, I.S., Belcher, D.A., Hickey, R., Miller, C., Badu-Tawiah, A.K., Baek, J.H., et al. (2020) Novel manufacturing method for producing apohemoglobin and its biophysical properties. *Biotechnol* 117: 125-145.

Samuel, P.P., Ou, W.C., Phillips, G.N., Jr. &, and Olson, J.S. (2017) Mechanism of human apohemoglobin unfolding. *Biochem* 56: 1444-1459.

Silkstone, G.G.A., Silkstone, R.S., Wilson, M.T., Simons, M., Bülow, L., Kallberg, K., et al. (2016) Engineering tyrosine electron transfer pathways decreases oxidative toxicity in hemoglobin: implications for blood substitute design. *Biochem J* 473: 3371-3383.

Tupta, B., Stuehr, E., Sumi, M.P., Sweeny, E.A., Smith, B., Stuehr, D.J., and Ghosh, A. (2022) GAPDH is involved in the heme-maturation of myoglobin and hemoglobin. *Faseb Journal* 36: e22099.

Varnado, C.L., Mollan, T.L., Birukou, I., Henderson, D.P., and Olson, J.S. (2013) Development of recombinant hemoglobin-based oxygen carriers. *Antioxid Redox Signal* 18: 2314-2328.

Yan, W., Shen, L., Yu, W., Zhou, H., and Hu, T. (2020) A triply modified human adult hemoglobin with low oxygen affinity, rapid autoxidation and high tetramer stability. *Int J Biol Macromol* 159: 236-242.
